# Probe metal binding mode of imine covalent organic frameworks: cycloiridation for (photo)catalytic hydrogen evolution from formate†

**DOI:** 10.1039/d1sc01692j

**Published:** 2021-05-11

**Authors:** Jiyun Hu, Hamed Mehrabi, Yin-Shan Meng, Maddison Taylor, Jin-Hui Zhan, Qigeng Yan, Mourad Benamara, Robert H. Coridan, Hudson Beyzavi

**Affiliations:** Department of Chemistry and Biochemistry, University of Arkansas Fayetteville Arkansas 72701 USA beyzavi@uark.edu; Material Science and Engineering Program, University of Arkansas Fayetteville Arkansas 72701 USA; State Key Laboratory of Fine Chemicals, Dalian University of Technology Dalian 116024 China; State Key Laboratory of Multiphase Complex System, Institute of Process Engineering, Chinese Academy of Sciences Beijing 100190 China jhzhan@ipe.ac.cn; Institute for Nanoscience & Engineering, University of Arkansas Fayetteville Arkansas 72701 USA

## Abstract

Metalation of covalent organic frameworks (COFs) is a critical strategy to functionalize COFs for advanced applications yet largely relies on the pre-installed specific metal docking sites in the network, such as porphyrin, salen, 2,2′-bipyridine, *etc.* We show in this study that the imine linkage of simple imine-based COFs, one of the most popular COFs, readily chelate transition metal (Ir in this work) *via* cyclometalation, which has not been explored before. The iridacycle decorated COF exhibited more than 10-fold efficiency enhancement in (photo)catalytic hydrogen evolution from aqueous formate solution than its molecular counterpart under mild conditions. This work will inspire more functional cyclometallated COFs to be explored beyond catalysis considering the large imine COF library and the rich metallacycle chemistry.

## Abstract 2

This study describes cyclometallation as a new metal binding mode for imine-based COFs. The iridacycle decorated COF could be used for catalytic hydrogen evolution from aqueous formate solution with high stability and high efficacy.

## Graphical abstract

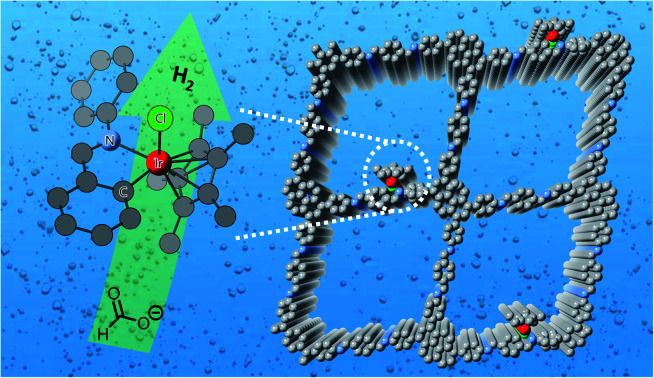

## Introduction

**Fig. 1:**
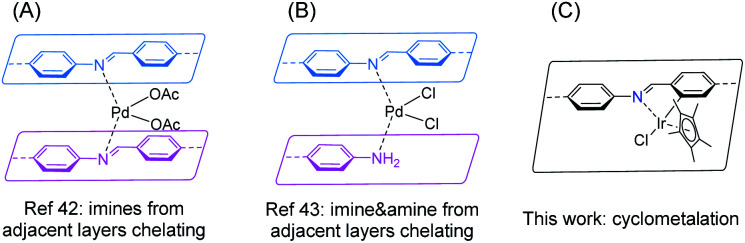
Metal binding modes of imine-based COFs.

The designable ordered porous structure of covalent organic frameworks (COFs) has attracted broad research interest in the past decades.^1–4^ Although COFs are completely composed of light elements (typically H, B, C, N, O, Si), their advanced applications, particularly catalysis,^5–8^ have stressed the role of metal species anchored onto the network.^8–10^ To accommodate the target metal, a building unit with a suitable metal binding site is required to be incorporated into the framework, which usually meets complicated organic synthesis. Nevertheless, plenty of functional linkers have been successfully implemented into COFs, such as 2,2′-bipyridine,^11–14^ phenanthroline,^15–17^ porphyrin,^18–22^ phthalocyanine,^23–25^ salen,^26–29^ hydroxy groups,^30–32^ β-ketoenamine,^33^ phosphine,^34,35^ dehydrobenzoannulene,^36,37^ among others.^9,10^ As one of the most popular COFs featuring both high crystallinity and stability, Schiff base COFs possess uniformly distributed imine linkages,^38^ which have been well studied in coordination chemistry.^39–41^ If the imine linkages can be effectively exploited for binding metals, a huge COF catalyst library would be accessible considering the large number of imine COFs. While the first COF-based heterogeneous catalyst was constructed on an imine COF, in which palladium(ii) was chelated by two imine moieties from adjacent COF layers in 2011 (Fig. 1A),^42^ not much progress has been achieved on using imines in COFs as intrinsic ligands for transition metals during the past decade. Only recently a new binding mode of stabilizing palladium(ii) with imine and amine defect from adjacent layers of an imine COF was identified (Fig. 1B).^43^ Thus, it is of great interest to explore both the scope of metal species and binding modes of imine-based COFs.

(Photo)catalytic hydrogen evolution reaction (HER) from water splitting is a promising clean energy production technique. COFs have been proved to be potential photocatalysts in promoting HER.^44–49^ The system is typically comprised of COF photocatalyst, Pt nanoparticle cocatalyst, and a sacrificial electron donor (*e.g.* ascorbic acid, triethanolamine) in an aqueous solution. Formic acid serves as an alternative high H_2_ storage density reservoir (4.3 wt%).^50,51^ The decomposition of HCOOH under basic conditions could release H_2_ with high purity. However, some iridium-based homogeneous HCOOH dehydrogenation catalysts displayed low stability and were prone to deactivate *via* nanoparticle formation.^52,53^ Immobilization of the catalyst on proper solid support is expected to extend the catalyst lifetime.^54^ Taking all of these considerations together, in this work, we show that cyclometalation at the imine site leads to an iridacycle functionalized imine COF for the first time (Fig. 1C), which exhibits fascinating performance in (photo)catalytic HER from aqueous formate solution.

## Results and discussion

### Synthesis and characterization

**Scheme 1:**
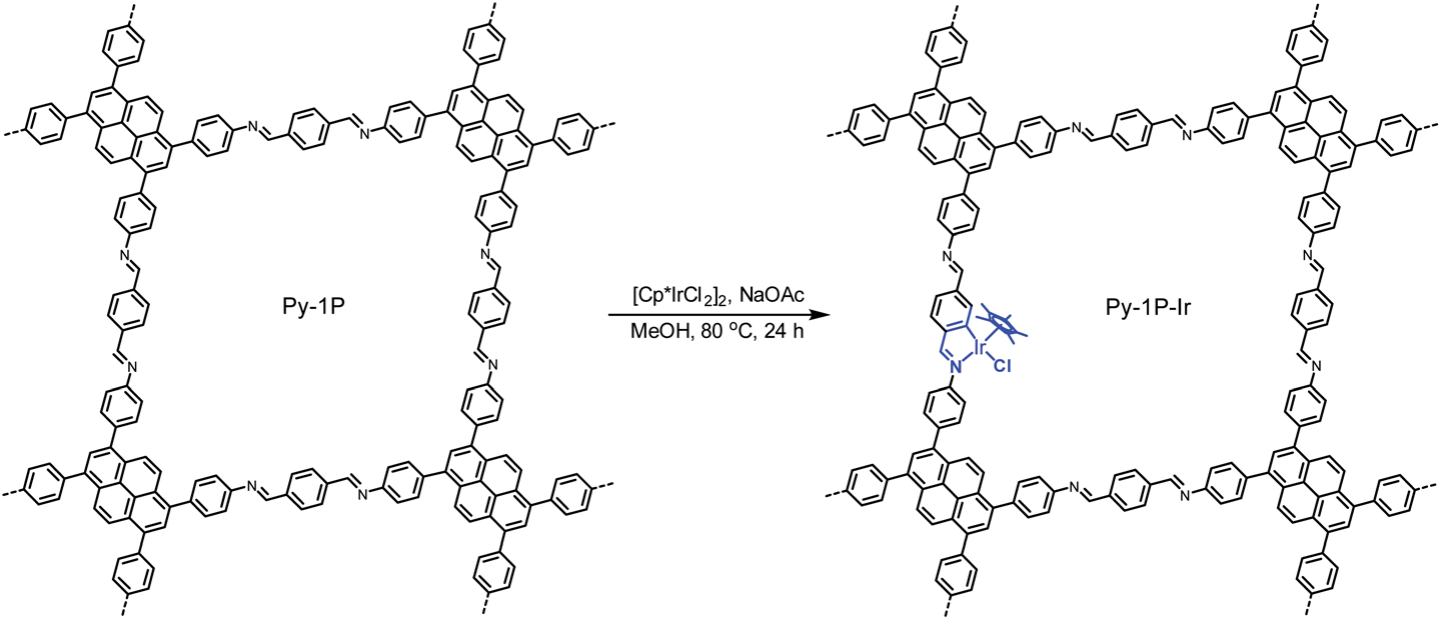
Cyclometalation of **Py-1P** COF with iridium.

Metallacycles of Schiff base ligands have been well explored for transition metals in organometallic chemistry.^55–57^ Nevertheless, introducing such type of complexes into COFs is unprecedented. To explore this new binding mode, a pyrene-based imine COF (**Py-1P**) constructed from 1,3,6,8-tetrakis(4-aminophenyl)pyrene and terephthalaldehyde was chosen as a model system because of its high crystallinity^58^ and facile synthesis.^59^ Iridium became the metal choice because of the various catalytic applications of iridacycles.^60,61^ Metalation of **Py-1P** COF was carried out by refluxing with [Cp*IrCl_2_]_2_ (Cp*, pentamethylcyclopentadienyl) in methanol in the presence of NaOAc for 24 hours (Scheme 1, see ESI† for details).

**Fig. 2:**
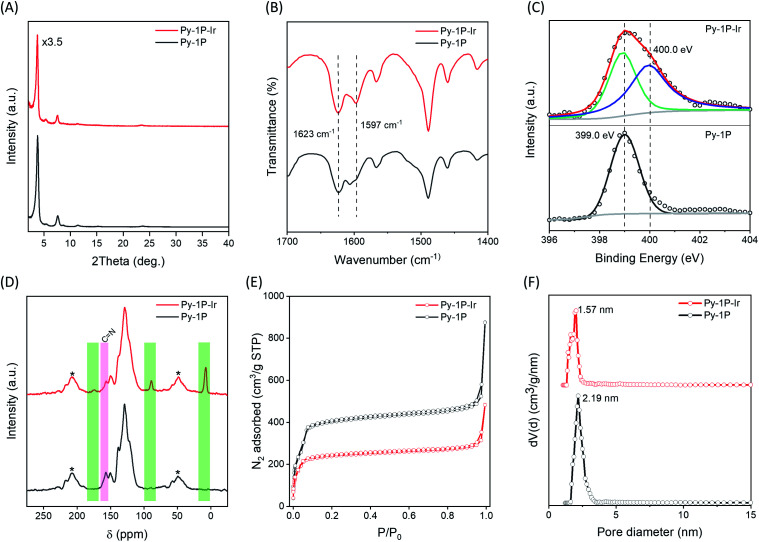
Comparison of the (A) PXRD patterns, (B) FT-IR spectra, (C) N 1s XPS spectra, (D) CP/MAS ^13^C NMR spectra, (E) N_2_ adsorption–desorption isotherms, and (F) pore size distributions of **Py-1P** and **Py-1P–Ir** COFs. Pore size distribution was analysed using the nonlocal density functional theory equilibrium model. The NMR spinning sidebands in (D) are marked with asterisks (*).

The successful formation of the iridacycle functionalized COF **Py-1P–Ir** was confirmed by FT-IR spectroscopy, X-ray photoelectron spectroscopy (XPS), and solid-state NMR characterization, and the retention of the framework crystallinity and porosity was examined by powder X-ray diffraction (PXRD) and N_2_ adsorption–desorption isotherm analysis, respectively. **Py-1P–Ir** COF showed strong diffraction peaks at 2*θ* = 3.74, 5.36, 7.56, 11.40, 23.52°, which are slightly shifted to low 2*θ* direction (0.04 to 0.18° difference) compared to the diffraction pattern of the parent **Py-1P** COF (Fig. 2A). This might result from the crystal cell expansion after introducing the bulky Ir organometallic unit. FT-IR analysis revealed a new C=N vibration band at 1597 cm^−1^, corresponding to the metal coordinated imines, in addition to the free ones at 1623 cm^−1^ for **Py-1P–Ir** COF and (Fig. 2B). The shifting of C=N vibration to lower wavenumber upon coordination to Ir was also observed for *N*-benzylideneaniline (**L1**) when forming the model iridacycle complex (**L1–Ir**, 1626 to 1582 cm^−1^, Fig. S1†).^62^ The coordination of imine N to iridium was further proved by XPS analysis (Fig. S2† and 2C). The N 1s XPS spectrum of **Py-1P–Ir** COF showed two subpeaks with binding energies of 399.0 and 400.0 eV, which were assigned to the free and coordinated imine nitrogen, respectively (Fig. 2C). The increase of N 1s binding energy upon coordination is consistent with that of the model complex **L1–Ir** (399.2 to 399.8 eV, Fig. S3†). Solid-state cross-polarization/magic angle spinning (CP/MAS) ^13^C NMR spectroscopy unambiguously confirmed the formation of the iridacycle in **Py-1P–Ir** COF. Three new signals at 173.7, 89.0, and 7.6 ppm appeared in the CP/MAS ^13^C NMR spectrum of **Py-1P–Ir** COF (Fig. 2D). The broad peak at 173.7 ppm is assigned to the iridium bonded carbon and the imine carbon of the iridacycle, while the peaks at 89.0 and 7.6 ppm originate from the aromatic and methyl carbons of Cp* ring, respectively, which matches well with the ^13^C NMR spectrum of the model complex **L1–Ir** (Fig. S4†). Meanwhile, the relative intensity of the free imine carbon peak at 157.0 ppm of **Py-1P–Ir** COF decreased accordingly compared to that of **Py-1P** COF. N_2_ adsorption experiment was carried out to investigate the porosity of **Py-1P–Ir** COF (Fig. 2E). The Brunauer–Emmett–Teller (BET) surface area of **Py-1P–Ir** COF is 972 m^2^ g^−1^, which is lower than 1960 m^2^ g^−1^ of **Py-1P** COF (Fig. S5 and S6†). The pore size is decreased to 1.57 nm for **Py-1P–Ir** COF, compared to 2.19 nm for **Py-1P** COF, which is expected for the pore wall metalation (Fig. 2F). The iridium loading was determined to be 11.8 wt% by inductively coupled plasma mass spectrometry (ICP-MS), corresponding to *ca.* 20% imine metalation. Scanning electron microscope analysis revealed that **Py-1P–Ir** COF was composed of aggregates of nanometer-sized particles, similar to that of **Py-1P** COF (Fig. S7†). Energy dispersive X-ray (EDX) analysis revealed the homogeneous distributions of Ir and Cl elements over the framework, and the Ir/Cl ratio is close to 1/1, as expected for the proposed structure (Fig. S8†). Besides, the formation of the iridacycle was also successfully performed on an azine linked COF (see ESI† for details), demonstrating the generality of the cyclometalation modification of imine-based COFs.

### Catalytic HER from formate

**Fig. 3:**
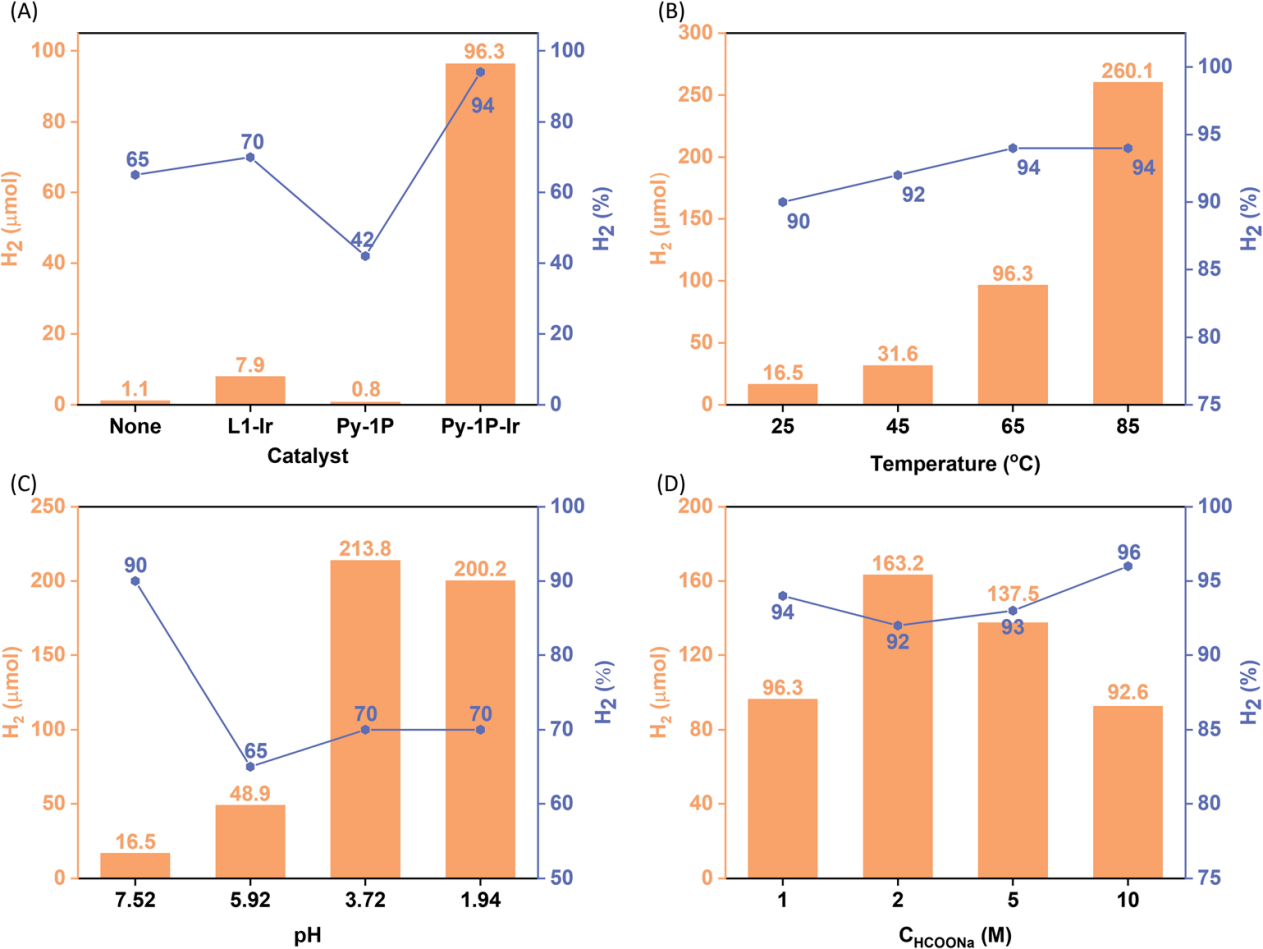
Catalytic hydrogen evolution from aqueous formate solution. The influence of catalyst (A), temperature (B), pH (C), and formate concentration (D) on the reaction outcome (H_2_ quantity and purity are presented in orange column and grey line/square symbol form, respectively. Purity refers to the H_2_ composition in the generated H_2_/CO_2_ mixture). Standard reaction condition: the reactions were carried out by heating a 10 mL HCOONa solution (1.0 M, pH = 7.52) containing 6.35 μmol catalyst (based on Ir) at 65 °C for 6 h. The pH effect was studied at room temperature. The concentration effect was studied at 65 °C and the pHs of HCOONa solutions were not adjusted. See ESI† for details.

Iridium complexes have demonstrated their potential to be homogeneous catalysts for hydrogen production from formate.^50–53,63^ The homogeneous distribution of the single-site iridacycle over the **Py-1P–Ir** COF with high surface area prompted us to test its capability in promoting formate decomposition to release H_2_. To our delight, **Py-1P–Ir** COF produced 96.3 μmol H_2_ with a purity of 94% from 10 mL of 1.0 M HCOONa solution at 65 °C in 6 hours (6.35 μmol catalyst based on Ir, Fig. 3A). In contrast, **L1–Ir** gave only 7.9 μmol H_2_ with 70% purity under otherwise identical conditions. **Py-1P** COF was catalytically inactive, showing even lower H_2_ production (0.8 μmol, 42%) than the control reaction (2.3 μmol, 65%). These results highlight the importance of the unique structure of **Py-1P–Ir** COF. Then, we set to investigate the influence of reaction parameters on the hydrogen production efficiency catalyzed by **Py-1P–Ir** COF, including temperature, pH, and formate concentration.

**Fig. 4:**
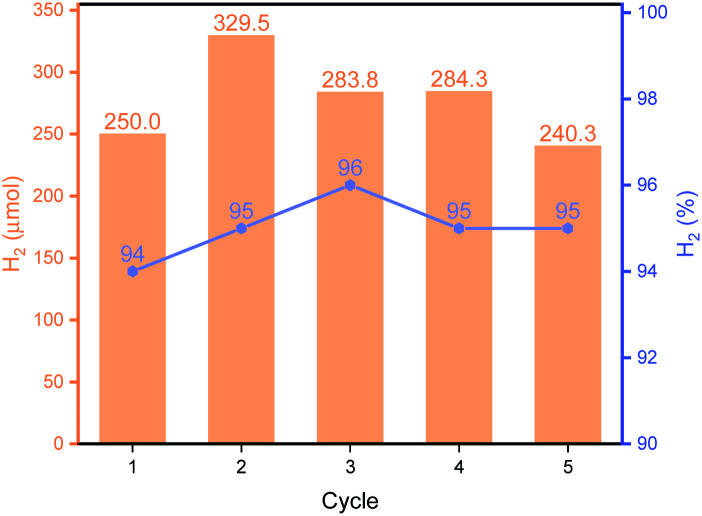
Recyclability of **Py-1P–Ir** COF. The reactions were carried out in 1 M HCOONa at 85 °C for 6 hours.

As shown in Fig. 3B, **Py-1P–Ir** COF displayed higher efficiency at higher temperature. An amount of 250.0 μmol H_2_ was obtained at 85 °C, which is *ca.* 2.6 times of that obtained at 65 °C. The high reactivity of **Py-1P–Ir** COF was manifested that 16.5 μmol H_2_ was formed even at 25 °C. In all the tested temperatures, the H_2_ purity was no less than 90%. Lowering the pH was found to favor the reaction that 48.9 and 213.8 μmol H_2_ were afforded at pH = 5.92, 3.72 respectively, while further lowering the pH to 1.94 did not lead to higher H_2_ production (200.2 μmol, Fig. 3C). The increased H_2_ production came with compromised H_2_ purity to *ca.* 70% under acidic conditions. The concentration of HCOONa on the HER showed a volcano-type effect (Fig. 3D). The H_2_ yield increased to 163.2 μmol from the reaction of 2 M HCOONa but started to fall back to 137.5 μmol for 5 M HCOONa and 92.6 μmol for 5 M HCOONa respectively thereafter. The H_2_ purities were all higher than 90% from the tested four HCOONa concentrations. The decreased reaction efficiency might be due to the decreased concentration of water, which is the proton source for H_2_ production. **Py-1P–Ir** COF exhibited excellent stability under all the tested conditions. All the recovered COF samples preserved the crystallinity as shown by PXRD analysis (Fig. S20–S22†). Interestingly, FT-IR and XPS indicate that the imine linkage was partially reduced (Fig. S20–S23†). The imine bond reduction is likely mediated by an iridium hydride intermediate *via* an outer-sphere process.^64–66^ The involvement of Ir in the imine reduction is supported by the fact that no imine reduction was observed in **Py-1P** COF during HER catalysis (Fig. S24†) and the catalytic reduction of *N*-benzylideneaniline as an exogenous substrate in the presence of **Py-1P–Ir** COF was observed (Fig. S25†). ICP-MS showed that the Ir concentrations in the reaction filtrates were all below 60 ppb (Table S2†), demonstrating the heterogeneous catalysis nature of the reaction. The high stability of **Py-1P–Ir** COF allowed it to be recycled for at least another four runs (Fig. 4). Interestingly, an initial performance improvement was observed in the second cycle. This improved reactivity of **Py-1P–Ir** COF is attributed to the presence of more reactive Ir species in recovered material, in which the Cl ligand has been replaced (see mechanism discussion below). This is supported by the fact that no Cl element was detected by EDX in **Py-1P–Ir** COF after one HER cycle (Fig. S26†). The average H_2_ production rate during the five cycles was calculated to be 4626 μmol g^−1^ h^−1^, corresponding to a TOF of 7.3 h^−1^. The recovered **Py-1P–Ir** COF after five cycles was still crystalline (Fig. S28†). No metal nanoparticle formation was observed by XPS (Fig. S29†) and TEM (Fig. S30†) analysis.

**Fig. 5:**
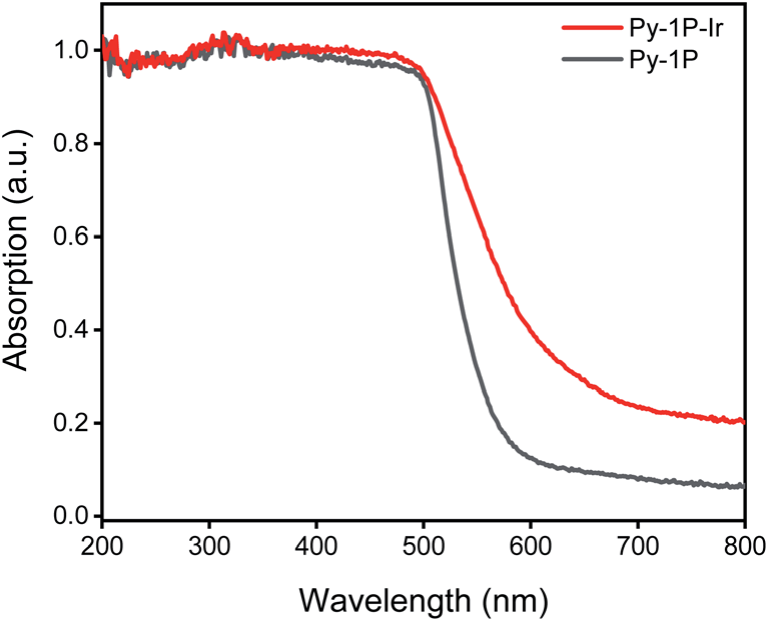
Diffuse reflectance UV-vis spectra of **Py-1P** and **Py-1P–Ir** COF.

Pyrene-based COFs have exhibited excellent photophysical properties which benefit photocatalysis.^67–70^ Diffuse reflectance UV-vis absorption spectrum of **Py-1P–Ir** COF displayed prominent absorption in 200–500 nm and a long tail to near-IR range (500–800 nm, Fig. 5). The incorporation of iridium complex into **Py-1P** COF did not change the absorption in 200–500 nm band but strongly improved the absorption ability in the 500–800 nm region. Encouraged by the excellent light absorption ability across the whole UV-visible region of **Py-1P–Ir** COF, we further tested its photocatalytic HER performance from formate decomposition. The reactions were carried out in 1 M formate solution in the presence of 6.35 μmol catalyst (based on Ir) under 460 nm light irradiation. As shown in Table 1, only 0.7 μmol H_2_ was detected in the absence of catalyst (entry 1). **Py-1P–Ir** COF catalyzed the generation of 84.5 μmol H_2_ in high purity (93%) in 6 hours (entry 3), which is *ca.* 34 times higher than the molecular counterpart **L1–Ir** did (entry 2). The corresponding H_2_ generation rate of 1358 μmol g^−1^ h^−1^ (TOF 2.2 h^−1^) is comparable to the typical COF photocatalyst/Pt cocatalyst/sacrificial electron donor system.^71^**Py-1P** COF failed to exert any catalytic effect showing the critical role of iridium for the reaction (entry 5, 0.6 μmol H_2_). Under the dark condition, **Py-1P–Ir** COF produced 31.6 μmol H_2_ in 92% purity, proving it to be a photocatalytic process (entry 4). A physical mixture of **Py-1P** COF and **L1–Ir** displayed lower H_2_ production (19.5 μmol) and poorer selectivity (71% purity), highlighting the importance of covalent hybridization of Ir catalytic centers within the COF (entry 6).^72^ The recovered **Py-1P–Ir** COF almost retained its crystalline structure as evidenced by PXRD analysis (Fig. S31†). Similarly, reduction of the imine bond was observed (Fig. S31†). ICP-MS analysis of the **Py-1P–Ir** COF catalyzed reaction solution revealed a low Ir concentration of 0.99 ppm, corresponding to 0.8% of the total Ir in **Py-1P–Ir** COF. Besides, the reaction filtrate only produced 2.0 μmol H_2_ under the photocatalysis conditions (entry 7), demonstrating the heterogeneous catalytic process by **Py-1P–Ir** COF.

**Table 1:** Photocatalytic HER from aqueous formate solution^a^

Entry	Catalyst	H_2_ (μmol)	Purity (%)
1	—	0.7	10
2	**L1–Ir**	2.5	82
3	**Py-1P–Ir**	84.5	93
4^b^	**Py-1P–Ir**	31.6	92
5	**Py-1P**	0.6	68
6^c^	**Py-1P**/**L1–Ir**	19.5	71
7^d^	—	2.0	58

a Reaction conditions: 10 mL formate solution (1.0 M) containing 6.35 μmol catalyst was irradiated with 460 nm LED light for 6 h.

b Without light at 45 °C (the photothermal effect raised the reaction temperature to *ca.* 42 °C).

c Physical mixture of **Py-1P** COF and **L1–Ir** with the same Ir and total mass loading as **Py-1P–Ir** COF.

d Reaction filtrate of entry 3.

**Fig. 6:**
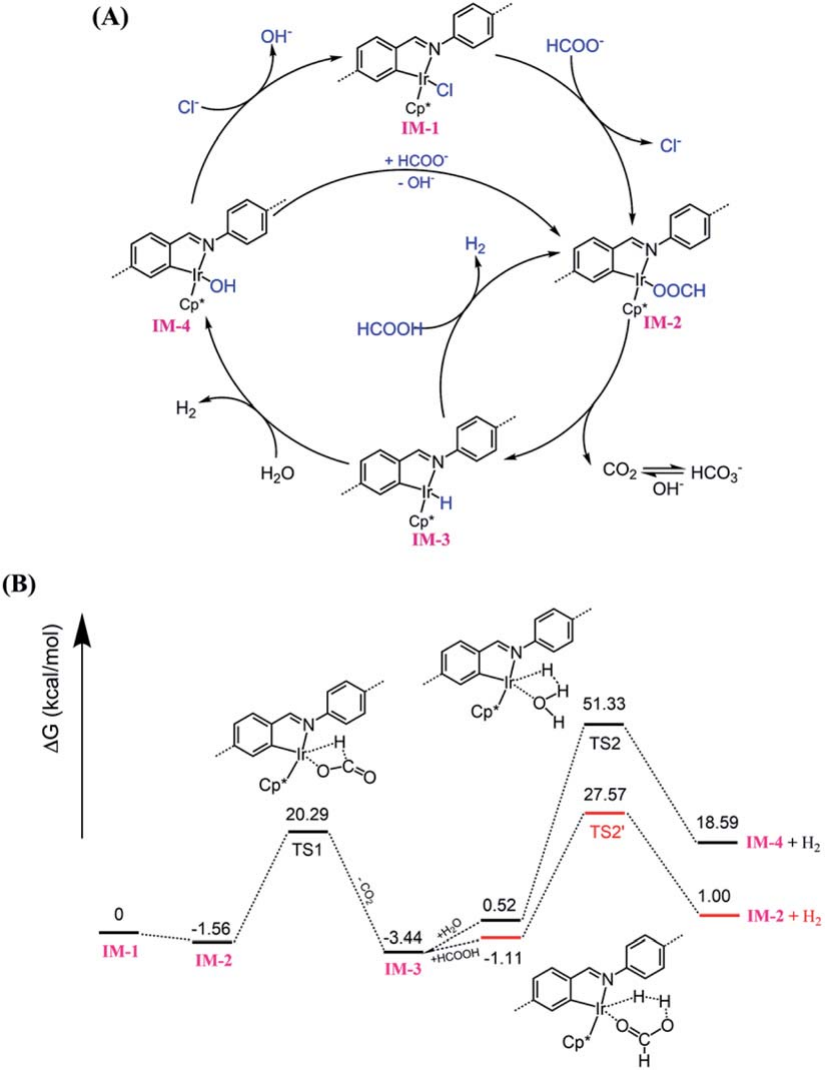
(A) Proposed mechanism for **Py-1P–Ir** COF catalyzed HER from formate; (B) calculated Gibbs free energy profile.

Based on literature reports,^52,53^ a proposed mechanism is shown in Fig. 6A. The catalytic cycle is initiated by Cl ligand replacement of **IM-1** with formate to give HCOO^−^ coordinated intermediate **IM-2**. CO_2_ extrusion from **IM-2** affords the key iridium hydride species **IM-3**. Then two pathways account for hydrogen production depending on the reaction conditions (pH). Under neutral or basic conditions, **IM-3** reacts with one molecular H_2_O to generate H_2_ and OH^−^ bonded Ir intermediate **IM-4**, which could be reconverted to **IM-1** or **IM-2***via* ligand exchange to close the catalytic cycle. While under acidic conditions, **IM-3** reacts with HCOOH to release H_2_ and regenerate **IM-2**. The involvement of iridium hydride intermediate was supported by NMR studies on **L1–Ir** in solution. Mixing **L1–Ir** and excess HCOONa in CD_3_CN/D_2_O (v/v, 4/1) generated an iridium hydride complex showing a hydride signal at −12.45 ppm in the ^1^H NMR spectrum (Fig. S32†).^52,73^ To further support the proposed mechanism, we carried out DFT calculations. The computed Gibbs free energy profile of the prosed reaction pathway is shown in Fig. 6B. **IM-2** is slightly lower (−1.56 kcal mol^−1^) in energy than **IM-1**, suggesting the first step ligand exchange is feasible. The transformation of **IM-2** to **IM-3***via* CO_2_ extrusion is thermodynamically favored (Δ*G* = 1.88 kcal mol^−1^) with a small energy barrier of 21.85 kcal mol^−1^ (**TS1**). This is consistent with our experimental findings that the iridium hydride formed immediately at room temperature. The reaction of **IM-3** with H_2_O to give **IM-4** and H_2_ is an energy demanding process (Δ*G* = 18.07 kcal mol^−1^) with an energy barrier of 50.81 kcal mol^−1^ (**TS2**). In contrast, the reaction of **IM-3** with HCOOH to give **IM-2** and H_2_ is energetically less demanding (Δ*G* = 2.11 kcal mol^−1^) and the activation energy is also significantly reduced to 28.68 kcal mol^−1^ (**TS2′**). These results match well with the observed higher reactivity of the catalyst in lower pH conditions.

## Conclusions

In summary, we identified a new metal binding mode of imine COFs where cyclometalation with iridium readily occurred. The iridium functionalized COF exhibited much enhanced catalytic efficiency in HER from an aqueous formate solution than the parent COF and the molecular counterpart in both thermal- and photo-triggered reactions. The easily available imine-based COFs, as well as various transition metals capable of forming metallacycles will largely advance surface organometallic chemistry in COFs beyond catalysis.

## Author contributions

J. Hu and H. Beyzavi conceived the work. J. Hu performed all experimental studies and data analysis. H. Mehrabi and R. H. Coridan performed GC analysis. Y.-S. Meng performed TGA and N_2_-sorption tests. M. Taylor was involved in linkers synthesis. J.-H. Zhan performed the DFT calculations. Q. Yan and M. Benamara performed XPS and TEM studies. J. Hu and H. Beyzavi wrote the manuscript with valuable inputs from all the authors. All authors reviewed and agreed with the content of the paper. H. Beyzavi led the project.

## Conflicts of interest

There are no conflicts to declare.

## Supplementary Material

SC-012-D1SC01692J-s001

SC-012-D1SC01692J-s002
