# Supplementary material for: Probe metal binding mode of imine covalent organic frameworks: cycloiridation for (photo)catalytic hydrogen evolution from formate
Source: Chem Sci. 2021 May 11;12(22):7930–6. doi: 10.1039/d1sc01692j (PMC8188469; doi:10.1039/d1sc01692j)
Supplement: SC-012-D1SC01692J-s001 [file SC-012-D1SC01692J-s001.pdf]

Electronic Supplementary Material (ESI) for Chemical Science.
This journal is © The Royal Society of Chemistry 2021

Cartesian Coordinates of All the Structures

IM-1

C	-22.04468700	-1.51762400	0.49314500
C	-21.18584600	0.80701600	0.54149400
H	-23.06039700	-1.13245100	0.47631400
C	-20.96225500	-0.57738200	0.52522600
C	-20.47460900	-3.37695500	0.51218300
C	-21.81121200	-2.85770400	0.48647800
C	-20.21712100	-4.75548500	0.51538100
H	-22.63685200	-3.56348300	0.46413000
C	-1.63752300	14.17854700	-0.56857600
C	-0.67753300	16.42494900	-0.59023600
C	-2.16752600	18.40896100	-0.54326600
H	0.18775100	17.08144200	-0.61955100
H	-13.17056500	3.94130700	-0.06598600
C	-18.52740600	-0.13587700	0.57580600
H	4.64577400	11.67928600	0.16150500
C	-4.42784100	16.73250700	-0.58569100
C	-5.40776000	14.50369000	-0.65377100
C	-3.92996300	12.48525000	-0.59829600
C	-5.06379200	11.52965600	-0.57222800
C	-5.11459300	10.47026400	-1.49419900
C	-6.12900300	9.52329500	-1.44465500
C	-7.15026200	9.62012700	-0.48620700
C	-7.09610700	10.66546100	0.45307100
C	-6.06654700	11.59899400	0.40938200
H	-6.27863400	13.86393100	-0.72523700
H	-4.34596200	10.39838900	-2.25752200
H	-6.16482900	8.70315400	-2.15397300
H	-7.83532200	10.71323400	1.24652500
H	-6.01653100	12.37758300	1.16384200
C	-12.44919900	5.99229800	0.06199700
C	-11.13480200	5.61072000	-0.26209400
C	-10.12126300	6.55337400	-0.34017200
C	-10.40029500	7.90841200	-0.09392200
C	-11.71426100	8.29013200	0.23147300
C	-12.72859700	7.34730700	0.30777100
H	-10.91778800	4.56268800	-0.45192300
H	-9.10412500	6.27286300	-0.58961600
H	-11.93112000	9.33794300	0.42267400
H	-13.74582800	7.62841600	0.55646100
C	-13.49539000	4.97254200	0.13531800
N	-14.71133100	5.25382200	0.42971000
C	15.00051600	3.57100300	0.64568500

C	15.84639400	5.92288800	0.45965300
C	14.49102200	6.52144700	0.49133000
C	14.19315000	7.55968000	1.39076100
C	12.95489800	8.18805100	1.37725100
C	11.95539800	7.78068300	0.47924200
C	12.24992200	6.75185300	-0.43380900
C	13.49701000	6.13784400	-0.42532600
H	13.99773100	3.94151700	0.82134500
H	14.94750700	7.86989900	2.10721900
H	12.72815700	8.99384300	2.06747800
H	11.51691600	6.47179600	-1.18381300
H	13.72295800	5.37256700	-1.16094600
C	4.60707700	10.60329300	-0.06284600
N	3.51440400	10.01934100	-0.39304000
C	-4.10948400	13.89043300	-0.59443100
C	-5.55975900	15.85584500	-0.64103200
C	-4.56727600	18.12924500	-0.56822000
H	-6.55204300	16.29582300	-0.68960200
H	9.60566300	6.79004800	0.00847900
C	16.06266100	4.52110300	0.47052900
H	-9.65836000	9.95447500	0.05229100
C	-1.98160400	17.01764300	-0.56024800
C	-0.51417800	15.07412700	-0.60274500
C	-1.48872400	12.76967800	-0.57262900
C	-0.16549800	12.10171300	-0.52202400
C	0.14975700	11.08870100	-1.44356700
C	1.35830500	10.40770900	-1.37689100
C	2.31124600	10.73840600	-0.40052500
C	1.99363900	11.73351600	0.54118000
C	0.77378200	12.39798900	0.47980900
H	0.48173500	14.65196000	-0.65461500
H	-0.56598400	10.84264100	-2.22192600
H	1.59998300	9.62480100	-2.08801300
H	2.68675500	11.94974400	1.34808700
H	0.52804700	13.13842200	1.23434700
C	8.35974100	8.57277900	0.16383300
C	7.18996200	7.86406000	-0.16418100
C	5.96555600	8.51113300	-0.23790300
C	5.88225400	9.89052000	0.01703500
C	7.05122800	10.59816500	0.35008600
C	8.27564200	9.95166300	0.42178900
H	7.25353000	6.79742600	-0.36363000
H	5.05712300	7.97720200	-0.49341100
H	6.98782200	11.66501600	0.54838700

H	9.18495100	10.48594400	0.67310100
C	9.64117700	7.86843900	0.22118400
N	10.73599700	8.47007700	0.50966800
C	-17.20153900	-0.67431300	0.69251600
C	-18.79127000	1.25772200	0.55242200
C	-17.70646200	2.26678500	0.51728100
C	-17.70863200	3.34935900	1.41342800
C	-16.72401100	4.32780900	1.35985800
C	-15.68178800	4.24217800	0.42321700
C	-15.68870900	3.17796900	-0.49648300
C	-16.68643700	2.21163100	-0.44779700
H	-16.37217400	0.01176700	0.81408200
H	-18.49006600	3.41135800	2.16456000
H	-16.72562300	5.16216400	2.05335100
H	-14.93527800	3.13737900	-1.27678700
H	-16.69666700	1.41342300	-1.18306500
C	-9.34822700	8.92286300	-0.16934300
N	-8.14179000	8.63013800	-0.48942600
C	-3.44772700	18.95717200	-0.54352500
C	-2.95544900	14.73689200	-0.57460200
H	-3.57355800	20.03557500	-0.53034600
C	-2.63213200	11.96712500	-0.60364700
C	-3.12109100	16.15743800	-0.57087300
H	-2.50685500	10.88977100	-0.56887900
C	-19.61793100	-1.06005500	0.54415200
C	-20.12230200	1.69881000	0.55276300
H	-20.31554700	2.76677400	0.52959000
C	2.33199900	-15.07934400	-0.79156700
C	3.41942300	-12.90209500	-0.69411900
C	2.03784000	-10.81949400	-0.56904300
C	3.22523300	-9.93751700	-0.45029600
C	3.41892300	-8.87617400	-1.35164300
C	4.50728500	-8.01920500	-1.23733000
C	5.43826100	-8.22089600	-0.21121400
C	5.25614400	-9.26226100	0.70581500
C	4.15645300	-10.10852800	0.58519300
H	4.32228200	-12.30406800	-0.69835800
H	2.71035700	-8.73835300	-2.16256900
H	4.67249400	-7.20541900	-1.93557700
H	5.95888200	-9.38251700	1.52448200
H	4.00402400	-10.89938000	1.31235400
C	10.63678400	-4.73949000	0.25565900
C	9.26877700	-4.44814500	0.15034800
C	8.30393000	-5.46065000	0.11897200

C	8.78705400	-6.79923300	0.13498000
C	10.15926700	-7.10295400	0.23077400
C	11.08164200	-6.07896800	0.30677100
H	8.96351600	-3.40832000	0.07708200
H	10.48492700	-8.14021900	0.23682300
H	12.14528100	-6.26881100	0.38782500
C	11.60018400	-3.63810500	0.30336700
N	12.85451700	-3.83946600	0.47783600
C	-16.96776900	-2.01556100	0.68905700
C	-17.81044600	-4.37067000	0.53405000
C	-16.45033800	-4.95892200	0.50330000
C	-16.10614000	-5.99674400	1.38667900
C	-14.85775900	-6.60235200	1.33169400
C	-13.89290400	-6.16945600	0.40809800
C	-14.23550200	-5.14564900	-0.49333200
C	-15.49411600	-4.55691300	-0.44512200
H	-15.95515700	-2.38107800	0.80877500
H	-16.83080400	-6.32209500	2.12665500
H	-14.59471500	-7.40567800	2.01180600
H	-13.52950200	-4.84845200	-1.26248400
H	-15.75682800	-3.79364100	-1.17051200
C	-6.47171800	-8.77569200	-0.18582100
N	-5.37758700	-8.16047600	-0.44909000
C	-0.33046000	-12.39382500	-0.70463200
C	-1.39652600	-14.58456200	-0.88999100
C	-0.00636200	-16.64036600	-0.91876800
H	-2.29119900	-15.19376400	-0.98460900
H	11.18244100	-2.62611200	0.19482700
C	16.54180600	1.68372500	0.43665700
H	-6.49704400	-9.86198100	-0.01607600
C	-0.12443500	-15.24295600	-0.85751600
C	-1.49425700	-13.22900300	-0.82505400
C	-0.41151400	-10.98108300	-0.62775300
C	-1.70932200	-10.26557900	-0.56652700
C	-1.98543100	-9.20729500	-1.44823600
C	-3.18777700	-8.51321200	-1.38250100
C	-4.17045400	-8.87490800	-0.44752300
C	-3.88474400	-9.90632200	0.46506400
C	-2.67323600	-10.58463800	0.40439600
H	-2.46669800	-12.75586600	-0.87954900
H	-1.24904000	-8.94110600	-2.20044600
H	-3.40310300	-7.70101900	-2.06883800
H	-4.59998800	-10.14559400	1.24562000
H	-2.45624700	-11.36164100	1.13048400

C	-10.27936900	-6.85204700	0.04701400
C	-9.12531000	-6.10055800	-0.23904200
C	-7.88177700	-6.71078700	-0.31583400
C	-7.76446600	-8.09570500	-0.10967700
C	-8.91897500	-8.84686700	0.17576000
C	-10.16125500	-8.23692400	0.25391800
H	-9.21527800	-5.02931100	-0.39976000
H	-6.98484200	-6.14238500	-0.53537600
H	-8.82908600	-9.91834800	0.33483700
H	-11.05865900	-8.80430500	0.47333500
C	-11.58078200	-6.18616200	0.12355500
N	-12.65522600	-6.82604400	0.40614300
C	15.22772400	2.22836800	0.63192100
C	16.79781400	0.28900900	0.39190200
C	15.72019900	-0.72805400	0.43257200
C	15.82370700	-1.82718100	1.30358400
C	14.87322600	-2.83848200	1.30095100
C	13.76074300	-2.77298800	0.44630700
C	13.65224000	-1.68150100	-0.43393700
C	14.62005600	-0.68212200	-0.44059700
H	14.40341900	1.54539800	0.79769300
H	16.66422500	-1.88000600	1.98869700
H	14.96090300	-3.69110300	1.96617400
H	12.83637600	-1.64952800	-1.14932800
H	14.54538200	0.13006600	-1.15664300
C	7.75828100	-7.78405700	0.00048600
N	6.53672000	-7.33792200	-0.08848700
C	2.15023100	-12.22951600	-0.64104900
C	3.50585500	-14.25845700	-0.75784300
C	2.40362800	-16.47984400	-0.85390500
H	4.47704400	-14.74364000	-0.80089100
H	-11.57869200	-5.10225400	-0.06255900
C	-18.03322000	-2.97009600	0.56663300
H	7.95881900	-8.85313800	-0.06107300
C	0.76926400	-10.23444300	-0.58294000
C	1.05468400	-14.44183200	-0.78247900
H	0.69598400	-9.15499600	-0.49394300
C	1.24535900	-17.25081900	-0.91336500
C	0.95757100	-13.01705000	-0.70665900
H	1.31876400	-18.33301700	-0.96156200
C	-18.91486800	-5.23513000	0.52416700
H	-18.73630200	-6.30536000	0.49178700
C	-19.37232800	-2.46883600	0.53843100
C	18.49927800	4.91715900	0.28337900

C	19.82948200	4.39291800	0.17421000
C	18.25019700	6.29675700	0.31519100
H	20.65578900	5.09541100	0.11068400
C	17.39544300	4.01372300	0.36411900
C	16.95306400	6.78238100	0.39908200
H	16.77819200	7.85360500	0.38460600
C	20.05558900	3.05190000	0.15221600
C	19.19004700	0.73176800	0.22822800
H	21.06626700	2.66173200	0.07063800
C	18.97209300	2.11676100	0.23969500
C	18.12559300	-0.15505100	0.30063100
H	18.31304900	-1.22318200	0.25597900
C	17.63329800	2.60376600	0.34427000
H	-0.90874500	-17.24264400	-0.97464800
H	-1.29500000	19.05612300	-0.53378200
H	-5.56525700	18.55837300	-0.57820100
H	19.08683800	6.98706400	0.25436100
H	20.20619600	0.35651200	0.14535300
H	3.37954300	-16.95702300	-0.85925800
H	-21.05233700	-5.44984000	0.49410200
H	-22.20674600	1.17815200	0.52743100
Ir	6.30042000	-5.26634800	-0.00610600
C	5.24568700	-4.91467700	1.87511000
C	6.03498600	-3.74739300	1.54898900
C	5.54263900	-3.22723600	0.30108400
C	4.33240400	-3.98314800	-0.04561300
C	4.14339900	-4.99305800	0.91370700
C	3.03558200	-5.99590700	0.97998700
C	3.51688800	-3.72165900	-1.26977000
C	6.01425100	-2.00920300	-0.42876900
C	7.12380400	-3.17212400	2.39692900
C	5.36068400	-5.74528600	3.11552800
H	3.40150800	-6.97602000	1.29419000
H	2.54871800	-6.12471300	0.01244500
H	2.27638200	-5.67624200	1.70446100
H	3.07786200	-2.71811700	-1.23256100
H	2.70543900	-4.44455700	-1.37253600
H	4.14829100	-3.78852400	-2.16143900
H	6.01133100	-2.18755600	-1.50679000
H	7.03484600	-1.74715000	-0.14039300
H	5.37221300	-1.14468300	-0.21784800
H	6.68850800	-2.56285100	3.19789500
H	7.79009900	-2.53276900	1.81647900
H	7.73104900	-3.95589900	2.85422100

H	6.38375700	-5.73902300	3.49774000
H	5.08415200	-6.78386500	2.91574400
H	4.69994200	-5.36877300	3.90651600
Cl	6.52462800	-5.22235700	-2.43381600

IM-2

C	-22.11246400	-1.44428800	0.56462700
C	-21.23746300	0.87413700	0.62296400
H	-23.12561400	-1.05214900	0.55657400
C	-21.02348400	-0.51163900	0.59658500
C	-20.55500300	-3.31431700	0.56095800
C	-21.88816400	-2.78584700	0.54706300
C	-20.30685700	-4.69454600	0.55200300
H	-22.71871800	-3.48583500	0.52461900
C	-1.62218300	14.13303700	-0.62601500
C	-0.65593100	16.37634400	-0.67536800
C	-2.14013200	18.36502800	-0.64460800
H	0.21110500	17.02998600	-0.71485800
H	-13.20440400	3.95469600	-0.01680300
C	-18.58547300	-0.08700900	0.63562900
H	4.65653800	11.63192500	0.10467900
C	-4.40530700	16.69469100	-0.66097100
C	-5.39172000	14.46807300	-0.70159000
C	-3.91938000	12.44613100	-0.62995800
C	-5.05625200	11.49457800	-0.59058100
C	-5.11483600	10.42738100	-1.50298000
C	-6.13342400	9.48554300	-1.44175400
C	-7.15099400	9.59538400	-0.48079800
C	-7.08849400	10.64817200	0.44962200
C	-6.05497200	11.57665500	0.39418100
H	-6.26465500	13.83008300	-0.76301200
H	-4.34922300	10.34548000	-2.26832400
H	-6.17560400	8.65957900	-2.14393000
H	-7.82455000	10.70614100	1.24532700
H	-5.99878300	12.36160800	1.14158200
C	-12.46884300	6.00099000	0.10596000
C	-11.15832100	5.61029500	-0.22294600
C	-10.13928700	6.54637600	-0.30836800
C	-10.40882200	7.90388300	-0.06513500
C	-11.71885800	8.29467300	0.26533700
C	-12.73863100	7.35838600	0.34927400
H	-10.94873700	4.56040700	-0.41087300
H	-9.12508600	6.25884800	-0.56174700
H	-11.92836700	9.34439800	0.45421300

H	-13.75294800	7.64657700	0.60174000
C	-13.52128600	4.98818200	0.18568600
N	-14.73389200	5.27750600	0.48602800
C	14.99594600	3.52014700	0.66784500
C	15.85102800	5.86590800	0.44973800
C	14.49776400	6.46957200	0.47377200
C	14.20368600	7.52101500	1.35898400
C	12.96667200	8.15169800	1.33882700
C	11.96466000	7.73363400	0.44856500
C	12.25567500	6.69202100	-0.45099300
C	13.50156100	6.07574600	-0.43617300
H	13.99477600	3.89679300	0.83945500
H	14.95977200	7.83936600	2.07004100
H	12.74264600	8.96726300	2.01838600
H	11.52066100	6.40275900	-1.19550100
H	13.72449300	5.29971700	-1.16135400
C	4.61611800	10.55308600	-0.10537800
N	3.52139800	9.96601100	-0.42310200
C	-4.09496400	13.85178100	-0.64030300
C	-5.53987600	15.82071300	-0.70283100
C	-4.54074000	18.09193100	-0.65810600
H	-6.53109100	16.26297200	-0.75255800
H	9.61310300	6.73834200	-0.00079700
C	16.06179800	4.46353000	0.47954400
H	-9.65472200	9.94630900	0.06767400
C	-1.95819900	16.97308000	-0.64719300
C	-0.49644400	15.02500600	-0.67381400
C	-1.47729900	12.72376500	-0.61529600
C	-0.15557600	12.05303300	-0.56169700
C	0.15454300	11.02943700	-1.47323600
C	1.36213600	10.34701000	-1.40349500
C	2.31920000	10.68660800	-0.43422200
C	2.00657600	11.69226100	0.49790300
C	0.78768500	12.35823900	0.43367100
H	0.49807600	14.59945300	-0.72458400
H	-0.56435700	10.77630300	-2.24641300
H	1.59988900	9.55603500	-2.10697100
H	2.70300200	11.91588100	1.29992800
H	0.54597400	13.10726100	1.18100500
C	8.36860900	8.52384300	0.13358500
C	7.19723900	7.81119500	-0.17995500
C	5.97281900	8.45785000	-0.25769800
C	5.89117500	9.84065500	-0.02144200
C	7.06182800	10.55228900	0.29697100

C	8.28621700	9.90623200	0.37266000
H	7.25945900	6.74186200	-0.36473400
H	5.06318500	7.92073800	-0.50201800
H	6.99966000	11.62180800	0.48081500
H	9.19678500	10.44341900	0.61298300
C	9.64991800	7.81959700	0.19629900
N	10.74601000	8.42470400	0.47260100
C	-17.26277200	-0.63519600	0.74219500
C	-18.83982400	1.30849200	0.62161600
C	-17.74826600	2.31012600	0.58386500
C	-17.73768300	3.38994300	1.48331400
C	-16.74689200	4.36198800	1.42671100
C	-15.71092500	4.27221500	0.48351900
C	-15.73038200	3.21071300	-0.43910200
C	-16.73433900	2.25102100	-0.38739200
H	-16.42819000	0.04440600	0.86445200
H	-18.51413700	3.45493700	2.23934400
H	-16.73886100	5.19427900	2.12263800
H	-14.98185800	3.16751100	-1.22397600
H	-16.75437200	1.45521600	-1.12505000
C	-9.35122400	8.91185100	-0.14979700
N	-8.14781900	8.61067100	-0.47346400
C	-3.41877400	18.91686600	-0.64630100
C	-2.93850900	14.69509900	-0.63352800
H	-3.54151000	19.99570800	-0.64434000
C	-2.62308500	11.92417100	-0.63407700
C	-3.10013900	16.11607800	-0.64453000
H	-2.50075300	10.84687700	-0.58840900
C	-19.68241300	-1.00355500	0.60441200
C	-20.16780200	1.75860500	0.63282100
H	-20.35392200	2.82796500	0.61682300
C	2.26209700	-15.01015900	-0.97646600
C	3.33848100	-12.83362600	-0.78533500
C	1.94605600	-10.76236300	-0.60071200
C	3.12715400	-9.88049200	-0.43100400
C	3.32374900	-8.77928800	-1.28267500
C	4.40600600	-7.92279400	-1.11795200
C	5.32843200	-8.16393400	-0.09255100
C	5.14125200	-9.24480400	0.77699800
C	4.04803600	-10.09033300	0.60698400
H	4.23878000	-12.23245300	-0.75604300
H	2.62238000	-8.60922300	-2.09365100
H	4.57794300	-7.07961700	-1.77803100
H	5.83430000	-9.39659500	1.59851500

H	3.89140500	-10.91180500	1.29836700
C	10.57008300	-4.73705600	0.38375700
C	9.20746400	-4.42421900	0.26202000
C	8.23048800	-5.42396900	0.23735100
C	8.68897600	-6.76855600	0.27948600
C	10.05535100	-7.09406900	0.39164300
C	10.99285200	-6.08249100	0.45887700
H	8.91545800	-3.38416900	0.15907000
H	10.36519600	-8.13597200	0.41813100
H	12.05280600	-6.28788000	0.55116200
C	11.54967200	-3.64941500	0.41650800
N	12.80116700	-3.86612300	0.59464900
C	-17.03816900	-1.97793100	0.72850000
C	-17.89750900	-4.32619500	0.55930300
C	-16.54153800	-4.92302100	0.51567300
C	-16.19972500	-5.97249700	1.38620200
C	-14.95503700	-6.58455800	1.31971100
C	-13.99120500	-6.14631900	0.39760900
C	-14.33163600	-5.11120500	-0.49155400
C	-15.58695900	-4.51647900	-0.43245600
H	-16.02750600	-2.35099200	0.84103700
H	-16.92313500	-6.30163000	2.12573100
H	-14.69391200	-7.39654000	1.99021800
H	-13.62656100	-4.80954500	-1.25982400
H	-15.84823300	-3.74418300	-1.14873000
C	-6.57351400	-8.75378700	-0.23928000
N	-5.47755500	-8.13191600	-0.47818800
C	-0.41311700	-12.34026100	-0.82594000
C	-1.46710400	-14.52694700	-1.10384000
C	-0.06752200	-16.57469500	-1.19008300
H	-2.35781500	-15.13594400	-1.23136300
H	11.14567200	-2.63423600	0.29146900
C	16.52936300	1.62413000	0.48193700
H	-6.60071400	-9.84432500	-0.09980000
C	-0.19254800	-15.18092100	-1.07954000
C	-1.57166100	-13.17504700	-0.99096000
C	-0.50154600	-10.93162400	-0.69921200
C	-1.80318800	-10.22385600	-0.62912600
C	-2.07334600	-9.13715500	-1.47736700
C	-3.27858700	-8.44930300	-1.40094400
C	-4.26972400	-8.84484900	-0.48885300
C	-3.99051700	-9.90628300	0.39080100
C	-2.77650100	-10.57905100	0.31960700
H	-2.54542700	-12.70400500	-1.03991300

H	-1.32954500	-8.84299300	-2.21165500
H	-3.48926700	-7.61449800	-2.06108400
H	-4.71274200	-10.17391400	1.15554300
H	-2.56468900	-11.37986200	1.02094900
C	-10.37966200	-6.83141800	0.02501100
C	-9.22364400	-6.07510600	-0.23956000
C	-7.98068800	-6.68507900	-0.32601100
C	-7.86602200	-8.07468000	-0.15231400
C	-9.02258600	-8.83080200	0.11081600
C	-10.26427200	-8.22096400	0.19947700
H	-9.31162100	-5.00025200	-0.37531600
H	-7.08212400	-6.11296000	-0.52852300
H	-8.93477800	-9.90591000	0.24459200
H	-11.16317200	-8.79205300	0.40258700
C	-11.67999000	-6.16494100	0.11359500
N	-12.75596400	-6.80748100	0.38402800
C	15.21764300	2.17656400	0.67113100
C	16.77940200	0.22783900	0.45392600
C	15.69638100	-0.78286100	0.50563300
C	15.79181700	-1.87210000	1.38978300
C	14.83252600	-2.87518700	1.40059700
C	13.71957600	-2.81032000	0.54660800
C	13.62034600	-1.72992800	-0.34838700
C	14.59659900	-0.73921500	-0.36819400
H	14.39056300	1.49932600	0.84612800
H	16.63210600	-1.92312200	2.07534200
H	14.91295400	-3.71962400	2.07710500
H	12.80368600	-1.69933600	-1.06287700
H	14.52797600	0.06474400	-1.09403600
C	7.64450800	-7.74075300	0.15273100
N	6.42662100	-7.28726600	0.06022400
C	2.06582200	-12.16836000	-0.72363900
C	3.43173800	-14.18638200	-0.89783800
C	2.34072100	-16.40715700	-1.08887800
H	4.40554500	-14.66578500	-0.94628700
H	-11.67589800	-5.07769700	-0.05188700
C	-18.11061800	-2.92440700	0.60437500
H	7.83633900	-8.81218000	0.09552700
C	0.67501000	-10.18232600	-0.61101700
C	0.98200400	-14.37841300	-0.96045800
H	0.59523100	-9.10723700	-0.48322600
C	1.18670300	-17.18001200	-1.19103900
C	0.87765200	-12.95774500	-0.83390800
H	1.26545100	-18.25943900	-1.27754800

C	-19.00787700	-5.18303100	0.54907200
H	-18.83686600	-6.25415600	0.50693600
C	-19.44643100	-2.41390400	0.58749200
C	18.49994200	4.84728400	0.28695200
C	19.82799400	4.31615500	0.18484700
C	18.25633200	6.22818400	0.30030600
H	20.65719700	5.01435900	0.11220900
C	17.39250700	3.94944000	0.37963900
C	16.96111500	6.72007600	0.37755700
H	16.79050900	7.79170500	0.34865000
C	20.04864900	2.97402600	0.18043400
C	19.17349900	0.65849200	0.28597600
H	21.05777000	2.57876200	0.10408700
C	18.96136900	2.04446000	0.27980800
C	18.10527800	-0.22295000	0.36885000
H	18.28818000	-1.29236200	0.33746700
C	17.62462600	2.53836900	0.37802700
H	-0.96646400	-17.17811700	-1.27910900
H	-1.26574900	19.00975100	-0.64518800
H	-5.53753900	18.52377800	-0.66921200
H	19.09572500	6.91428000	0.23026600
H	20.18812600	0.27801700	0.20823600
H	3.31871400	-16.87998000	-1.09908300
H	-21.14688700	-5.38305700	0.53029200
H	-22.25586700	1.25229200	0.61742300
Ir	6.22359700	-5.19951500	0.10465100
C	4.89633800	-4.96443100	1.84366100
C	5.93018200	-3.96100800	1.88469300
C	5.77301600	-3.12228900	0.71346900
C	4.58971100	-3.58430300	0.01231800
C	4.02855800	-4.68936800	0.70700300
C	2.76181300	-5.41636500	0.38303300
C	4.07839200	-2.97536800	-1.25403800
C	6.53514600	-1.87468400	0.39448200
C	6.94404300	-3.77064800	2.96851500
C	4.65142700	-6.03059800	2.86587900
H	2.83016300	-6.47626200	0.63671700
H	2.52568300	-5.35044800	-0.68155900
H	1.91513500	-4.99323300	0.93867200
H	3.45231400	-2.10381800	-1.02645800
H	3.47408700	-3.68492000	-1.82332800
H	4.91119900	-2.65058200	-1.87920300
H	6.81590600	-1.86610400	-0.65981900
H	7.44848900	-1.81390500	0.99122000

H	5.93616800	-0.98339300	0.61970600
H	6.60165700	-3.01481500	3.68524700
H	7.90274200	-3.44370700	2.56083700
H	7.11948800	-4.70034600	3.51369600
H	5.56865800	-6.28107900	3.40290600
H	4.27592400	-6.94465300	2.39937500
H	3.90808400	-5.69763700	3.60017400
O	6.46664200	-5.45794700	-1.98108200
C	6.93066600	-4.48089000	-2.70043700
O	7.23772800	-3.34698700	-2.34774300
H	7.02682700	-4.78642900	-3.76283100

TS1

C	22.13007300	-1.44496900	-0.40957700
C	21.25754600	0.87507800	-0.42482800
H	23.14322600	-1.05413600	-0.37677900
C	21.04226200	-0.51068600	-0.43441400
C	20.57166700	-3.31300600	-0.47661800
C	21.90481200	-2.78632300	-0.42964000
C	20.32261400	-4.69274300	-0.50846600
H	22.73457300	-3.48740800	-0.41310600
C	1.62219400	14.13106500	0.50445200
C	0.65666000	16.37517700	0.50636200
C	2.14245600	18.36262000	0.47394800
H	-0.21049400	17.02966600	0.52193800
H	13.21491000	3.95182500	0.14103600
C	18.60525000	-0.08284100	-0.49911100
H	-4.64557400	11.62616700	-0.30386100
C	4.40594000	16.69174000	0.55150600
C	5.39006300	14.46544000	0.63880400
C	3.91789800	12.44319900	0.56857300
C	5.05476500	11.49091700	0.55816300
C	5.09809600	10.43288800	1.48208800
C	6.11716000	9.49016200	1.44705200
C	7.15048800	9.59032800	0.50198700
C	7.10369700	10.63385100	-0.43969000
C	6.06959500	11.56285400	-0.41078300
H	6.26144400	13.82805200	0.72381500
H	4.32013400	10.35887000	2.23569700
H	6.14735500	8.67136500	2.15819000
H	7.85293800	10.68361400	-1.22355600
H	6.02600800	12.34013100	-1.16698300
C	12.48079500	5.99873800	0.01736200
C	11.16511400	5.60672000	0.32330700

C	10.14396700	6.54195400	0.39196200
C	10.41650100	7.89981900	0.15420800
C	11.73194900	8.29205600	-0.15217300
C	12.75380900	7.35672500	-0.21899600
H	10.95302000	4.55640700	0.50598300
H	9.12565400	6.25324300	0.62687900
H	11.94381300	9.34209000	-0.33663800
H	13.77209200	7.64590500	-0.45370600
C	13.53487400	4.98661900	-0.04953100
N	14.75196700	5.27850400	-0.32839400
C	-14.99375600	3.52204800	-0.77936900
C	-15.84762900	5.87613500	-0.67696200
C	-14.49272700	6.47607600	-0.69924000
C	-14.17818700	7.49054600	-1.61988500
C	-12.94023900	8.11950800	-1.60007500
C	-11.95739100	7.73574900	-0.67373100
C	-12.26905200	6.73158500	0.26080500
C	-13.51596700	6.11750000	0.24572700
H	-13.98919300	3.88966600	-0.95083700
H	-14.91900400	7.78144900	-2.35825100
H	-12.70071800	8.90683400	-2.30710700
H	-11.55000000	6.47165800	1.03127600
H	-13.75559100	5.37200000	0.99711700
C	-4.61181100	10.55293100	-0.06594500
N	-3.52469900	9.97117500	0.28593000
C	4.09415600	13.84885000	0.56361200
C	5.53903400	15.81791400	0.62453800
C	4.54230700	18.08875300	0.53202800
H	6.52954000	16.26036200	0.68542500
H	-9.61925900	6.75185500	-0.14296500
C	-16.06073000	4.47404600	-0.65030400
H	9.66276500	9.94089100	0.00229600
C	1.95961500	16.97091400	0.49235500
C	0.49634500	15.02401600	0.52068500
C	1.47662100	12.72187600	0.51114100
C	0.15508800	12.05165200	0.44651700
C	-0.17244700	11.04515000	1.37078900
C	-1.38057400	10.36443100	1.29324800
C	-2.32061400	10.68864800	0.30230100
C	-1.98975300	11.67625600	-0.64278700
C	-0.77059100	12.34079200	-0.56996100
H	-0.49916700	14.59957600	0.56016900
H	0.53289100	10.80446100	2.16025700
H	-1.63223700	9.58705500	2.00697100

H	-2.67196400	11.88706500	-1.46034700
H	-0.51467700	13.07608500	-1.32616400
C	-8.36588200	8.52766900	-0.31573500
C	-7.20324000	7.82206200	0.04299000
C	-5.97829200	8.46723100	0.12387400
C	-5.88730200	9.84172400	-0.15387000
C	-7.04913500	10.54624400	-0.51724400
C	-8.27401200	9.90150300	-0.59686800
H	-7.27293700	6.75942500	0.26086700
H	-5.07541600	7.93571100	0.40301700
H	-6.97986600	11.60936300	-0.73288800
H	-9.17786200	10.43351000	-0.87163300
C	-9.64816700	7.82540800	-0.37973500
N	-10.73650800	8.42285300	-0.69982300
C	17.28360100	-0.62711700	-0.63501900
C	18.86045700	1.31173400	-0.45137300
C	17.76919400	2.31385200	-0.41423800
C	17.77367300	3.40648300	-1.29804800
C	16.78164300	4.37737400	-1.24441100
C	15.72977600	4.27399300	-0.32043000
C	15.73442600	3.19995400	0.58779400
C	16.73942600	2.24107800	0.53924800
H	16.45094800	0.05594300	-0.75085200
H	18.56271300	3.48216800	-2.03991200
H	16.78476200	5.21936800	-1.92859600
H	14.97282700	3.14534500	1.35927000
H	16.74720600	1.43468000	1.26557900
C	9.35645600	8.90675600	0.21736900
N	8.14734500	8.60570400	0.51920500
C	3.42123500	18.91391100	0.48993400
C	2.93857100	14.69256300	0.52628000
H	3.54467300	19.99257300	0.47545000
C	2.62127800	11.92196400	0.55911900
C	3.10088700	16.11349300	0.52074600
H	2.49880000	10.84421300	0.52610900
C	19.70113000	-1.00089900	-0.47360000
C	20.18872100	1.76061800	-0.43166200
H	20.37522300	2.82920800	-0.38975200
C	-2.28124100	-15.02896900	0.30972400
C	-3.34236300	-12.83931000	0.41647300
C	-1.93616400	-10.76916100	0.42582300
C	-3.11297900	-9.86549800	0.41136600
C	-3.26336100	-8.87682000	1.39826300
C	-4.34179200	-7.99922300	1.38430200

C	-5.30721400	-8.10005600	0.37701700
C	-5.16564800	-9.06987000	-0.62331300
C	-4.07799900	-9.93898000	-0.60474200
H	-4.23745700	-12.23451100	0.49309200
H	-2.53035800	-8.81302000	2.19627600
H	-4.45776200	-7.24467800	2.15387200
H	-5.89677500	-9.11564000	-1.42433900
H	-3.96307300	-10.67293300	-1.39555800
C	-10.57300900	-4.69517000	-0.06611300
C	-9.20849400	-4.37121400	-0.01277900
C	-8.23014100	-5.36173000	0.07627500
C	-8.68072900	-6.70419100	0.16288900
C	-10.04613300	-7.04207900	0.12208700
C	-10.98975100	-6.04180200	-0.00759100
H	-8.92404900	-3.32345500	-0.00875300
H	-10.35130200	-8.08185900	0.20438900
H	-12.05091800	-6.25727900	-0.04733900
C	-11.55775500	-3.61682000	-0.16950900
N	-12.80804800	-3.84995600	-0.33170200
C	17.05802100	-1.96958600	-0.65617700
C	17.91414600	-4.32176200	-0.53887400
C	16.55680000	-4.91707800	-0.52879500
C	16.21988200	-5.93333300	-1.43936700
C	14.97257200	-6.54301500	-1.40556300
C	14.00251800	-6.13634900	-0.47556900
C	14.33888900	-5.13576900	0.45401900
C	15.59561400	-4.54214000	0.42571600
H	16.04850600	-2.33915100	-0.78910000
H	16.94891400	-6.23798400	-2.18385800
H	14.71433600	-7.32909000	-2.10732700
H	13.62948500	-4.86035100	1.22809400
H	15.85319300	-3.79632200	1.17085500
C	6.58136300	-8.76547900	0.02485200
N	5.49339600	-8.16451100	0.34116800
C	0.41355300	-12.37507700	0.37513600
C	1.45458000	-14.58544800	0.35749700
C	0.03872400	-16.62098400	0.25270700
H	2.34306900	-15.21032900	0.38154300
H	-11.15930500	-2.59313300	-0.11242900
C	-16.53343600	1.63773300	-0.53456600
H	6.60020400	-9.83961400	-0.21061900
C	0.17357900	-15.22465000	0.30198100
C	1.56869300	-13.23030900	0.40031100
C	0.51177000	-10.96173300	0.41029700

C	1.81568700	-10.25572100	0.37749000
C	2.11632800	-9.26720200	1.32962300
C	3.32135600	-8.57615500	1.29157700
C	4.28349600	-8.87308400	0.31336700
C	3.97450000	-9.83439000	-0.66559800
C	2.75934900	-10.50846300	-0.63204400
H	2.54816600	-12.77418000	0.47061800
H	1.39659800	-9.05361800	2.11404700
H	3.55487000	-7.81756700	2.03123200
H	4.67417300	-10.01995900	-1.47439100
H	2.52367300	-11.22828000	-1.40942400
C	10.38959900	-6.83625000	-0.14028700
C	9.23830100	-6.09917600	0.19086900
C	7.99501800	-6.71187800	0.24705200
C	7.87476100	-8.08454000	-0.02787600
C	9.02614200	-8.82107600	-0.36011700
C	10.26865200	-8.20892600	-0.41562500
H	9.33050500	-5.03733200	0.40407200
H	7.10043300	-6.15479300	0.50225200
H	8.93392700	-9.88300500	-0.57287500
H	11.16397700	-8.76520000	-0.66930200
C	11.69059900	-6.16727100	-0.19128000
N	12.76572900	-6.79461300	-0.49838400
C	-15.21792600	2.17990000	-0.72716600
C	-16.78633900	0.24441200	-0.44629400
C	-15.70439300	-0.76860900	-0.43282400
C	-15.78973700	-1.90074500	-1.26264500
C	-14.83176700	-2.90381900	-1.21113700
C	-13.72919300	-2.79560900	-0.34826400
C	-13.64004500	-1.67243300	0.49329500
C	-14.61620100	-0.68224100	0.45223900
H	-14.38936900	1.49471700	-0.85874100
H	-16.62124000	-1.98556200	-1.95550600
H	-14.90505700	-3.78106400	-1.84537700
H	-12.83303800	-1.60829500	1.21646900
H	-14.55679200	0.15581100	1.13922300
C	-7.62883800	-7.66208400	0.32220900
N	-6.40669200	-7.20661900	0.33927800
C	-2.06615800	-12.17880800	0.38598600
C	-3.44481300	-14.19533400	0.37206800
C	-2.36976200	-16.42899900	0.26025500
H	-4.42089100	-14.67162000	0.40029700
H	11.68710400	-5.09157700	0.03731600
C	18.12853400	-2.91959100	-0.54321200

H	-7.81332100	-8.73026300	0.42925100
C	-0.66020900	-10.20128800	0.45465400
C	-0.99634100	-14.40700200	0.32067700
H	-0.57460200	-9.11906900	0.45125500
C	-1.22061900	-17.21508900	0.22852900
C	-0.88234800	-12.98198000	0.35791300
H	-1.30699900	-18.29670400	0.19081900
C	19.02335400	-5.17992600	-0.53725800
H	18.85084000	-6.25161400	-0.52735800
C	19.46421000	-2.41099000	-0.49513500
C	-18.50103400	4.86864200	-0.51785900
C	-19.83165000	4.34407600	-0.41531900
C	-18.25450200	6.24735200	-0.58762200
H	-20.66068900	5.04571000	-0.38837600
C	-17.39390000	3.96630000	-0.55136100
C	-16.95713800	6.73396800	-0.66241500
H	-16.78506500	7.80566000	-0.67639100
C	-20.05479400	3.00373400	-0.35419900
C	-19.18214300	0.68489800	-0.33855300
H	-21.06577400	2.61339600	-0.27729900
C	-18.96768300	2.06947600	-0.39263900
C	-18.11431100	-0.20052700	-0.36245600
H	-18.29979400	-1.26704800	-0.28393600
C	-17.62859000	2.55694700	-0.49062000
H	0.93419300	-17.23581200	0.23770200
H	1.26861000	19.00765300	0.45087300
H	5.53904400	18.52030500	0.55428500
H	-19.09366300	6.93683400	-0.56272100
H	-20.19855700	0.30940600	-0.26013100
H	-3.35140200	-16.89415800	0.25123300
H	21.16186300	-5.38238300	-0.49379600
H	22.27598100	1.25200200	-0.39501800
Ir	-6.21579200	-5.11647100	0.22183700
C	-4.95465700	-4.80076000	-1.48392600
C	-5.91758100	-3.70627600	-1.38930800
C	-5.66724300	-3.01923300	-0.15399200
C	-4.48366800	-3.62773900	0.45381200
C	-4.01472600	-4.67474100	-0.37452600
C	-2.79219100	-5.51412900	-0.18199000
C	-3.95855400	-3.21199400	1.78790800
C	-6.36845500	-1.81256100	0.38387200
C	-6.92823100	-3.34830200	-2.42982200
C	-4.80421900	-5.74470400	-2.63377000
H	-2.94886200	-6.53802200	-0.52624000

H	-2.50109600	-5.56383400	0.86883900
H	-1.94895800	-5.09608800	-0.74581000
H	-3.60030300	-2.17682800	1.75090200
H	-3.13607200	-3.84964100	2.11682300
H	-4.77623100	-3.27228900	2.51528100
H	-6.59570200	-1.95720300	1.44143700
H	-7.30858600	-1.63645000	-0.14368900
H	-5.74819700	-0.91535100	0.26620700
H	-6.48008800	-2.67386200	-3.16921800
H	-7.79199700	-2.84612500	-1.99275200
H	-7.29095500	-4.23557100	-2.95220500
H	-5.75822800	-5.90025100	-3.14143000
H	-4.43917800	-6.71729500	-2.29521200
H	-4.08822900	-5.34910800	-3.36456900
O	-6.95124900	-4.04995300	2.63438100
C	-6.97546900	-5.30376600	2.81269000
O	-7.78541800	-6.06795800	3.32538200
H	-6.03345300	-5.82223800	2.35288800

IM3

C	-22.01068500	-1.23554200	0.41082100
C	-21.10461900	1.07129700	0.45223700
H	-23.01813200	-0.82973800	0.38464600
C	-20.90942200	-0.31746200	0.44501400
C	-20.47933500	-3.12657900	0.45204600
C	-21.80478700	-2.58015300	0.41391900
C	-20.25019700	-4.51005000	0.46602400
H	-22.64461800	-3.26894000	0.39019000
C	-1.29606700	14.06457500	-0.49990200
C	-0.30018100	16.29542000	-0.49960300
C	-1.75835200	18.30260000	-0.44026000
H	0.57559800	16.93817100	-0.52064600
H	-13.02076300	4.03882400	-0.10651500
C	-18.46638500	0.07427500	0.51031400
H	4.94472500	11.46919000	0.22231100
C	-4.04505000	16.66302100	-0.50385900
C	-5.06024500	14.45083700	-0.59374400
C	-3.61517800	12.40845900	-0.55013000
C	-4.76466300	11.47151200	-0.53470400
C	-4.83066100	10.42014300	-1.46486900
C	-5.86186300	9.49090000	-1.42633300
C	-6.88502500	9.59873500	-0.47110700
C	-6.81560800	10.63540700	0.47671100
C	-5.76944200	11.55072000	0.44408800

H	-5.94103800	13.82580800	-0.67331600
H	-4.06070300	10.34061400	-2.22610200
H	-5.90957500	8.67711000	-2.14224100
H	-7.55688400	10.69008600	1.26778800
H	-5.70844700	12.32240100	1.20480200
C	-12.25858000	6.07462900	0.02935000
C	-10.95006400	5.66736800	-0.28721400
C	-9.91705300	6.58947500	-0.35566100
C	-10.17010800	7.94911900	-0.10679900
C	-11.47822200	8.35648600	0.21102200
C	-12.51201700	7.43432200	0.27742300
H	-10.75307300	4.61568400	-0.47867800
H	-8.90416300	6.28901900	-0.59912800
H	-11.67496200	9.40787700	0.40431300
H	-13.52495400	7.73527000	0.52033800
C	-13.32588100	5.07629600	0.09356100
N	-14.53780000	5.38239200	0.37964600
C	15.17747900	3.21480900	0.64375500
C	16.06413600	5.55521200	0.50649600
C	14.71850100	6.17554700	0.53474000
C	14.42915900	7.20445600	1.44763900
C	13.20076600	7.85189700	1.43429300
C	12.20216200	7.47309200	0.52292300
C	12.48850800	6.45444600	-0.40398500
C	13.72628000	5.82182700	-0.39577800
H	14.17890200	3.59874100	0.81437800
H	15.18216000	7.49172400	2.17499500
H	12.98073500	8.65010700	2.13541800
H	11.75693700	6.19702400	-1.16346300
H	13.94648300	5.06466200	-1.14144700
C	4.89261300	10.39614400	-0.01317000
N	3.79308100	9.82993200	-0.35150900
C	-3.77216600	13.81631300	-0.53539800
C	-5.19063800	15.80509700	-0.57016300
C	-4.16217400	18.06161800	-0.47494300
H	-6.17560200	16.26128400	-0.61819300
H	9.84264600	6.51930000	0.03017400
C	16.25709300	4.14995700	0.49677200
H	-9.38813600	9.97896300	0.05382700
C	-1.59467900	16.90867300	-0.46853400
C	-0.15843700	14.94230900	-0.52311900
C	-1.16980400	12.65356400	-0.51545700
C	0.14277900	11.96468200	-0.46740000
C	0.44561000	10.95618000	-1.39797100

C	1.64428100	10.25749300	-1.33537500
C	2.59966300	10.56516300	-0.35388300
C	2.29374400	11.55500800	0.59721200
C	1.08363400	12.23736700	0.53965100
H	0.83074700	14.50468800	-0.57547800
H	-0.27173300	10.72804700	-2.18031600
H	1.87654000	9.47833300	-2.05372100
H	2.98787900	11.75329800	1.40783300
H	0.84669300	12.97382800	1.30086300
C	8.61870200	8.31591200	0.20130100
C	7.44092500	7.62554600	-0.13706700
C	6.22494700	8.28876400	-0.20699800
C	6.15840000	9.66637600	0.06205300
C	7.33539500	10.35566900	0.40523000
C	8.55134600	9.69300000	0.47333000
H	7.49149700	6.56023900	-0.34705200
H	5.31045700	7.76896100	-0.46994500
H	7.28495300	11.42111100	0.61452800
H	9.46662900	10.21304100	0.73271000
C	9.89089500	7.59464500	0.25543400
N	10.99206200	8.17865600	0.55567700
C	-17.15260700	-0.49058600	0.63828400
C	-18.70141200	1.47284200	0.47860600
C	-17.59579800	2.45930200	0.44864700
C	-17.58225500	3.54368100	1.34248000
C	-16.57679900	4.50099300	1.29472000
C	-15.52925300	4.39139500	0.36655100
C	-15.55151000	3.32569600	-0.55119400
C	-16.56972300	2.38064300	-0.50835800
H	-16.31007400	0.17897300	0.76135400
H	-18.36805600	3.62370800	2.08732200
H	-16.56614500	5.33667600	1.98652800
H	-14.79300700	3.26745300	-1.32544600
H	-16.59092200	1.58108600	-1.24194000
C	-9.09738900	8.94245500	-0.17124400
N	-7.89505400	8.62764400	-0.48582300
C	-3.02960500	18.87125200	-0.43975500
C	-2.60484200	14.64400500	-0.50511100
H	-3.13820200	19.95138300	-0.41773400
C	-2.32577400	11.86963900	-0.55625500
C	-2.74773700	16.06696000	-0.48991700
H	-2.21779200	10.79016200	-0.53008800
C	-19.57546400	-0.82748100	0.47596100
C	-20.02305900	1.94113400	0.46693100

H	-20.19413000	3.01272200	0.43743100
C	2.19609900	-15.15583900	-0.54074500
C	3.29486700	-12.98218800	-0.55177400
C	1.92520900	-10.88852000	-0.50040700
C	3.11778900	-10.00774100	-0.44145500
C	3.29396100	-8.98416400	-1.38818500
C	4.38913500	-8.13030700	-1.33136600
C	5.34850300	-8.29146400	-0.32551500
C	5.17735600	-9.29241500	0.63938500
C	4.07176100	-10.13655900	0.57975000
H	4.20043300	-12.38971300	-0.59257000
H	2.56519700	-8.87247800	-2.18508300
H	4.52886100	-7.33544400	-2.05530400
H	5.89689500	-9.38220400	1.44685100
H	3.93227900	-10.89343600	1.34473400
C	10.65434800	-4.97519300	0.05862100
C	9.30346500	-4.64068100	-0.10376200
C	8.29801100	-5.61869200	-0.16384000
C	8.73850400	-6.97474000	-0.08544200
C	10.09837300	-7.32107100	0.05884400
C	11.05328900	-6.32965000	0.14139800
H	9.03870000	-3.59075300	-0.19615700
H	10.38654900	-8.36858600	0.10763500
H	12.10650300	-6.55366000	0.26211100
C	11.64858700	-3.90469900	0.13244000
N	12.89486800	-4.13983300	0.32590200
C	-16.94645200	-1.83633200	0.64344600
C	-17.83645400	-4.17439500	0.49775000
C	-16.48800300	-4.78939000	0.47910200
C	-16.16684700	-5.82395600	1.37472700
C	-14.92882600	-6.45168900	1.33262200
C	-13.95196400	-6.04557700	0.40951000
C	-14.27257100	-5.02632000	-0.50516200
C	-15.52055100	-4.41474800	-0.46911700
H	-15.94230100	-2.22195000	0.77119200
H	-16.90081700	-6.12862000	2.11435200
H	-14.68300300	-7.25185400	2.02282400
H	-13.55806500	-4.74993100	-1.27419600
H	-15.76630600	-3.65435300	-1.20344100
C	-6.56579200	-8.76683100	-0.11739300
N	-5.46692900	-8.17262200	-0.40760900
C	-0.45250600	-12.45510600	-0.53832900
C	-1.53090200	-14.64632400	-0.61705000
C	-0.15092500	-16.70864100	-0.57237900

H	-2.42947600	-15.25436800	-0.67516800
H	11.26109800	-2.88012700	0.02680200
C	16.69003600	1.30598000	0.42158400
H	-6.60197200	-9.84670600	0.08770400
C	-0.26164900	-15.30927900	-0.57043100
C	-1.62141600	-13.28872600	-0.60975600
C	-0.52588100	-11.03993100	-0.52107000
C	-1.81861200	-10.31421600	-0.47645300
C	-2.09348400	-9.28725000	-1.39496800
C	-3.28900700	-8.58048200	-1.34558000
C	-4.26700500	-8.89858300	-0.39009000
C	-3.98304000	-9.89847700	0.55735100
C	-2.77750500	-10.58877100	0.51292100
H	-2.59218900	-12.81342200	-0.67405800
H	-1.36086800	-9.05537900	-2.16205700
H	-3.50277800	-7.79193500	-2.05943200
H	-4.69411500	-10.10229900	1.35166900
H	-2.56097300	-11.33926500	1.26646300
C	-10.34809100	-6.78906100	0.07259700
C	-9.18451000	-6.06002600	-0.23239900
C	-7.94940800	-6.68839500	-0.29609200
C	-7.85000200	-8.06923500	-0.05595400
C	-9.01377900	-8.79781400	0.24959600
C	-10.24799500	-8.16990400	0.31325300
H	-9.26052100	-4.99183800	-0.41866200
H	-7.04534000	-6.13753900	-0.53055700
H	-8.93768100	-9.86604200	0.43549300
H	-11.15253200	-8.71991400	0.54717300
C	-11.63991300	-6.10345900	0.13337100
N	-12.72500500	-6.72211900	0.42263600
C	15.38273100	1.86902000	0.60981300
C	16.92312700	-0.09196600	0.35706100
C	15.82700600	-1.08979900	0.36559100
C	15.89363900	-2.20193600	1.22343400
C	14.92201900	-3.19265000	1.19289900
C	13.82536500	-3.09415400	0.32087700
C	13.75587300	-1.99032300	-0.54809100
C	14.74335200	-1.01068500	-0.52542900
H	14.54552000	1.19693300	0.75405200
H	16.72124100	-2.28062300	1.92175600
H	14.98010500	-4.05433200	1.84963900
H	12.95272500	-1.93219800	-1.27610900
H	14.69700700	-0.18750900	-1.23137500
C	7.67637300	-7.92255600	-0.16610600

N	6.46825500	-7.42806900	-0.28099600
C	2.02978100	-12.30107100	-0.51216200
C	3.37413000	-14.34048000	-0.55714100
C	2.26036100	-16.55809700	-0.54298400
H	4.34242500	-14.83220300	-0.59034700
H	-11.62083100	-5.02305600	-0.07117300
C	-18.03062700	-2.76941200	0.52001600
H	7.82969200	-9.00058100	-0.16468200
C	0.65902400	-10.29841100	-0.52272000
C	0.92203500	-14.51173900	-0.54395900
H	0.59200000	-9.21581600	-0.47854400
C	1.09785500	-17.32486100	-0.55513300
C	0.83259600	-13.08445100	-0.52860700
H	1.16555700	-18.40851000	-0.55694000
C	-18.95809300	-5.01624900	0.48663000
H	-18.80116300	-6.09010600	0.46281400
C	-19.35889600	-2.24100700	0.48004400
C	18.70209800	4.50834700	0.34463100
C	20.02475200	3.96369600	0.24351700
C	18.47536300	5.89124800	0.39534300
H	20.86326400	4.65322400	0.20124700
C	17.58261200	3.62226800	0.39769700
C	17.18549200	6.39696800	0.47214700
H	17.02852800	7.47107300	0.47308500
C	20.22902000	2.61956600	0.20353500
C	19.32432600	0.31313200	0.23267700
H	21.23414800	2.21397900	0.12863000
C	19.12929600	1.70129600	0.26278100
C	18.24440300	-0.55683900	0.27681000
H	18.41449700	-1.62722000	0.21813700
C	17.79753500	2.20902200	0.35802800
H	-1.05677600	-17.30800600	-0.59143300
H	-0.87561100	18.93558200	-0.42256000
H	-5.15312300	18.50676200	-0.48431300
H	19.32390900	6.56843100	0.35534300
H	20.33519800	-0.07775400	0.15758800
H	3.23390000	-17.04013400	-0.53898100
H	-21.09934600	-5.18726900	0.44387800
H	-22.11754700	1.46322500	0.42891100
Ir	6.30680700	-5.36112300	-0.31881300
C	4.91940700	-4.83189800	1.47033300
C	5.94019000	-3.84471100	1.34321200
C	5.81509300	-3.23712800	0.02962700
C	4.63758100	-3.81176400	-0.59846400

C	4.09773800	-4.79576400	0.27116900
C	2.83827000	-5.58013500	0.07417900
C	4.06966400	-3.37919600	-1.91531100
C	6.55513900	-2.02575800	-0.44965000
C	6.95789300	-3.46678000	2.37389800
C	4.66089700	-5.72238800	2.64715100
H	2.92637100	-6.59163700	0.47630900
H	2.58221900	-5.67038500	-0.98363600
H	1.99548700	-5.09226700	0.58093600
H	3.39322700	-2.52556000	-1.78046700
H	3.50384100	-4.18341600	-2.39069300
H	4.86042700	-3.07499700	-2.60388900
H	6.66156200	-2.02980600	-1.53700000
H	7.55822800	-1.97914900	-0.01887400
H	6.03301800	-1.10280800	-0.16448200
H	6.66009400	-2.54857600	2.89504300
H	7.93763700	-3.29336100	1.92218500
H	7.07823800	-4.25384300	3.12130600
H	5.54678200	-5.81454300	3.27915900
H	4.37637800	-6.72795100	2.32407500
H	3.84455900	-5.33156800	3.26793200
H	6.61716400	-5.45450300	-1.87905300

IM-3 + H_2O

C	-22.06323400	-1.27466000	0.50987700
C	-21.16304000	1.03475600	0.52681900
H	-23.07183100	-0.87168100	0.48433400
C	-20.96423300	-0.35349500	0.53122400
C	-20.52681000	-3.16129600	0.56144200
C	-21.85383400	-2.61865500	0.52413100
C	-20.29402300	-4.54399900	0.58664900
H	-22.69195600	-3.30978500	0.51012300
C	-1.40002200	14.08429400	-0.57034000
C	-0.41229500	16.31861300	-0.59365600
C	-1.87814300	18.32094200	-0.56847100
H	0.46122200	16.96430500	-0.61832500
H	-13.08935300	4.02112700	-0.08478600
C	-18.52195800	0.04513900	0.58291000
H	4.84517300	11.52020600	0.23281300
C	-4.15845800	16.67212600	-0.62236200
C	-5.16501400	14.45513400	-0.68819200
C	-3.71261800	12.41896400	-0.61070500
C	-4.85882800	11.47816700	-0.58756500
C	-4.91777000	10.41442700	-1.50400700

C	-5.94589200	9.48213100	-1.45685500
C	-6.97275300	9.59880700	-0.50664800
C	-6.91031500	10.64811300	0.42768300
C	-5.86737600	11.56671000	0.38655200
H	-6.04307000	13.82577400	-0.76387100
H	-4.14475400	10.32753900	-2.26132400
H	-5.98818500	8.65880600	-2.16212100
H	-7.65441900	10.71052600	1.21552300
H	-5.81180100	12.34862700	1.13716700
C	-12.33401900	6.06094300	0.02890100
C	-11.02416600	5.65500800	-0.28381100
C	-9.99449600	6.58004500	-0.36260800
C	-10.25242100	7.94141600	-0.12861200
C	-11.56198100	8.34752200	0.18484800
C	-12.59235000	7.42237800	0.26192300
H	-10.82339400	4.60201000	-0.46378300
H	-8.98055400	6.28058600	-0.60290700
H	-11.76250700	9.40026900	0.36651900
H	-13.60627400	7.72229000	0.50197800
C	-13.39764600	5.05954200	0.10532000
N	-14.60998600	5.36433200	0.39100400
C	15.08613000	3.29554100	0.82833500
C	15.97396100	5.63640400	0.70986400
C	14.62752400	6.25561900	0.71252700
C	14.31991600	7.28368500	1.62039400
C	13.09085000	7.92898000	1.58494300
C	12.10964100	7.54866000	0.65546500
C	12.41469600	6.53129500	-0.26681300
C	13.65330700	5.90092400	-0.23640900
H	14.08423100	3.67882900	0.97971500
H	15.05889600	7.57175200	2.36169400
H	12.85662700	8.72635700	2.28240500
H	11.69736900	6.27288500	-1.03941300
H	13.88831900	5.14463200	-0.97843300
C	4.79877200	10.44507200	0.00578700
N	3.70426000	9.87215600	-0.33746700
C	-3.87489700	13.82628500	-0.61516600
C	-5.30052200	15.80909100	-0.68312000
C	-4.28086600	18.07053500	-0.61243800
H	-6.28690900	16.26090500	-0.74208000
H	9.76175000	6.58649200	0.12880600
C	16.16795300	4.23126700	0.70322300
H	-9.47839100	9.97590000	0.00872900
C	-1.70919300	16.92738700	-0.57758600

C	-0.26547000	14.96584900	-0.59873600
C	-1.26846000	12.67371200	-0.56711300
C	0.04654600	11.99076900	-0.50280900
C	0.35984000	10.97316400	-1.41984800
C	1.56097700	10.28030600	-1.34117500
C	2.50817800	10.60277500	-0.35649600
C	2.19157500	11.60194900	0.58130200
C	0.97927800	12.27877600	0.50751400
H	0.72556100	14.53119700	-0.63989100
H	-0.35121600	10.73314700	-2.20437900
H	1.80142900	9.49406900	-2.04902900
H	2.87904200	11.81214400	1.39459500
H	0.73406000	13.02294400	1.25855600
C	8.52920500	8.37939600	0.27601300
C	7.35828600	7.68311500	-0.07383200
C	6.14092100	8.34162100	-0.16207800
C	6.06624500	9.72034200	0.09906100
C	7.23654400	10.41572400	0.45272800
C	8.45377400	9.75767000	0.53976600
H	7.41516100	6.61698500	-0.27786600
H	5.23160800	7.81712900	-0.43349700
H	7.17979200	11.48209400	0.65560400
H	9.36388300	10.28225100	0.80806300
C	9.80287800	7.66268100	0.35134400
N	10.89739600	8.25115700	0.66692400
C	-17.20620000	-0.51523500	0.71028800
C	-18.76077400	1.44277500	0.53988300
C	-17.65808900	2.43204900	0.49714700
C	-17.64505500	3.52464600	1.38095600
C	-16.64292600	4.48472100	1.32102700
C	-15.59807100	4.36993000	0.39044700
C	-15.61976900	3.29566100	-0.51724100
C	-16.63485200	2.34781300	-0.46241200
H	-16.36489900	0.15751500	0.82400400
H	-18.42864300	3.60902200	2.12764400
H	-16.63276400	5.32682200	2.00501500
H	-14.86350600	3.23268500	-1.29331700
H	-16.65587300	1.54152700	-1.18859500
C	-9.18330300	8.93789900	-0.20341900
N	-7.97919300	8.62386700	-0.51193000
C	-3.15146700	18.88478400	-0.58205700
C	-2.71084200	14.65870600	-0.58986700
H	-3.26414100	19.96470000	-0.57481600
C	-2.42122700	11.88489600	-0.60370100

C	-2.85904400	16.08118100	-0.59397500
H	-2.30932000	10.80625800	-0.56312500
C	-19.62882200	-0.85975000	0.56112700
C	-20.08372000	1.90746700	0.52934900
H	-20.25774400	2.97829400	0.49099400
C	2.18326000	-15.13436900	-0.49245700
C	3.27083500	-12.95545200	-0.53162000
C	1.89072200	-10.86818900	-0.48205000
C	3.07817000	-9.98000400	-0.43984400
C	3.23471400	-8.95572100	-1.38906000
C	4.32477300	-8.09359500	-1.34766500
C	5.29724800	-8.24510500	-0.35320700
C	5.14537400	-9.24716700	0.61447200
C	4.04716600	-10.10068600	0.56861300
H	4.17318900	-12.35927500	-0.58707300
H	2.49662800	-8.85041800	-2.17810500
H	4.45183300	-7.30415400	-2.07908100
H	5.87035900	-9.32733700	1.41798500
H	3.92284000	-10.85647800	1.33715900
C	10.58244900	-4.89744200	0.11240000
C	9.23796200	-4.56602300	-0.10923000
C	8.23541100	-5.54570300	-0.20627100
C	8.67426000	-6.89817900	-0.07776300
C	10.02701700	-7.24173900	0.12532100
C	10.97953700	-6.24918300	0.22591000
H	8.97758200	-3.51698600	-0.20859500
H	10.31194000	-8.28815500	0.20553400
H	12.02742100	-6.47093700	0.38919100
C	11.57154100	-3.82315900	0.21262100
N	12.81165500	-4.05654300	0.44236500
C	-16.99651100	-1.86035900	0.72618400
C	-17.88106000	-4.20188000	0.60454900
C	-16.53115800	-4.81361300	0.58433800
C	-16.20280600	-5.83999300	1.48674600
C	-14.96362600	-6.46535000	1.44306000
C	-13.99275000	-6.06480700	0.51124700
C	-14.32055200	-5.05371800	-0.40993100
C	-15.56964300	-4.44457600	-0.37209200
H	-15.99081100	-2.24226900	0.85293000
H	-16.93208800	-6.14019800	2.23280100
H	-14.71229900	-7.25929100	2.13845000
H	-13.61092800	-4.78214200	-1.18517000
H	-15.82109700	-3.69081600	-1.11130400
C	-6.60500100	-8.77623300	-0.04433200

N	-5.50993200	-8.18193400	-0.34832800
C	-0.47893700	-12.44704900	-0.48612000
C	-1.54679100	-14.64414100	-0.53680000
C	-0.15597200	-16.69898900	-0.48881300
H	-2.44273200	-15.25716500	-0.58141800
H	11.18508000	-2.79996700	0.09128600
C	16.60352100	1.38766500	0.63335600
H	-6.63711000	-9.85451400	0.16972800
C	-0.27377200	-15.30023400	-0.49699800
C	-1.64420200	-13.28700900	-0.53970800
C	-0.55970600	-11.03219000	-0.47978500
C	-1.85543100	-10.31210100	-0.43058500
C	-2.14179600	-9.29402900	-1.35549500
C	-3.33934900	-8.59112600	-1.30189800
C	-4.30799000	-8.90444500	-0.33533100
C	-4.01264300	-9.89562000	0.61777200
C	-2.80503400	-10.58208500	0.56891200
H	-2.61788100	-12.81702400	-0.59867000
H	-1.41659600	-9.06593600	-2.13072100
H	-3.56187300	-7.80924800	-2.02037400
H	-4.71635100	-10.09536100	1.41964900
H	-2.57954200	-11.32547100	1.32687600
C	-10.38962600	-6.80424300	0.15781700
C	-9.22974600	-6.07559400	-0.16182600
C	-7.99395600	-6.70215300	-0.22939400
C	-7.89011300	-8.08074100	0.02159500
C	-9.05012000	-8.80890000	0.34209000
C	-10.28506400	-8.18278100	0.40955300
H	-9.30922100	-5.00919700	-0.35667100
H	-7.09277200	-6.15162000	-0.47541100
H	-8.97059500	-9.87538400	0.53639300
H	-11.18680300	-8.73249600	0.65469700
C	-11.68235900	-6.12058400	0.22139500
N	-12.76436400	-6.73882400	0.52283500
C	15.29252800	1.94989300	0.79681700
C	16.83876700	-0.00998500	0.57091900
C	15.74289000	-1.00789700	0.55362400
C	15.79190000	-2.12350800	1.40805200
C	14.82111400	-3.11408000	1.35315000
C	13.74254600	-3.01087200	0.45965100
C	13.69062300	-1.90351900	-0.40597900
C	14.67770200	-0.92465000	-0.35925000
H	14.45270600	1.27764600	0.92410400
H	16.60504400	-2.20528700	2.12272900

H	14.86562500	-3.97869800	2.00706400
H	12.90291500	-1.84122000	-1.15032200
H	14.64606700	-0.09870000	-1.06262800
C	7.61731800	-7.85158200	-0.16057200
N	6.40962500	-7.37160000	-0.32049300
C	2.00285800	-12.28026100	-0.48423600
C	3.35688800	-14.31334900	-0.52724200
C	2.25465400	-16.53625300	-0.48421300
H	4.32726200	-14.80046400	-0.56678500
H	-11.66667500	-5.04195500	0.00748000
C	-18.07877300	-2.79725800	0.61554400
H	7.77702900	-8.92785900	-0.11880200
C	0.62126200	-10.28483400	-0.49776100
C	0.90604300	-14.49661500	-0.48828700
H	0.54849000	-9.20234000	-0.46156900
C	1.09599300	-17.30887100	-0.47883100
C	0.80949800	-13.06972600	-0.48330300
H	1.16912900	-18.39213500	-0.47270200
C	-19.00053200	-5.04668400	0.60589000
H	-18.84092000	-6.12030100	0.59064900
C	-19.40856900	-2.27262800	0.57676900
C	18.61523200	4.59084800	0.59965000
C	19.93987500	4.04685800	0.52440800
C	18.38679700	5.97359100	0.64658400
H	20.77873600	4.73679600	0.49934800
C	17.49537100	3.70425800	0.62992200
C	17.09539400	6.47867500	0.69801300
H	16.93792700	7.55269000	0.69631200
C	20.14562500	2.70287300	0.48727400
C	19.24165700	0.39601000	0.49554500
H	21.15222800	2.29784400	0.43189300
C	19.04533500	1.78405500	0.52365900
C	18.16145700	-0.47447100	0.51663000
H	18.33324800	-1.54466900	0.45929800
C	17.71171900	2.29117200	0.59310400
H	-1.05891100	-17.30298500	-0.49428000
H	-0.99784000	18.95740500	-0.55447300
H	-5.27338000	18.51181200	-0.63276000
H	19.23560900	6.65123000	0.62367700
H	20.25398900	0.00568800	0.44004600
H	3.23060500	-17.01338400	-0.48606000
H	-21.14150000	-5.22355500	0.57421900
H	-22.17707700	1.42384700	0.50412700
Ir	6.24407400	-5.30328300	-0.46879000

C	4.55172500	-4.84294700	1.04830700
C	5.65205100	-3.96799800	1.30677100
C	5.87411100	-3.17950200	0.10856800
C	4.86433200	-3.55195800	-0.85266500
C	4.04071400	-4.57599700	-0.27848600
C	2.78323300	-5.14070700	-0.86253200
C	4.65540700	-2.88556500	-2.17839100
C	6.79979400	-2.00788600	-0.01897400
C	6.43431000	-3.84646300	2.57795300
C	3.96911500	-5.84606700	1.99593200
H	2.62163600	-6.17362000	-0.54710700
H	2.81199000	-5.12875000	-1.95501200
H	1.90817200	-4.55659000	-0.54847300
H	4.00022000	-2.01309900	-2.06055700
H	4.18510300	-3.56349900	-2.89394700
H	5.60117200	-2.54655700	-2.60425500
H	7.26505900	-1.98399400	-1.00723300
H	7.58970000	-2.04953700	0.73479100
H	6.25242300	-1.06909600	0.13359000
H	6.08558200	-2.99528500	3.17635500
H	7.49785900	-3.69851700	2.37318900
H	6.33956800	-4.74567600	3.19087400
H	4.69494200	-6.14762000	2.75430400
H	3.64434100	-6.74661100	1.46909900
H	3.09630600	-5.42891300	2.51289700
H	6.54181600	-5.55355600	-2.01894900
O	7.97840000	-3.29322700	-2.85260300
H	8.88105800	-3.48119900	-2.56680800
H	7.46916400	-4.00448800	-2.42286500

IM-3 + HCOOH

C	22.15719700	-1.47410200	-0.43385900
C	21.29037900	0.84789000	-0.46870800
H	23.17133200	-1.08552800	-0.40468100
C	21.07170700	-0.53739300	-0.46677700
C	20.59409500	-3.33877100	-0.48368300
C	21.92854300	-2.81501600	-0.44163400
C	20.34156800	-4.71811600	-0.50204200
H	22.75653500	-3.51801600	-0.41869600
C	1.66120500	14.11201800	0.52605700
C	0.69022900	16.35373300	0.54585600
C	2.17091000	18.34502000	0.51498400
H	-0.17842900	17.00596300	0.57012900
H	13.25640700	3.94184600	0.08742000

C	18.63581900	-0.10411100	-0.53471600
H	-4.60473900	11.59504300	-0.24343400
C	4.43891200	16.67939700	0.57078900
C	5.42901400	14.45516400	0.64110700
C	3.96145400	12.42971200	0.56899200
C	5.10024000	11.47987200	0.54944500
C	5.14913100	10.41825900	1.46897000
C	6.16920700	9.47707400	1.42555500
C	7.19809800	9.58229900	0.47620900
C	7.14593000	10.62967400	-0.46089600
C	6.11075500	11.55723900	-0.42358500
H	6.30241800	13.81948600	0.71784300
H	4.37450100	10.34009800	2.22558000
H	6.20360800	8.65527500	2.13302700
H	7.89155600	10.68362800	-1.24792900
H	6.06272500	12.33746500	-1.17647400
C	12.52424800	5.98920900	-0.03631100
C	11.20914400	5.59811700	0.27326300
C	10.18908800	6.53417100	0.34573900
C	10.46209400	7.89198000	0.10817200
C	11.77678700	8.28322300	-0.20265800
C	12.79765500	7.34701900	-0.27319300
H	10.99674400	4.54789600	0.45609400
H	9.17130600	6.24628800	0.58390700
H	11.98897500	9.33318200	-0.38719400
H	13.81547400	7.63549900	-0.51079500
C	13.57718800	4.97610000	-0.10469500
N	14.79435400	5.26604400	-0.38533500
C	-14.95242100	3.48605500	-0.76885900
C	-15.80175800	5.83710500	-0.58624100
C	-14.44692500	6.43705100	-0.61289700
C	-14.14752900	7.47777500	-1.50899200
C	-12.90950800	8.10651200	-1.49142000
C	-11.91150400	7.69670200	-0.59287500
C	-12.20752400	6.66548800	0.31694400
C	-13.45454400	6.05151200	0.30475300
H	-13.94951700	3.85725700	-0.94213700
H	-14.90038700	7.78938400	-2.22642100
H	-12.68162100	8.91401400	-2.17928000
H	-11.47550800	6.38301700	1.06699700
H	-13.68139500	5.28401000	1.03772900
C	-4.56556600	10.51817400	-0.02324700
N	-3.47323600	9.93482000	0.30929200
C	4.13423800	13.83576300	0.57008700

C	5.57458000	15.80806100	0.63283300
C	4.57172100	18.07683100	0.55774600
H	6.56432500	16.25268200	0.69021000
H	-9.56264400	6.70292700	-0.12504900
C	-16.01628900	4.43498700	-0.59770800
H	9.70867300	9.93386200	-0.03787900
C	1.99160100	16.95278200	0.52721600
C	0.53332900	15.00211700	0.55389900
C	1.51910500	12.70242100	0.52552900
C	0.19929400	12.02857800	0.46419300
C	-0.11812600	11.01272400	1.38185500
C	-1.32387000	10.32766000	1.30645800
C	-2.27194000	10.65686900	0.32485000
C	-1.95163000	11.65431900	-0.61345200
C	-0.73448100	12.32292700	-0.54330200
H	-0.46088000	14.57501700	0.59712900
H	0.59348900	10.76794300	2.16440800
H	-1.56735100	9.54280700	2.01482000
H	-2.64050800	11.86945200	-1.42426700
H	-0.48662500	13.06549500	-1.29505500
C	-8.31619100	8.48647400	-0.26984200
C	-7.14679100	7.77646700	0.05675300
C	-5.92283800	8.42379200	0.13657600
C	-5.83966100	9.80455400	-0.11082700
C	-7.00818900	10.51332000	-0.44314600
C	-8.23212300	9.86666300	-0.52074900
H	-7.21025100	6.70874000	0.25019100
H	-5.01479600	7.88879500	0.39126100
H	-6.94480600	11.58120400	-0.63588500
H	-9.14118800	10.40174700	-0.77123500
C	-9.59761000	7.78230000	-0.33242400
N	-10.69185400	8.38587500	-0.61914100
C	17.31293600	-0.64616400	-0.66739000
C	18.89438000	1.29016100	-0.49708100
C	17.80534100	2.29476000	-0.46402700
C	17.81068900	3.38341500	-1.35273500
C	16.82080900	4.35670700	-1.30183400
C	15.77027000	4.25966800	-0.37565800
C	15.77409600	3.18960800	0.53725000
C	16.77692200	2.22841400	0.49137500
H	16.48215000	0.03801400	-0.78989700
H	18.59866700	3.45412900	-2.09622300
H	16.82465900	5.19559200	-1.98982800
H	15.01364500	3.14016100	1.31019900

H	16.78417400	1.42525400	1.22127800
C	9.40301800	8.89958200	0.17743600
N	8.19555600	8.59828700	0.48546700
C	3.44839900	18.89940300	0.52639200
C	2.97632200	14.67670400	0.54346800
H	3.56910100	19.97843300	0.51672700
C	2.66612800	11.90531300	0.56312900
C	3.13512000	16.09805200	0.54449900
H	2.54627100	10.82746100	0.52475200
C	19.72937100	-1.02464900	-0.50175200
C	20.22372700	1.73598300	-0.48168600
H	20.41285600	2.80439700	-0.44770400
C	-2.21235900	-15.07062100	0.60643500
C	-3.29766700	-12.89062600	0.56378700
C	-1.91450200	-10.80705900	0.46247400
C	-3.10110300	-9.92094200	0.37642800
C	-3.27308000	-8.86872300	1.29247200
C	-4.36485100	-8.01189000	1.20993700
C	-5.32221600	-8.19452500	0.20502100
C	-5.15656600	-9.22671700	-0.72664600
C	-4.05725700	-10.07590700	-0.63915400
H	-4.19995500	-12.29238300	0.59006400
H	-2.54760400	-8.73978700	2.08981300
H	-4.50429900	-7.20275200	1.91698500
H	-5.87098000	-9.33461900	-1.53651800
H	-3.92197200	-10.85602400	-1.38109100
C	-10.56081800	-4.79048200	-0.32022800
C	-9.21049500	-4.47411800	-0.12563800
C	-8.22072700	-5.46489700	-0.03434300
C	-8.67842500	-6.81318500	-0.14650300
C	-10.03798200	-7.14069700	-0.33975800
C	-10.97810200	-6.13689300	-0.43261400
H	-8.94404400	-3.43045300	-0.01082900
H	-10.33582800	-8.18412300	-0.41152200
H	-12.03121300	-6.34515800	-0.57992400
C	-11.53337500	-3.69861500	-0.37760800
N	-12.78225100	-3.90854000	-0.58484800
C	17.08399600	-1.98819200	-0.67765500
C	17.93385600	-4.34153400	-0.53691600
C	16.57533600	-4.93403200	-0.52182900
C	16.23954400	-5.96321400	-1.41837900
C	14.99182800	-6.57154600	-1.37875000
C	14.01963100	-6.15008700	-0.45759200
C	14.35412600	-5.13562700	0.45738800

C	15.61180600	-4.54404400	0.42421400
H	16.07374000	-2.35622400	-0.80901300
H	16.97007000	-6.27937300	-2.15657700
H	14.73503300	-7.36792000	-2.06934400
H	13.64230500	-4.84798700	1.22479400
H	15.86797500	-3.78787100	1.15931700
C	6.59610600	-8.76522200	0.07024800
N	5.50315500	-8.15418700	0.34775300
C	0.45283400	-12.38708300	0.54508400
C	1.51751000	-14.58231400	0.67706300
C	0.12488600	-16.63649800	0.67893800
H	2.41218700	-15.19436500	0.75085600
H	-11.12536100	-2.68747900	-0.23578200
C	-16.49200000	1.59718900	-0.56277300
H	6.62016100	-9.84819200	-0.11977600
C	0.24424100	-15.23828900	0.64398300
C	1.61649900	-13.22586200	0.63810000
C	0.53538600	-10.97319100	0.49525900
C	1.83350300	-10.25774800	0.43991500
C	2.11523300	-9.21790900	1.34158800
C	3.31775800	-8.52363200	1.28393800
C	4.29538400	-8.86721300	0.33692500
C	4.00404700	-9.87943900	-0.59511300
C	2.79202100	-10.55766500	-0.54234600
H	2.59004700	-12.75507400	0.69262400
H	1.38295000	-8.96652500	2.10285800
H	3.53737600	-7.72564800	1.98540600
H	4.71526600	-10.10337600	-1.38384700
H	2.57056400	-11.31919100	-1.28332000
C	10.40451300	-6.83921300	-0.12657200
C	9.24987200	-6.09192900	0.16802600
C	8.00616800	-6.70306300	0.23291400
C	7.88912000	-8.08461200	0.00513700
C	9.04399200	-8.83141100	-0.29008300
C	10.28663400	-8.22062200	-0.35560300
H	9.33966300	-5.02330500	0.34540200
H	7.10889100	-6.13802000	0.45958800
H	8.95424500	-9.90022700	-0.46626500
H	11.18447500	-8.78473500	-0.58157100
C	11.70613200	-6.17222200	-0.18778300
N	12.78264200	-6.80799000	-0.47182600
C	-15.17792900	2.14316200	-0.75431700
C	-16.74593400	0.20206900	-0.51674400
C	-15.66568200	-0.81229100	-0.55021400

C	-15.75997500	-1.91237200	-1.42078600
C	-14.80343000	-2.91811500	-1.41386500
C	-13.69510900	-2.84731100	-0.55419500
C	-13.59741400	-1.75522100	0.32708400
C	-14.56985000	-0.76073000	0.32826600
H	-14.35217400	1.46117000	-0.91669700
H	-16.59680300	-1.96955800	-2.11013200
H	-14.88239700	-3.77077700	-2.08015500
H	-12.78270600	-1.71542200	1.04336300
H	-14.50152600	0.05331300	1.04289200
C	-7.63920700	-7.78007900	-0.03857700
N	-6.42580900	-7.31428600	0.12065900
C	-2.02840100	-12.21828900	0.50901300
C	-3.38528200	-14.24788200	0.60189400
C	-2.28534400	-16.47198700	0.64197500
H	-4.35664400	-14.73260200	0.64619600
H	11.70239400	-5.09076900	0.01198800
C	18.15187400	-2.93996600	-0.55491100
H	-7.81456800	-8.85476600	-0.05178600
C	-0.64461100	-10.22444700	0.47514700
C	-0.93443900	-14.43439600	0.59643200
H	-0.56995900	-9.14368900	0.40544700
C	-1.12761000	-17.24534800	0.67423200
C	-0.83615300	-13.00846700	0.54717100
H	-1.20196300	-18.32819300	0.70169900
C	19.04113000	-5.20225700	-0.52616000
H	18.86607600	-6.27336400	-0.50533200
C	19.48885500	-2.43428400	-0.51090800
C	-18.45419000	4.82820000	-0.42006300
C	-19.78415500	4.30223300	-0.31618500
C	-18.20664400	6.20811300	-0.45126500
H	-20.61165700	5.00369500	-0.25657300
C	-17.34890900	3.92609000	-0.49597600
C	-16.90975100	6.69523700	-0.53002900
H	-16.73625100	7.76669100	-0.51494800
C	-20.00861400	2.96089500	-0.29446300
C	-19.13959200	0.64170500	-0.36502600
H	-21.01918300	2.56951000	-0.21707900
C	-18.92360500	2.02704000	-0.37664300
C	-18.07351200	-0.24372600	-0.43133500
H	-18.25947600	-1.31213200	-0.38657000
C	-17.58505900	2.51582100	-0.47593600
H	1.02698500	-17.24076900	0.71373900
H	1.29535100	18.98797700	0.50031300

H	5.56751200	18.51073700	0.57644600
H	-19.04436800	6.89743900	-0.39400600
H	-20.15565000	0.26513400	-0.28667700
H	-3.26181700	-16.94800300	0.64796900
H	21.17906800	-5.40970300	-0.48035300
H	22.30975700	1.22252700	-0.44237000
Ir	-6.22079000	-5.24817800	0.22048100
C	-4.24500800	-4.95971500	-1.00791500
C	-5.32616600	-4.29847200	-1.69228400
C	-5.83984800	-3.28248700	-0.80684700
C	-5.08657600	-3.33649500	0.42052500
C	-4.07676600	-4.37058200	0.28198900
C	-2.98744600	-4.66753500	1.26637000
C	-5.16695700	-2.35153500	1.54679800
C	-6.85553400	-2.24496600	-1.16830900
C	-5.76851600	-4.54637900	-3.10140800
C	-3.41068500	-6.06005200	-1.58686600
H	-2.65566000	-5.70592600	1.19501400
H	-3.32463500	-4.49411500	2.29116600
H	-2.11256000	-4.02770100	1.09215400
H	-4.42260800	-1.55703800	1.40824500
H	-4.96717700	-2.83078200	2.50822300
H	-6.15213500	-1.88688000	1.59699900
H	-7.37309200	-1.86808400	-0.28529500
H	-7.60340600	-2.64524400	-1.85647500
H	-6.36366000	-1.39963600	-1.66580600
H	-5.21613000	-3.91560100	-3.81049100
H	-6.83246800	-4.33102300	-3.22368700
H	-5.60642200	-5.58773500	-3.39097700
H	-4.00814900	-6.72567900	-2.21409300
H	-2.94740900	-6.66830100	-0.80881600
H	-2.61206000	-5.64104900	-2.21086200
H	-6.44945400	-5.48146300	1.78490800
O	-8.10236800	-3.83941000	2.93204400
H	-7.60892100	-4.13457600	2.12446700
C	-8.65859800	-2.64676200	2.77577800
O	-8.57987000	-1.92337200	1.80305100
H	-9.22451100	-2.37835400	3.68213000

TS2

C	-21.99958600	-1.24075200	0.85373800
C	-21.09360300	1.06628500	0.83454700
H	-23.00749300	-0.83524600	0.85155000
C	-20.89810700	-0.32241500	0.84224700

C	-20.46701800	-3.13103400	0.87603200
C	-21.79320700	-2.58518900	0.86969400
C	-20.23712900	-4.51420600	0.90148300
H	-22.63309900	-3.27422800	0.88045000
C	-1.37048000	14.11117600	-0.82970900
C	-0.38838000	16.34599400	-0.92591400
C	-1.85926400	18.34449500	-0.96253100
H	0.48346600	16.99273300	-0.97256400
H	-13.03177400	4.03708800	0.00962200
C	-18.45426800	0.07025200	0.83038000
H	4.87975300	11.57731600	0.07978400
C	-4.13553500	16.68914400	-0.95880900
C	-5.13663200	14.46868900	-0.95091400
C	-3.67888300	12.43975900	-0.81286800
C	-4.82295000	11.49773400	-0.75611700
C	-4.88488200	10.40734600	-1.64035400
C	-5.91087800	9.47494100	-1.55957800
C	-6.93206300	9.61719000	-0.60671700
C	-6.86618800	10.69375300	0.29592200
C	-5.82592400	11.61317300	0.22093000
H	-6.01320100	13.83491000	-1.00373000
H	-4.11600400	10.29952900	-2.39920900
H	-5.95573300	8.63105400	-2.23993700
H	-7.60525500	10.77792500	1.08645800
H	-5.76769200	12.41739300	0.94741400
C	-12.27717600	6.07963300	0.07581600
C	-10.97162100	5.67021400	-0.25002400
C	-9.94540600	6.59555700	-0.36327700
C	-10.20281300	7.96084200	-0.15260600
C	-11.50828100	8.37050800	0.17309800
C	-12.53495500	7.44493600	0.28566000
H	-10.77130300	4.61427200	-0.41237100
H	-8.93465600	6.29334500	-0.61337700
H	-11.70846700	9.42631300	0.33649600
H	-13.54558300	7.74747700	0.53608200
C	-13.33738900	5.07807100	0.18975900
N	-14.54387700	5.38611700	0.49588300
C	15.06010900	3.32117200	1.01560500
C	15.97110100	5.65227100	0.87963300
C	14.63043400	6.28261000	0.83966400
C	14.31073800	7.32869000	1.72248900
C	13.08778400	7.98225900	1.64867800
C	12.12428000	7.59246100	0.70474600
C	12.44204600	6.55754200	-0.19331400

C	13.67505600	5.91909100	-0.12488700
H	14.05815200	3.71534600	1.13529900
H	15.03515400	7.62399200	2.47521700
H	12.84429700	8.79310300	2.32716900
H	11.73964300	6.29122800	-0.97689300
H	13.92085000	5.14865900	-0.84866100
C	4.83304000	10.49656500	-0.11851400
N	3.74026200	9.91573500	-0.45382900
C	-3.84467800	13.84579300	-0.86141300
C	-5.27556700	15.82177700	-0.98892500
C	-4.26145100	18.08684400	-0.99349900
H	-6.26324400	16.26905400	-1.05964400
H	9.78278100	6.62962000	0.15987400
C	16.15286200	4.24563700	0.90203900
H	-9.43365700	9.99995000	-0.07347900
C	-1.68679200	16.95179100	-0.92725400
C	-0.23814500	14.99412100	-0.88784800
C	-1.23547000	12.70177400	-0.78189100
C	0.08122900	12.02457100	-0.69560400
C	0.39803400	10.97919400	-1.57954000
C	1.60006200	10.29105900	-1.47637400
C	2.54438800	10.64547900	-0.49995000
C	2.22449300	11.67373500	0.40477100
C	1.01160000	12.34624500	0.30674200
H	0.75393500	14.56079500	-0.91642600
H	-0.31119600	10.71311600	-2.35728400
H	1.84310800	9.48295800	-2.15824800
H	2.90941500	11.91045200	1.21290000
H	0.76358000	13.11351700	1.03320600
C	8.55483100	8.42953000	0.24567500
C	7.38615700	7.72780100	-0.10074600
C	6.17185200	8.38695200	-0.22001300
C	6.09814200	9.77208100	0.00504000
C	7.26640500	10.47332100	0.35378800
C	8.48053800	9.81439900	0.47250000
H	7.44224400	6.65655300	-0.27630000
H	5.26400100	7.85811600	-0.48785900
H	7.21045500	11.54468600	0.52855900
H	9.38881200	10.34328200	0.73858200
C	9.82446300	7.71063900	0.35786300
N	10.91615200	8.30161900	0.67842300
C	-17.13722400	-0.49297400	0.92876400
C	-18.69070100	1.46824900	0.78442300
C	-17.58774400	2.45502700	0.70654100

C	-17.55371500	3.55703900	1.57815100
C	-16.55355600	4.51635700	1.48423400
C	-15.53080500	4.39125800	0.53070000
C	-15.57314300	3.30675400	-0.36392100
C	-16.58689900	2.35996500	-0.27538400
H	-16.29170200	0.17807200	1.01879800
H	-18.31953100	3.64972400	2.34208600
H	-16.52769300	5.36599300	2.15842400
H	-14.83490600	3.23512500	-1.15644600
H	-16.62497200	1.54612400	-0.99235500
C	-9.13755200	8.95798600	-0.26362400
N	-7.93580600	8.63988300	-0.57725100
C	-3.13405300	18.90443300	-0.99166200
C	-2.68272900	14.68146800	-0.86521200
H	-3.24946400	19.98374000	-1.01885300
C	-2.38622900	11.90921200	-0.79180600
C	-2.83454400	16.10268600	-0.91436500
H	-2.27163000	10.83265700	-0.71733600
C	-19.56357800	-0.83190200	0.84143900
C	-20.01248300	1.93614300	0.80422200
H	-20.18495400	3.00711200	0.76307400
C	2.18996400	-15.14383300	-0.91694800
C	3.27611300	-12.96383600	-0.90731600
C	1.89738000	-10.88035200	-0.76356200
C	3.08372800	-9.99201700	-0.70987500
C	3.21691800	-8.92486200	-1.61257300
C	4.30434100	-8.05847600	-1.56031200
C	5.30080400	-8.24905400	-0.59410900
C	5.16405700	-9.29164600	0.33742400
C	4.07246700	-10.14980700	0.27513600
H	4.17680800	-12.36505300	-0.96159700
H	2.46099400	-8.78475400	-2.37905300
H	4.40025800	-7.22841300	-2.25061000
H	5.89399700	-9.39734200	1.13344700
H	3.96467800	-10.93484600	1.01643800
C	10.51914700	-4.85916700	0.28205600
C	9.17912300	-4.52785300	0.04151400
C	8.18301000	-5.50847800	-0.06488800
C	8.61963300	-6.86422500	0.00579500
C	9.96680400	-7.20440200	0.24683200
C	10.91143700	-6.21155900	0.40190800
H	8.91911400	-3.47956800	-0.07138200
H	10.25489100	-8.25152600	0.29667500
H	11.95429400	-6.43407400	0.59338600

C	11.50754100	-3.78611600	0.40096700
N	12.73850600	-4.02211500	0.67166900
C	-16.93055300	-1.83856700	0.94634100
C	-17.82353400	-4.17843400	0.85628100
C	-16.47644900	-4.79431900	0.80292500
C	-16.12912800	-5.82171900	1.69715400
C	-14.89429800	-6.45257300	1.62178800
C	-13.94603800	-6.05648700	0.66508600
C	-14.29285800	-5.04374200	-0.24720700
C	-15.53808700	-4.42938600	-0.17771100
H	-15.92301900	-2.22245100	1.05059300
H	-16.84017000	-6.11864200	2.46190300
H	-14.62881300	-7.24759900	2.31065400
H	-13.60192600	-4.77539300	-1.04025900
H	-15.80521000	-3.67497600	-0.91072900
C	-6.58580300	-8.79314000	-0.09962700
N	-5.49549900	-8.19672100	-0.41606000
C	-0.47213600	-12.46019100	-0.77519800
C	-1.54043900	-14.65538700	-0.87510000
C	-0.14858600	-16.70970500	-0.91869700
H	-2.43698700	-15.26738400	-0.92203600
H	11.12882500	-2.76320900	0.25614400
C	16.56515500	1.39764400	0.89162300
H	-6.61819600	-9.87520400	0.09423400
C	-0.26678700	-15.31146100	-0.88029000
C	-1.63802300	-13.29886400	-0.83327500
C	-0.55266900	-11.04628200	-0.72288100
C	-1.84712600	-10.32831000	-0.62858900
C	-2.14874800	-9.28250700	-1.51695500
C	-3.34413900	-8.58062800	-1.42014000
C	-4.29536000	-8.92182400	-0.44576700
C	-3.98423600	-9.94178200	0.47133300
C	-2.77886700	-10.62775200	0.37935600
H	-2.61272400	-12.82791700	-0.85884400
H	-1.43703400	-9.03108900	-2.29738800
H	-3.57846700	-7.77684100	-2.11011200
H	-4.67296300	-10.16497700	1.28002000
H	-2.54046000	-11.39386100	1.11027200
C	-10.35601300	-6.80843200	0.21910700
C	-9.20056100	-6.08037600	-0.11746300
C	-7.96947700	-6.71105800	-0.22190200
C	-7.86604500	-8.09348400	0.00733000
C	-9.02161400	-8.82107700	0.34467500
C	-10.25178200	-8.19066700	0.44980500

H	-9.27976200	-5.01113300	-0.29621300
H	-7.07167300	-6.16112900	-0.48122300
H	-8.94250000	-9.89054000	0.52204000
H	-11.15001700	-8.74000900	0.70834400
C	-11.64385900	-6.12032000	0.32059700
N	-12.72105400	-6.73632300	0.64319200
C	15.25541100	1.97350000	1.01185800
C	16.78977500	-0.00271700	0.85861700
C	15.68617700	-0.99169600	0.82650200
C	15.70237800	-2.09514800	1.69783100
C	14.72644700	-3.07978300	1.62893900
C	13.67491400	-2.98176600	0.70327500
C	13.65521300	-1.88615300	-0.17842400
C	14.64774900	-0.91366900	-0.11719200
H	14.40679300	1.31050500	1.12922600
H	16.49439200	-2.17256900	2.43628200
H	14.74634800	-3.93557700	2.29553000
H	12.89003600	-1.82998600	-0.94640100
H	14.64275500	-0.09773200	-0.83283000
C	7.59595300	-7.82824500	-0.23457000
N	6.40626100	-7.37257300	-0.52445200
C	2.00918000	-12.29159600	-0.81387900
C	3.36255200	-14.32113200	-0.94804100
C	2.26168900	-16.54524500	-0.95460200
H	4.33207300	-14.80594100	-1.02216000
H	-11.62905400	-5.03995100	0.11537600
C	-18.01746100	-2.77323600	0.86662900
H	7.78145700	-8.90109400	-0.19417500
C	0.62749600	-10.29781400	-0.73782500
C	0.91287200	-14.50757400	-0.86834300
H	0.55563700	-9.21717900	-0.66496600
C	1.10347800	-17.31856900	-0.95178300
C	0.81622500	-13.08163900	-0.81611200
H	1.17694000	-18.40144900	-0.98147900
C	-18.94487600	-5.02018100	0.88961900
H	-18.78851200	-6.09426300	0.87435600
C	-19.34650200	-2.24527200	0.85887800
C	18.60505900	4.58283200	0.85475700
C	19.92638100	4.02660500	0.82344700
C	18.38766900	5.96804300	0.87137900
H	20.77161700	4.70898500	0.80805100
C	17.47702600	3.70633300	0.87173900
C	17.09989300	6.48474800	0.88120800
H	16.95205600	7.55984900	0.85691500

C	20.12126900	2.68052400	0.81542700
C	19.19720900	0.38180300	0.84027100
H	21.12536300	2.26623100	0.79343200
C	19.01235300	1.77171500	0.83918100
C	18.10926300	-0.47918200	0.84749400
H	18.27319900	-1.55157800	0.81264300
C	17.68188600	2.29107700	0.86498400
H	-1.05138100	-17.31393000	-0.92623800
H	-0.98057100	18.98328100	-0.97065900
H	-5.25510900	18.52481400	-1.02578700
H	19.24270300	6.63805700	0.85819300
H	20.20717300	-0.01781500	0.81826000
H	3.23754400	-17.02128200	-0.99022100
H	-21.08635600	-5.19158700	0.91355100
H	-22.10694400	1.45784500	0.83498900
Ir	6.27043500	-5.25827400	-0.57442000
C	4.99463200	-4.97226900	1.18015800
C	5.97186200	-3.91398800	1.06909000
C	5.58734500	-3.15471700	-0.09450100
C	4.16480500	-3.48527800	-0.37836600
C	3.81045900	-4.57403600	0.37916200
C	2.56026400	-5.38698500	0.30926200
C	3.40197700	-2.87723300	-1.50101000
C	6.23945900	-1.88696000	-0.56349000
C	6.99108800	-3.55633500	2.10458200
C	4.94248200	-5.99420200	2.27759800
H	2.15106700	-5.58403900	1.30685100
H	2.75731700	-6.35998800	-0.15881100
H	1.78856800	-4.88799500	-0.28096000
H	3.53968300	-1.79254300	-1.55336400
H	2.33216500	-3.08554700	-1.42674200
H	3.76282500	-3.31647700	-2.44452800
H	6.12348200	-1.76125100	-1.64365200
H	7.30952700	-1.88190100	-0.34143900
H	5.79514100	-1.00626100	-0.07978500
H	6.48984100	-3.00838100	2.91223600
H	7.77826000	-2.91418200	1.70932400
H	7.46490200	-4.44038800	2.53527700
H	5.94587600	-6.24880300	2.62704300
H	4.46469500	-6.91485200	1.93192600
H	4.36731300	-5.62037000	3.13441400
H	4.88592200	-5.24276200	-2.25414700
O	7.01645200	-5.43553600	-2.68100700
H	7.39088000	-4.56679200	-2.88747700

H	5.86170400	-5.30553100	-2.71892900

TS2’

C	-22.17617200	-1.56144900	0.24701700
C	-21.31299000	0.75718800	0.39946200
H	-23.19087000	-1.17343100	0.23547700
C	-21.09222000	-0.62598300	0.32891400
C	-20.61020900	-3.42395000	0.20592400
C	-21.94539800	-2.90070100	0.18745300
C	-20.35562700	-4.80210700	0.15522400
H	-22.77222200	-3.60286800	0.12733400
C	-1.70892800	14.07662500	-0.33695500
C	-0.74029500	16.31962100	-0.33268300
C	-2.21999500	18.30767300	-0.20463800
H	0.12686300	16.97328800	-0.36888600
H	-13.28564900	3.88202200	-0.01969600
C	-18.65724400	-0.19303400	0.42210600
H	4.58190200	11.55104000	0.09831400
C	-4.48790700	16.64136000	-0.23378100
C	-5.47893900	14.41872600	-0.33250000
C	-4.00918100	12.39372900	-0.35582900
C	-5.14615600	11.44163600	-0.33704700
C	-5.21364900	10.40847200	-1.28735500
C	-6.23145800	9.46474100	-1.25103800
C	-7.24026900	9.53952000	-0.27761300
C	-7.16913500	10.55751400	0.69006600
C	-6.13552000	11.48729300	0.65944600
H	-6.35406500	13.78441500	-0.40026200
H	-4.45550100	10.35509500	-2.06255100
H	-6.28035300	8.66502900	-1.98255400
H	-7.89835200	10.58648000	1.49364800
H	-6.07216600	12.24405100	1.43482400
C	-12.55483300	5.92318200	0.18235900
C	-11.24231300	5.54580200	-0.15405100
C	-10.22407000	6.48530300	-0.20130900
C	-10.49606300	7.83286200	0.08980000
C	-11.80759600	8.20998700	0.42997800
C	-12.82691800	7.27039100	0.47475000
H	-11.03091300	4.50368800	-0.37950300
H	-9.20868900	6.20840700	-0.46166000
H	-12.01899000	9.25190500	0.65625800
H	-13.84270300	7.54845900	0.73265600
C	-13.60654200	4.90721300	0.21598800
N	-14.82312000	5.18372300	0.51241100

C	14.95059700	3.46062200	0.34819200
C	15.77545600	5.77447700	-0.14726200
C	14.42797400	6.38326800	-0.04783400
C	14.20930900	7.49976200	0.77705800
C	12.97168900	8.12855100	0.81992800
C	11.89650700	7.64618600	0.05666300
C	12.11186500	6.53896700	-0.78423300
C	13.35650700	5.92267900	-0.83254400
H	13.96848400	3.85541400	0.57891400
H	15.02453900	7.87024300	1.39089200
H	12.80435200	8.99362000	1.45304300
H	11.31613400	6.19404200	-1.43684000
H	13.51753500	5.09184400	-1.51190600
C	4.53104400	10.47059300	-0.10141300
N	3.42247300	9.88479000	-0.36960000
C	-4.18250200	13.79908100	-0.31526200
C	-5.62465600	15.77077200	-0.28455900
C	-4.62081700	18.03785400	-0.18098100
H	-6.61579700	16.21568700	-0.30103500
H	9.51423500	6.63863400	-0.16714000
C	15.99154600	4.37851100	-0.01952000
H	-9.74215800	9.86977000	0.29795600
C	-2.04062100	16.91639700	-0.25772300
C	-0.58313300	14.96881800	-0.37919800
C	-1.56610600	12.66739400	-0.37347400
C	-0.24601200	11.99156300	-0.36773000
C	0.03417200	10.98477300	-1.30771300
C	1.23870500	10.29429100	-1.28425900
C	2.22407000	10.61060800	-0.33566000
C	1.94340600	11.60004800	0.62325400
C	0.72596200	12.27213000	0.60696900
H	0.40939400	14.54450500	-0.46555200
H	-0.70628000	10.75124500	-2.06658100
H	1.45224900	9.51605700	-2.00938600
H	2.66450600	11.80497700	1.40834000
H	0.50915600	13.00637400	1.37623500
C	8.28646800	8.43429500	-0.02396200
C	7.09684400	7.71751000	-0.24610800
C	5.87159800	8.36676600	-0.27084800
C	5.80696400	9.75570400	-0.06787300
C	6.99562800	10.47096900	0.16379200
C	8.22117400	9.82289000	0.18242500
H	7.14580300	6.64326100	-0.40433100
H	4.94824300	7.82670500	-0.44807100

H	6.94659200	11.54512900	0.32304700
H	9.14578700	10.36295000	0.35235300
C	9.56795100	7.72739800	-0.02358000
N	10.68208100	8.34069200	0.13962800
C	-17.33384400	-0.73895800	0.53139100
C	-18.91775500	1.20107200	0.45150900
C	-17.82983200	2.20726700	0.46439000
C	-17.83501800	3.25465400	1.40138500
C	-16.84636600	4.23059200	1.39290900
C	-15.79744900	4.17701900	0.46136100
C	-15.80144900	3.14902100	-0.49870500
C	-16.80278100	2.18532300	-0.49450600
H	-16.50453400	-0.06026200	0.68936800
H	-18.62196400	3.29090500	2.14845500
H	-16.85032100	5.03763500	2.11799700
H	-15.04257300	3.13579600	-1.27464600
H	-16.81032300	1.41593800	-1.25991700
C	-9.43924600	8.84422800	0.04103900
N	-8.23741500	8.55544000	-0.29908900
C	-3.49742300	18.86069900	-0.16276600
C	-3.02434300	14.64011700	-0.30087700
H	-3.61819400	19.93899300	-0.12192900
C	-2.71404900	11.87087100	-0.39761300
C	-3.18370600	16.06092200	-0.26146600
H	-2.59322400	10.79252700	-0.38887800
C	-19.74921500	-1.11245400	0.34194200
C	-20.24772500	1.64520800	0.45697600
H	-20.43845300	2.71372900	0.47475200
C	2.32923100	-14.95913400	-0.71616500
C	3.37735000	-12.76896900	-0.52367600
C	1.95907400	-10.70853400	-0.44376500
C	3.12508200	-9.81315600	-0.24613200
C	3.35594400	-8.73514100	-1.11619200
C	4.42853000	-7.87024700	-0.92601900
C	5.30828100	-8.06742100	0.14462600
C	5.07378100	-9.12326200	1.03781400
C	3.99841700	-9.98264400	0.83999600
H	4.27072700	-12.15918000	-0.46846900
H	2.69292600	-8.58897700	-1.96314300
H	4.60516800	-7.04689900	-1.60744700
H	5.71355600	-9.24250800	1.90620600
H	3.81334800	-10.78206100	1.54992400
C	10.52935200	-4.72548000	1.12714300
C	9.18946900	-4.38233800	0.89153700

C	8.20882600	-5.36228900	0.78064400
C	8.60832700	-6.71303700	0.94797000
C	9.94252200	-7.06341000	1.24706600
C	10.90132600	-6.07145700	1.33556100
H	8.95735600	-3.34850100	0.67090200
H	10.21576900	-8.10841000	1.37123900
H	11.94453200	-6.29934500	1.52234600
C	11.52120900	-3.65398100	1.04206400
N	12.77674300	-3.87105800	1.19796700
C	-17.10278700	-2.07954800	0.47686400
C	-17.94857100	-4.42447800	0.21741900
C	-16.58922700	-5.01421500	0.18183300
C	-16.25907100	-6.08614000	1.02907900
C	-15.00955900	-6.68912400	0.97088100
C	-14.02977100	-6.21964200	0.08160000
C	-14.35860600	-5.16249900	-0.78579700
C	-15.61837800	-4.57644800	-0.73548100
H	-16.09232700	-2.45191900	0.59338100
H	-16.99532600	-6.43974400	1.74421300
H	-14.75681600	-7.51791600	1.62378900
H	-13.64023000	-4.83532700	-1.53096100
H	-15.86982900	-3.78562200	-1.43486000
C	-6.58165100	-8.77114900	-0.40208600
N	-5.48292000	-8.14159100	-0.60604300
C	-0.37391300	-12.31323300	-0.72814200
C	-1.39578900	-14.51505400	-1.00524900
C	0.02463200	-16.55016400	-0.99427400
H	-2.27497500	-15.13510800	-1.15687500
H	11.11821800	-2.66658900	0.78774400
C	16.46510900	1.54780800	0.18306600
H	-6.60487200	-9.86216500	-0.26535500
C	-0.11741200	-15.15584800	-0.91647800
C	-1.51731500	-13.16236200	-0.92291100
C	-0.48092800	-10.90336900	-0.63218600
C	-1.79098300	-10.20801100	-0.61853500
C	-2.04259700	-9.13517700	-1.48990400
C	-3.25480300	-8.45588800	-1.46098000
C	-4.27117400	-8.84689300	-0.57495000
C	-4.01249600	-9.89560000	0.32596900
C	-2.79179700	-10.55952600	0.30246100
H	-2.49246200	-12.70185700	-1.02019800
H	-1.27834200	-8.84479000	-2.20450400
H	-3.45039500	-7.63142900	-2.13854000
H	-4.75705000	-10.15905200	1.07044800

H	-2.59673200	-11.34951900	1.02072300
C	-10.40646100	-6.87651200	-0.22595600
C	-9.24923700	-6.11035400	-0.45450600
C	-8.00039800	-6.71147700	-0.51479800
C	-7.88086300	-8.10172700	-0.34989200
C	-9.03881800	-8.86794200	-0.12475500
C	-10.28642300	-8.26705200	-0.06279100
H	-9.34081900	-5.03477500	-0.58173800
H	-7.10082900	-6.13136800	-0.68807500
H	-8.94723900	-9.94358600	0.00200200
H	-11.18639200	-8.84564600	0.11231800
C	-11.71266600	-6.21881300	-0.15791200
N	-12.78985700	-6.87228200	0.07923300
C	15.17557100	2.12126300	0.44707900
C	16.71545200	0.15301400	0.25516200
C	15.64682800	-0.84206200	0.50963100
C	15.83563000	-1.84021800	1.48143400
C	14.88208900	-2.82736400	1.68970200
C	13.68769900	-2.83452600	0.95216100
C	13.49344600	-1.84761400	-0.03328000
C	14.46232600	-0.87363300	-0.24760800
H	14.37029300	1.46605400	0.75587500
H	16.74220200	-1.83087000	2.07880500
H	15.03097000	-3.60209200	2.43472500
H	12.60259600	-1.87461400	-0.65424900
H	14.31795000	-0.14254600	-1.03663300
C	7.56696500	-7.65800000	0.69876300
N	6.40098400	-7.18857800	0.33249100
C	2.09727900	-12.11536900	-0.53002100
C	3.48718000	-14.12278600	-0.60459700
C	2.42506600	-16.35718900	-0.79760200
H	4.46630900	-14.59362300	-0.60161800
H	-11.71129200	-5.12909700	-0.30589400
C	-18.16870000	-3.02575600	0.30401600
H	7.72200600	-8.73427300	0.76872500
C	0.68462700	-10.14076700	-0.51569000
C	1.04399000	-14.33983300	-0.76563400
H	0.58972400	-9.06415700	-0.41328400
C	1.28323600	-17.14310100	-0.93193000
C	0.92167000	-12.91816800	-0.67226600
H	1.37516600	-18.22312100	-0.99384800
C	-19.05455200	-5.28501800	0.15935900
H	-18.87787200	-6.35352900	0.08572700
C	-19.50641500	-2.52043400	0.28136700

C	18.39892200	4.73181400	-0.47595700
C	19.71229200	4.18643900	-0.66031500
C	18.15419700	6.11037100	-0.55505300
H	20.52884100	4.87130000	-0.87157300
C	17.30801400	3.85080800	-0.20190400
C	16.87094400	6.61406000	-0.39565700
H	16.69520800	7.68046300	-0.49748200
C	19.93482900	2.84745700	-0.57214100
C	19.08053400	0.55425500	-0.18350600
H	20.93292800	2.44149400	-0.71175000
C	18.86496600	1.93567400	-0.28903700
C	18.02820400	-0.31050100	0.08062600
H	18.21025900	-1.37951600	0.12674300
C	17.54259800	2.44348100	-0.10511000
H	-0.86434400	-17.16404700	-1.10799000
H	-1.34462300	18.95103800	-0.20069000
H	-5.61691000	18.47089600	-0.15851200
H	18.98131000	6.78358600	-0.76228800
H	20.08389600	0.16278000	-0.32638100
H	3.40676900	-16.82061000	-0.75805300
H	-21.19198000	-5.49288400	0.09609400
H	-22.33291100	1.13115500	0.39044900
Ir	6.27338700	-5.06755600	0.24351900
C	4.53665800	-4.76866200	1.49778100
C	5.62770100	-3.86499400	1.87980700
C	5.83991800	-2.95157100	0.78370300
C	4.88311200	-3.29645200	-0.24595000
C	4.04705800	-4.36744300	0.20161800
C	2.85236600	-4.92757600	-0.50417800
C	4.82407300	-2.60947600	-1.56810000
C	6.74759200	-1.76372000	0.75789000
C	6.30063400	-3.81657200	3.21368100
C	3.92972200	-5.81488800	2.37529900
H	2.67984900	-5.97062500	-0.23282800
H	2.97123600	-4.88275800	-1.58934600
H	1.94845800	-4.36156400	-0.24605600
H	4.58793000	-1.54887900	-1.42298700
H	4.06619000	-3.04626400	-2.22068700
H	5.80691000	-2.68560800	-2.04544200
H	7.46623800	-1.81639300	-0.06768800
H	7.31425900	-1.69016900	1.68904300
H	6.15792400	-0.84422200	0.65864400
H	5.77540600	-3.12028300	3.87849500
H	7.33561800	-3.48547600	3.11561600

H	6.30880100	-4.80000800	3.68807800
H	4.67218100	-6.23845600	3.05475100
H	3.50591100	-6.63125300	1.78801600
H	3.12706800	-5.37682000	2.98077200
H	6.59234700	-6.07059000	-1.93146600
O	7.67129200	-3.73405900	-1.86509100
H	6.99045300	-5.42952100	-2.08186600
C	8.77205200	-3.09848200	-2.00114500
O	9.27197700	-2.25934000	-1.22881500
H	9.33473300	-3.33849400	-2.93622800

IM-4 + H_2

C	-22.02176400	-1.51245200	0.22839500
C	-21.16339000	0.81129100	0.31449300
H	-23.03691200	-1.12673100	0.19667600
C	-20.93986800	-0.57297800	0.28892700
C	-20.45274000	-3.37254500	0.25907300
C	-21.78852000	-2.85250700	0.21365100
C	-20.19571000	-4.75114900	0.25500600
H	-22.61379400	-3.55771100	0.16982800
C	-1.59207100	14.17896100	-0.37507000
C	-0.62770700	16.42340000	-0.34262600
C	-2.11354500	18.40844100	-0.24112900
H	0.23880900	17.07875000	-0.35772900
H	-13.13841600	3.94836700	-0.12999200
C	-18.50622300	-0.13302500	0.38749600
H	4.69055200	11.64982900	0.24900900
C	-4.37730100	16.73804800	-0.32102400
C	-5.36189300	14.51369300	-0.44413900
C	-3.88819800	12.49146100	-0.44240500
C	-5.02348900	11.53727100	-0.44022700
C	-5.07439900	10.50072800	-1.38790300
C	-6.08968300	9.55383600	-1.36269000
C	-7.11216500	9.62855300	-0.40357600
C	-7.05831200	10.65072300	0.56081100
C	-6.02743900	11.58373100	0.54148800
H	-6.23422300	13.87777900	-0.53043600
H	-4.30501000	10.44673100	-2.15192800
H	-6.12532600	8.75120700	-2.09177400
H	-7.79884200	10.67997100	1.35394800
H	-5.97752400	12.34336800	1.31502500
C	-12.41777400	5.99651900	0.03850700
C	-11.10054600	5.61944100	-0.27928400
C	-10.08563500	6.56240300	-0.33111000

C	-10.36593800	7.91308900	-0.06336900
C	-11.68255500	8.29017300	0.25662800
C	-12.69842400	7.34717800	0.30588500
H	-10.88250600	4.57472800	-0.48550800
H	-9.06643400	6.28542300	-0.57603200
H	-11.90034700	9.33457400	0.46469300
H	-13.71782600	7.62480400	0.54954400
C	-13.46545000	4.97663000	0.08254600
N	-14.68514000	5.25445400	0.36446400
C	15.04961500	3.52402100	0.42788300
C	15.88694800	5.87789200	0.23044600
C	14.53250100	6.47459100	0.30600200
C	14.25944600	7.50630000	1.22063100
C	13.02120000	8.13440100	1.24555800
C	11.99757100	7.73364500	0.37205600
C	12.26683200	6.71080600	-0.55547600
C	13.51346900	6.09667200	-0.58517200
H	14.05260800	3.89242600	0.63769100
H	15.03333900	7.81194700	1.91796700
H	12.81356300	8.93547200	1.94719300
H	11.51376100	6.43596600	-1.28730000
H	13.71918100	5.33635400	-1.33180700
C	4.64703000	10.57816100	0.00562300
N	3.54991100	10.00296000	-0.32503100
C	-4.06481100	13.89647300	-0.40272600
C	-5.51110900	15.86537500	-0.39652500
C	-4.51387600	18.13415900	-0.26759400
H	-6.50258000	16.30842600	-0.43196800
H	9.63491600	6.75148500	-0.05201900
C	16.10480800	4.47636300	0.22523600
H	-9.62229300	9.95490800	0.12956000
C	-1.93048700	17.01764300	-0.29402300
C	-0.46710100	15.07301700	-0.38987400
C	-1.44614800	12.77030900	-0.41470300
C	-0.12450600	12.09798700	-0.38564200
C	0.18253100	11.10491300	-1.33144900
C	1.38911800	10.41881600	-1.28686100
C	2.34871300	10.72508800	-0.30906700
C	2.03976800	11.69993700	0.65632000
C	0.82136000	12.36888500	0.61719500
H	0.52783000	14.65047600	-0.45543100
H	-0.53822800	10.87860500	-2.11115500
H	1.62420900	9.65119700	-2.01661200
H	2.73852000	11.89616200	1.46349300

H	0.58218900	13.09261600	1.38981300
C	8.39717500	8.53527800	0.14730400
C	7.22015100	7.83365500	-0.16985900
C	5.99653200	8.48475400	-0.21540900
C	5.92116100	9.86089600	0.05875700
C	7.09712200	10.56097900	0.38302100
C	8.32092700	9.91070200	0.42542200
H	7.27754200	6.76955900	-0.38420100
H	5.08268800	7.95661700	-0.46354700
H	7.03977600	11.62519300	0.59672100
H	9.23572700	10.43951100	0.66824200
C	9.67783100	7.82768500	0.17023400
N	10.78039400	8.42436400	0.43887200
C	-17.18270500	-0.67296800	0.52249200
C	-18.76934000	1.26080500	0.37301900
C	-17.68363800	2.26951700	0.36744600
C	-17.69977100	3.34152600	1.27599700
C	-16.71347800	4.31945700	1.25014900
C	-15.65614200	4.24386900	0.32973500
C	-15.64897900	3.19060700	-0.60253300
C	-16.64796200	2.22462300	-0.58135400
H	-16.35544400	0.01171000	0.66460500
H	-18.49346800	3.39554100	2.01479200
H	-16.72568800	5.14558100	1.95332400
H	-14.88307700	3.15835100	-1.37098600
H	-16.64666900	1.43488100	-1.32578900
C	-9.31235800	8.92765200	-0.11178800
N	-8.10474100	8.64010800	-0.43201000
C	-3.39262100	18.95899900	-0.22433600
C	-2.90894100	14.73982800	-0.36379900
H	-3.51624900	20.03695900	-0.18333000
C	-2.59138500	11.97121600	-0.46294200
C	-3.07170000	16.16017300	-0.32372500
H	-2.46829400	10.89309800	-0.45522600
C	-19.59622400	-1.05639800	0.32757000
C	-20.09999500	1.70250300	0.35374800
H	-20.29246200	2.77074400	0.33773300
C	2.34124100	-15.12158500	-0.56887900
C	3.43706800	-12.94828100	-0.47712600
C	2.06404600	-10.85787000	-0.40709800
C	3.25484800	-9.98087000	-0.28153900
C	3.47583800	-8.94262300	-1.20395600
C	4.56948600	-8.09331700	-1.08604600
C	5.47508600	-8.28265200	-0.03519100

C	5.26801900	-9.29883200	0.90247100
C	4.16131500	-10.13655300	0.77794300
H	4.34237900	-12.35399600	-0.46962700
H	2.78453600	-8.81739800	-2.03170100
H	4.76659200	-7.29265000	-1.79149500
H	5.95668000	-9.40811100	1.73450000
H	3.98582500	-10.91036500	1.51820700
C	10.68746300	-4.79561500	0.16838900
C	9.31479600	-4.50896300	0.11048300
C	8.34638400	-5.52204400	0.12415000
C	8.83653500	-6.86107300	0.14402600
C	10.21171000	-7.16007600	0.18953300
C	11.13558400	-6.13380200	0.21466300
H	9.00701400	-3.46810900	0.04885600
H	10.54016000	-8.19658200	0.20162600
H	12.20176300	-6.32206000	0.25808300
C	11.64978600	-3.69246200	0.17811400
N	12.91072000	-3.89135400	0.30054000
C	-16.94924800	-2.01422500	0.51122600
C	-17.78968800	-4.36751300	0.32160000
C	-16.42941300	-4.95620500	0.31243300
C	-16.10242000	-5.99953900	1.19581000
C	-14.85339900	-6.60527000	1.16082700
C	-13.87106700	-6.16742100	0.25820400
C	-14.19624000	-5.13806500	-0.64338800
C	-15.45528500	-4.54886900	-0.61525600
H	-15.93900600	-2.38126300	0.64542900
H	-16.84113300	-6.32907100	1.91988000
H	-14.60346200	-7.41279100	1.84091100
H	-13.47577200	-4.83646300	-1.39725700
H	-15.70389800	-3.78092100	-1.34070400
C	-6.44321200	-8.78235900	-0.19584800
N	-5.34332000	-8.17015300	-0.44144300
C	-0.30924800	-12.42275400	-0.56569800
C	-1.38227100	-14.61075100	-0.74532900
C	-0.00186200	-16.67329800	-0.72221100
H	-2.27796200	-15.21684200	-0.84994900
H	11.22593900	-2.68066000	0.09154500
C	16.58522700	1.63975700	0.15797500
H	-6.47327100	-9.86780600	-0.02156200
C	-0.11430000	-15.27469400	-0.68032300
C	-1.47453300	-13.25406000	-0.69845900
C	-0.38458700	-11.00876800	-0.50692300
C	-1.68005300	-10.28721700	-0.47528100

C	-1.93639000	-9.23515600	-1.37034300
C	-3.13726700	-8.53639300	-1.33205700
C	-4.13808400	-8.88739000	-0.41240100
C	-3.87247400	-9.91194300	0.51384100
C	-2.66237100	-10.59454000	0.48092900
H	-2.44348900	-12.77722500	-0.77743500
H	-1.18547000	-8.97742100	-2.11102500
H	-3.33704700	-7.72904200	-2.02873200
H	-4.60255000	-10.14208000	1.28333900
H	-2.46107900	-11.36580700	1.21754400
C	-10.25206600	-6.85331100	-0.03773500
C	-9.09183800	-6.10357000	-0.30266700
C	-7.84786900	-6.71552800	-0.35584800
C	-7.73625300	-8.10038900	-0.14624900
C	-8.89696300	-8.84979400	0.11787600
C	-10.13968900	-8.23824700	0.17201800
H	-9.17727100	-5.03225500	-0.46544300
H	-6.94617900	-6.14842000	-0.55866900
H	-8.81141400	-9.92121500	0.27972700
H	-11.04178700	-8.80427700	0.37505200
C	-11.55377800	-6.18578400	0.01544000
N	-12.63409500	-6.82500400	0.27639500
C	15.27759100	2.18174400	0.39887400
C	16.84118100	0.24564100	0.09621600
C	15.76732900	-0.77370200	0.16826400
C	15.90426300	-1.87797800	1.02817100
C	14.95633800	-2.89123600	1.05390400
C	13.81313600	-2.82291600	0.24101400
C	13.67075600	-1.72622600	-0.62775900
C	14.63605700	-0.72489300	-0.66396500
H	14.45969900	1.49696800	0.58733600
H	16.76896500	-1.93310700	1.68227800
H	15.06957700	-3.74748300	1.71053100
H	12.82851300	-1.69081300	-1.31179800
H	14.53371800	0.09151500	-1.37175000
C	7.80339800	-7.85220900	0.08952200
N	6.57987300	-7.40552600	0.06959500
C	2.17035900	-12.26922800	-0.45806700
C	3.51820000	-14.30571300	-0.52197700
C	2.40719800	-16.52307900	-0.61254800
H	4.48765900	-14.79599500	-0.53899900
H	-11.54670000	-5.10142000	-0.16792800
C	-18.01263400	-2.96718400	0.36165400
H	8.00146400	-8.92225800	0.03504800

C	0.79893800	-10.26697800	-0.45092600
C	1.06704300	-14.47812700	-0.59243200
H	0.73000200	-9.18607800	-0.37747800
C	1.24655700	-17.28937500	-0.68506700
C	0.97543000	-13.05204400	-0.53612400
H	1.31563300	-18.37244100	-0.71847800
C	-18.89395600	-5.23139000	0.28393200
H	-18.71504500	-6.30138600	0.24612000
C	-19.35089100	-2.46515600	0.31344900
C	18.53385700	4.87649900	-0.03589400
C	19.86038300	4.35461300	-0.19118500
C	18.28467300	6.25554800	0.01383200
H	20.68361700	5.05847900	-0.27584200
C	17.43399100	3.97122100	0.07338500
C	16.99034600	6.73911200	0.14165900
H	16.81405900	7.81019900	0.14015300
C	20.08685000	3.01405300	-0.23024600
C	19.22639000	0.69249400	-0.14308900
H	21.09474300	2.62560900	-0.34690700
C	19.00755900	2.07709000	-0.11477100
C	18.16576800	-0.19602300	-0.04182700
H	18.35288000	-1.26358700	-0.09947200
C	17.67232700	2.56169500	0.03628800
H	-0.90593500	-17.27204000	-0.78815000
H	-1.23968100	19.05342600	-0.21727600
H	-5.51101600	18.56534300	-0.26437900
H	19.11837900	6.94720800	-0.06818000
H	20.23966100	0.31901900	-0.26156000
H	3.38071400	-17.00480500	-0.59284900
H	-21.03057100	-5.44495400	0.21238300
H	-22.18378700	1.18296100	0.28563900
Ir	6.33897300	-5.34033400	0.08345700
C	5.15956800	-4.95750600	1.93149900
C	6.07597700	-3.87500000	1.69954900
C	5.72447100	-3.26491100	0.43119200
C	4.50749700	-3.91539300	-0.03852200
C	4.15872100	-4.94255800	0.86699900
C	2.97436800	-5.85518400	0.79851000
C	3.87644900	-3.62402800	-1.36155900
C	6.32234400	-2.03005200	-0.16820700
C	7.15123900	-3.41283400	2.63122400
C	5.11205100	-5.85119200	3.13238100
H	3.22571000	-6.86290400	1.13701300
H	2.59301400	-5.94181000	-0.22021200

H	2.16197200	-5.48296800	1.43537800
H	3.71503600	-2.54933300	-1.49222400
H	2.91544500	-4.13073400	-1.46974700
H	4.55076500	-3.98099100	-2.15119700
H	6.31987000	-2.08770400	-1.25948400
H	7.35689000	-1.89354900	0.15669000
H	5.76382200	-1.13169300	0.12515200
H	6.77113200	-2.62352800	3.29087200
H	8.00831700	-3.01187600	2.08712900
H	7.51424800	-4.23233500	3.25494000
H	6.09099000	-5.92056200	3.61134900
H	4.80351500	-6.86244500	2.85394200
H	4.39669000	-5.47619500	3.87504100
H	5.05155700	-6.24007700	-4.61357500
O	6.33985800	-5.47836900	-1.97715900
H	7.23509200	-5.30409100	-2.29461600
H	5.36182000	-6.04632200	-3.96047100

IM-2 + H_2

C	-22.11246400	-1.44428800	0.56462700
C	-21.23746300	0.87413700	0.62296400
H	-23.12561400	-1.05214900	0.55657400
C	-21.02348400	-0.51163900	0.59658500
C	-20.55500300	-3.31431700	0.56095800
C	-21.88816400	-2.78584700	0.54706300
C	-20.30685700	-4.69454600	0.55200300
H	-22.71871800	-3.48583500	0.52461900
C	-1.62218300	14.13303700	-0.62601500
C	-0.65593100	16.37634400	-0.67536800
C	-2.14013200	18.36502800	-0.64460800
H	0.21110500	17.02998600	-0.71485800
H	-13.20440400	3.95469600	-0.01680300
C	-18.58547300	-0.08700900	0.63562900
H	4.65653800	11.63192500	0.10467900
C	-4.40530700	16.69469100	-0.66097100
C	-5.39172000	14.46807300	-0.70159000
C	-3.91938000	12.44613100	-0.62995800
C	-5.05625200	11.49457800	-0.59058100
C	-5.11483600	10.42738100	-1.50298000
C	-6.13342400	9.48554300	-1.44175400
C	-7.15099400	9.59538400	-0.48079800
C	-7.08849400	10.64817200	0.44962200
C	-6.05497200	11.57665500	0.39418100
H	-6.26465500	13.83008300	-0.76301200

H	-4.34922300	10.34548000	-2.26832400
H	-6.17560400	8.65957900	-2.14393000
H	-7.82455000	10.70614100	1.24532700
H	-5.99878300	12.36160800	1.14158200
C	-12.46884300	6.00099000	0.10596000
C	-11.15832100	5.61029500	-0.22294600
C	-10.13928700	6.54637600	-0.30836800
C	-10.40882200	7.90388300	-0.06513500
C	-11.71885800	8.29467300	0.26533700
C	-12.73863100	7.35838600	0.34927400
H	-10.94873700	4.56040700	-0.41087300
H	-9.12508600	6.25884800	-0.56174700
H	-11.92836700	9.34439800	0.45421300
H	-13.75294800	7.64657700	0.60174000
C	-13.52128600	4.98818200	0.18568600
N	-14.73389200	5.27750600	0.48602800
C	14.99594600	3.52014700	0.66784500
C	15.85102800	5.86590800	0.44973800
C	14.49776400	6.46957200	0.47377200
C	14.20368600	7.52101500	1.35898400
C	12.96667200	8.15169800	1.33882700
C	11.96466000	7.73363400	0.44856500
C	12.25567500	6.69202100	-0.45099300
C	13.50156100	6.07574600	-0.43617300
H	13.99477600	3.89679300	0.83945500
H	14.95977200	7.83936600	2.07004100
H	12.74264600	8.96726300	2.01838600
H	11.52066100	6.40275900	-1.19550100
H	13.72449300	5.29971700	-1.16135400
C	4.61611800	10.55308600	-0.10537800
N	3.52139800	9.96601100	-0.42310200
C	-4.09496400	13.85178100	-0.64030300
C	-5.53987600	15.82071300	-0.70283100
C	-4.54074000	18.09193100	-0.65810600
H	-6.53109100	16.26297200	-0.75255800
H	9.61310300	6.73834200	-0.00079700
C	16.06179800	4.46353000	0.47954400
H	-9.65472200	9.94630900	0.06767400
C	-1.95819900	16.97308000	-0.64719300
C	-0.49644400	15.02500600	-0.67381400
C	-1.47729900	12.72376500	-0.61529600
C	-0.15557600	12.05303300	-0.56169700
C	0.15454300	11.02943700	-1.47323600
C	1.36213600	10.34701000	-1.40349500

C	2.31920000	10.68660800	-0.43422200
C	2.00657600	11.69226100	0.49790300
C	0.78768500	12.35823900	0.43367100
H	0.49807600	14.59945300	-0.72458400
H	-0.56435700	10.77630300	-2.24641300
H	1.59988900	9.55603500	-2.10697100
H	2.70300200	11.91588100	1.29992800
H	0.54597400	13.10726100	1.18100500
C	8.36860900	8.52384300	0.13358500
C	7.19723900	7.81119500	-0.17995500
C	5.97281900	8.45785000	-0.25769800
C	5.89117500	9.84065500	-0.02144200
C	7.06182800	10.55228900	0.29697100
C	8.28621700	9.90623200	0.37266000
H	7.25945900	6.74186200	-0.36473400
H	5.06318500	7.92073800	-0.50201800
H	6.99966000	11.62180800	0.48081500
H	9.19678500	10.44341900	0.61298300
C	9.64991800	7.81959700	0.19629900
N	10.74601000	8.42470400	0.47260100
C	-17.26277200	-0.63519600	0.74219500
C	-18.83982400	1.30849200	0.62161600
C	-17.74826600	2.31012600	0.58386500
C	-17.73768300	3.38994300	1.48331400
C	-16.74689200	4.36198800	1.42671100
C	-15.71092500	4.27221500	0.48351900
C	-15.73038200	3.21071300	-0.43910200
C	-16.73433900	2.25102100	-0.38739200
H	-16.42819000	0.04440600	0.86445200
H	-18.51413700	3.45493700	2.23934400
H	-16.73886100	5.19427900	2.12263800
H	-14.98185800	3.16751100	-1.22397600
H	-16.75437200	1.45521600	-1.12505000
C	-9.35122400	8.91185100	-0.14979700
N	-8.14781900	8.61067100	-0.47346400
C	-3.41877400	18.91686600	-0.64630100
C	-2.93850900	14.69509900	-0.63352800
H	-3.54151000	19.99570800	-0.64434000
C	-2.62308500	11.92417100	-0.63407700
C	-3.10013900	16.11607800	-0.64453000
H	-2.50075300	10.84687700	-0.58840900
C	-19.68241300	-1.00355500	0.60441200
C	-20.16780200	1.75860500	0.63282100
H	-20.35392200	2.82796500	0.61682300

C	2.26209700	-15.01015900	-0.97646600
C	3.33848100	-12.83362600	-0.78533500
C	1.94605600	-10.76236300	-0.60071200
C	3.12715400	-9.88049200	-0.43100400
C	3.32374900	-8.77928800	-1.28267500
C	4.40600600	-7.92279400	-1.11795200
C	5.32843200	-8.16393400	-0.09255100
C	5.14125200	-9.24480400	0.77699800
C	4.04803600	-10.09033300	0.60698400
H	4.23878000	-12.23245300	-0.75604300
H	2.62238000	-8.60922300	-2.09365100
H	4.57794300	-7.07961700	-1.77803100
H	5.83430000	-9.39659500	1.59851500
H	3.89140500	-10.91180500	1.29836700
C	10.57008300	-4.73705600	0.38375700
C	9.20746400	-4.42421900	0.26202000
C	8.23048800	-5.42396900	0.23735100
C	8.68897600	-6.76855600	0.27948600
C	10.05535100	-7.09406900	0.39164300
C	10.99285200	-6.08249100	0.45887700
H	8.91545800	-3.38416900	0.15907000
H	10.36519600	-8.13597200	0.41813100
H	12.05280600	-6.28788000	0.55116200
C	11.54967200	-3.64941500	0.41650800
N	12.80116700	-3.86612300	0.59464900
C	-17.03816900	-1.97793100	0.72850000
C	-17.89750900	-4.32619500	0.55930300
C	-16.54153800	-4.92302100	0.51567300
C	-16.19972500	-5.97249700	1.38620200
C	-14.95503700	-6.58455800	1.31971100
C	-13.99120500	-6.14631900	0.39760900
C	-14.33163600	-5.11120500	-0.49155400
C	-15.58695900	-4.51647900	-0.43245600
H	-16.02750600	-2.35099200	0.84103700
H	-16.92313500	-6.30163000	2.12573100
H	-14.69391200	-7.39654000	1.99021800
H	-13.62656100	-4.80954500	-1.25982400
H	-15.84823300	-3.74418300	-1.14873000
C	-6.57351400	-8.75378700	-0.23928000
N	-5.47755500	-8.13191600	-0.47818800
C	-0.41311700	-12.34026100	-0.82594000
C	-1.46710400	-14.52694700	-1.10384000
C	-0.06752200	-16.57469500	-1.19008300
H	-2.35781500	-15.13594400	-1.23136300

H	11.14567200	-2.63423600	0.29146900
C	16.52936300	1.62413000	0.48193700
H	-6.60071400	-9.84432500	-0.09980000
C	-0.19254800	-15.18092100	-1.07954000
C	-1.57166100	-13.17504700	-0.99096000
C	-0.50154600	-10.93162400	-0.69921200
C	-1.80318800	-10.22385600	-0.62912600
C	-2.07334600	-9.13715500	-1.47736700
C	-3.27858700	-8.44930300	-1.40094400
C	-4.26972400	-8.84484900	-0.48885300
C	-3.99051700	-9.90628300	0.39080100
C	-2.77650100	-10.57905100	0.31960700
H	-2.54542700	-12.70400500	-1.03991300
H	-1.32954500	-8.84299300	-2.21165500
H	-3.48926700	-7.61449800	-2.06108400
H	-4.71274200	-10.17391400	1.15554300
H	-2.56468900	-11.37986200	1.02094900
C	-10.37966200	-6.83141800	0.02501100
C	-9.22364400	-6.07510600	-0.23956000
C	-7.98068800	-6.68507900	-0.32601100
C	-7.86602200	-8.07468000	-0.15231400
C	-9.02258600	-8.83080200	0.11081600
C	-10.26427200	-8.22096400	0.19947700
H	-9.31162100	-5.00025200	-0.37531600
H	-7.08212400	-6.11296000	-0.52852300
H	-8.93477800	-9.90591000	0.24459200
H	-11.16317200	-8.79205300	0.40258700
C	-11.67999000	-6.16494100	0.11359500
N	-12.75596400	-6.80748100	0.38402800
C	15.21764300	2.17656400	0.67113100
C	16.77940200	0.22783900	0.45392600
C	15.69638100	-0.78286100	0.50563300
C	15.79181700	-1.87210000	1.38978300
C	14.83252600	-2.87518700	1.40059700
C	13.71957600	-2.81032000	0.54660800
C	13.62034600	-1.72992800	-0.34838700
C	14.59659900	-0.73921500	-0.36819400
H	14.39056300	1.49932600	0.84612800
H	16.63210600	-1.92312200	2.07534200
H	14.91295400	-3.71962400	2.07710500
H	12.80368600	-1.69933600	-1.06287700
H	14.52797600	0.06474400	-1.09403600
C	7.64450800	-7.74075300	0.15273100
N	6.42662100	-7.28726600	0.06022400

C	2.06582200	-12.16836000	-0.72363900
C	3.43173800	-14.18638200	-0.89783800
C	2.34072100	-16.40715700	-1.08887800
H	4.40554500	-14.66578500	-0.94628700
H	-11.67589800	-5.07769700	-0.05188700
C	-18.11061800	-2.92440700	0.60437500
H	7.83633900	-8.81218000	0.09552700
C	0.67501000	-10.18232600	-0.61101700
C	0.98200400	-14.37841300	-0.96045800
H	0.59523100	-9.10723700	-0.48322600
C	1.18670300	-17.18001200	-1.19103900
C	0.87765200	-12.95774500	-0.83390800
H	1.26545100	-18.25943900	-1.27754800
C	-19.00787700	-5.18303100	0.54907200
H	-18.83686600	-6.25415600	0.50693600
C	-19.44643100	-2.41390400	0.58749200
C	18.49994200	4.84728400	0.28695200
C	19.82799400	4.31615500	0.18484700
C	18.25633200	6.22818400	0.30030600
H	20.65719700	5.01435900	0.11220900
C	17.39250700	3.94944000	0.37963900
C	16.96111500	6.72007600	0.37755700
H	16.79050900	7.79170500	0.34865000
C	20.04864900	2.97402600	0.18043400
C	19.17349900	0.65849200	0.28597600
H	21.05777000	2.57876200	0.10408700
C	18.96136900	2.04446000	0.27980800
C	18.10527800	-0.22295000	0.36885000
H	18.28818000	-1.29236200	0.33746700
C	17.62462600	2.53836900	0.37802700
H	-0.96646400	-17.17811700	-1.27910900
H	-1.26574900	19.00975100	-0.64518800
H	-5.53753900	18.52377800	-0.66921200
H	19.09572500	6.91428000	0.23026600
H	20.18812600	0.27801700	0.20823600
H	3.31871400	-16.87998000	-1.09908300
H	-21.14688700	-5.38305700	0.53029200
H	-22.25586700	1.25229200	0.61742300
Ir	6.22359700	-5.19951500	0.10465100
C	4.89633800	-4.96443100	1.84366100
C	5.93018200	-3.96100800	1.88469300
C	5.77301600	-3.12228900	0.71346900
C	4.58971100	-3.58430300	0.01231800
C	4.02855800	-4.68936800	0.70700300

C	2.76181300	-5.41636500	0.38303300
C	4.07839200	-2.97536800	-1.25403800
C	6.53514600	-1.87468400	0.39448200
C	6.94404300	-3.77064800	2.96851500
C	4.65142700	-6.03059800	2.86587900
H	2.83016300	-6.47626200	0.63671700
H	2.52568300	-5.35044800	-0.68155900
H	1.91513500	-4.99323300	0.93867200
H	3.45231400	-2.10381800	-1.02645800
H	3.47408700	-3.68492000	-1.82332800
H	4.91119900	-2.65058200	-1.87920300
H	6.81590600	-1.86610400	-0.65981900
H	7.44848900	-1.81390500	0.99122000
H	5.93616800	-0.98339300	0.61970600
H	6.60165700	-3.01481500	3.68524700
H	7.90274200	-3.44370700	2.56083700
H	7.11948800	-4.70034600	3.51369600
H	5.56865800	-6.28107900	3.40290600
H	4.27592400	-6.94465300	2.39937500
H	3.90808400	-5.69763700	3.60017400
O	6.46664200	-5.45794700	-1.98108200
C	6.93066600	-4.48089000	-2.70043700
O	7.23772800	-3.34698700	-2.34774300
H	7.02682700	-4.78642900	-3.76283100

CO_2

O	0.000000	-0.000000	1.169174
C	0.000000	0.000000	0.000000
O	0.000000	-0.000000	-1.169174

H_2O

O	0.000000	0.000000	0.119188
H	0.000000	0.759286	-0.476754
H	-0.000000	-0.759286	-0.476754

$HCOO^-$

O	1.141497	-0.208173	0.000003
C	0.000028	0.311159	-0.000011
O	-1.141507	-0.208179	0.000003
H	-0.000082	1.463860	0.000021

HCOOH

O	-1.058869	-0.281399	-0.000173
H	-1.777777	0.367680	0.001111
C	0.130784	0.361883	0.000081
O	1.178535	-0.219172	0.000049
H	0.035742	1.465589	-0.000606
