# Supplementary material for: Probe metal binding mode of imine covalent organic frameworks: cycloiridation for (photo)catalytic hydrogen evolution from formate
Source: Chem Sci. 2021 May 11;12(22):7930–6. doi: 10.1039/d1sc01692j (PMC8188469; doi:10.1039/d1sc01692j)
Supplement: SC-012-D1SC01692J-s002 [file SC-012-D1SC01692J-s002.pdf]

Electronic Supplementary Material (ESI) for Chemical Science.
This journal is © The Royal Society of Chemistry 2021

Probe Metal Binding Mode of Imine Covalent Organic Frameworks: Cycloiridation for (Photo)catalytic Hydrogen Evolution from Formate

Jiyun Hu,[a] Hamed Mehrabi,[b] Yin-Shan Meng,[c] Maddison Taylor,[a] Jin-Hui Zhan,*[d] Qigeng Yan,[e] Mourad Benamara,[e] Robert H. Coridan,[a] Hudson Beyzavi*[a]

[a] *Department of Chemistry and Biochemistry, University of Arkansas, Fayetteville, Arkansas, 72701, United States. E-mail: beyzavi@uark.edu*

[b] *Material Science and Engineering program, University of Arkansas, Fayetteville, Arkansas, 72701, United States.*

[c] *State Key Laboratory of Fine Chemicals, Dalian University of Technology, Dalian, 116024, China.*

[d] *State Key Laboratory of Multiphase Complex System, Institute of Process Engineering, Chinese Academy of Sciences, Beijing, 100190, China. E-mail: jhzhan@ipe.ac.cn (corresponding author of the computational section)*

[e] *Institute for Nanoscience & Engineering, University of Arkansas, Fayetteville, Arkansas, 72701, United States*

Contents

1. General 3
2. Synthetic procedures 4
   2.1 Synthesis of 1,3,6,8-tetrakis(4-aminophenyl)pyrene (TAPPy) 4
   2.2 Synthesis of 1,3,5-tris(*p*-formylphenyl)benzene (TFPB) 4
   2.3 Synthesis of model Ir imine complex 5
   2.4 Synthesis of model Ir azine complex 5
   2.5 Synthesis of Py-1P 6
   2.6 Synthesis of TFPB-Hz COF 7
   2.7 General procedure for metalation with iridium 7
3. Catalytic hydrogen evolution from aqueous formate solution 18
4. Theoretical calculations 28
5. NMR spectra 29
6. References 34

1. General

Solution NMR were measured on a Bruker Ultrashield™ 400 MHz NMR spectrometer. Solid-state NMR experiments were performed on an Agilent DD2 500 MHz spectrometer (B_0 = 11.7 T) equipped with a 4.0 mm probe. ^1H-^{13}C CP/MAS NMR spectra were obtained at a spinning speed of 10 kHz, a recycle delay of 4 s, and a pulse length of 2.5 µs (π/4). FT-IR spectra were recorded on a Shimadzu IRAffinity-1S spectrophotometer. Powder X-ray diffraction (PXRD) spectra were collected on a Rigaku MiniFlex Ⅱ desktop X-Ray diffractometer. N_2 sorption isotherm carried out on a Quantachrome Autosorb iQ-MP/XR gas sorption analyzer. The morphologies of samples were inspected using a FEI Nova 200 NanoLab scanning electron microscope (SEM) and a FEI Titan 80-300 transmission electron microscope. X-ray photoelectron spectroscopy (XPS) spectra were obtained on a PHI VersaProbe XPS instrument. Inductively coupled plasma mass spectrometry (ICP-MS) was performed on a Thermo Icap Q ICP-MS instrument. Thermogravimetric analysis (TGA) was carried out on a universal Q500 TA instrument under N_2 atmosphere. pH was measured using a HANNA HI 2210 pH meter. Gas analysis was performed on an SRI 8610C gas chromatograph.

1,4-dioxane, hydrazine monohydrate, o-dichlorobenze, and 1,3,5-tribromobenzene were purchased from BeanTown Chemical. Mesitylene and 1,3,6,8-tetrabromopyrene were purchased from Tokyo Chemical Industry Co., Ltd. (TCI). Koptec's Pure Ethanol 200 Proof was obtained from Decon Labs. HPLC grade water was obtained from VWR. Sodium formate and $[Cp^*IrCl_2]_2$ were obtained from Alfa Aesar. 4-aminophenylboronic acid pinacol ester, (4-formylphenyl)boronic acid, and tetrakis(triphenylphosphine)palladium(0) were obtained from Matrix Scientific. All the chemicals were used as received.

2. Synthetic procedures

2.1 Synthesis of 1,3,6,8-tetrakis(4-aminophenyl)pyrene (TAPPy)[1]

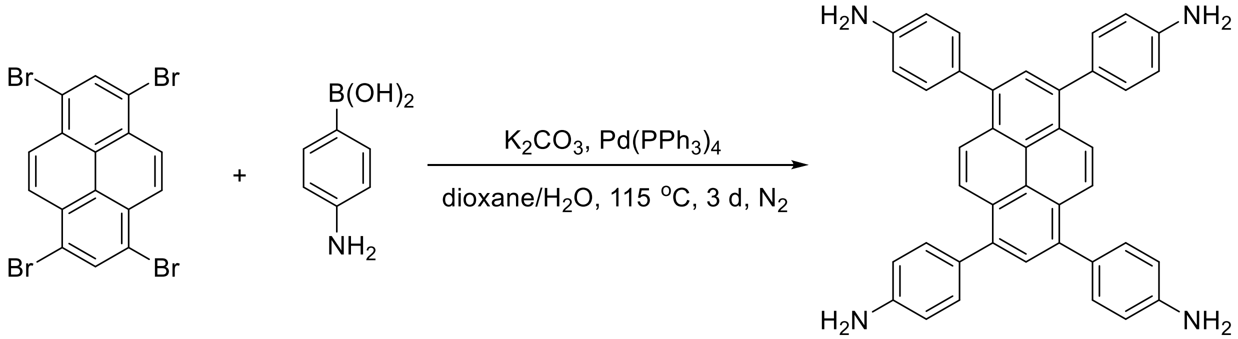

A mixture of 1,3,6,8-tetrabromopyrene (1.5 g, 2.9 mmol), 4-aminophenylboronic acid pinacol ester (3.0 g, 13.7 mmol), K_2CO_3 (2.2 g, 15.9 mmol) and $Pd(PPh_3)_4$ (330 mg, 0.29 mmol) in 32 mL 1,4-dioxane and 8 mL H_2O was refluxed for 3 d under N_2. After cooling to room temperature, H_2O was added. The resulting precipitate was collected via filtration, washed with H_2O and MeOH and then recrystallized from hot 1,4-dioxane to give the title compound as a yellow powder. Yield: 1.4 g, 82%. ^1H NMR (400 MHz, DMSO-d_6): δ 8.12 (s, 4H), 7.78 (s, 2H), 7.34 (d, J = 8.4 Hz, 8H), 6.77 (d, J = 8.4 Hz, 8H), 5.31 (s, 8H). ^{13}C NMR (101 MHz, DMSO-d_6) δ 148.7, 137.6, 131.5, 129.5, 128.1, 127.2, 126.6, 124.9, 114.4.

2.2 Synthesis of 1,3,5-tris(*p*-formylphenyl)benzene (TFPB)[2]

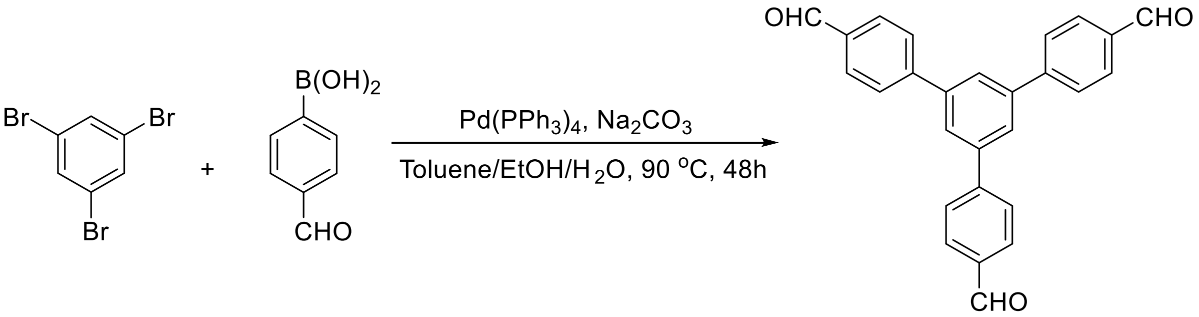

A mixture of 1,3,5-Tribromobenzene (2.5 g, 8 mmol), (4-formylphenyl)boronic acid (5.0 g, 33.3 mmol), Na_2CO_3 (8.4 g, 79.2 mmol) and $Pd(PPh_3)_4$ (1.0 g, 0.87 mmol) were dissolved in toluene (30 ml), water (5 ml) and ethanol (10 ml). The solution was degassed three times and then heated at 90 ^oC for 48 h. The organic layer was then decanted, and the aqueous layer was extracted two times using CH_2Cl_2. The combined organic layer was washed with water and evaporated. The crude product was purified by recrystallization from ethyl acetate to give the title compound TFPB as an off white solid. Yield: 2.3 g, 74%.

^{1}H NMR (400 MHz, $CDCl_3$) δ 10.12 (s, 3H), 8.08 – 8.01 (m, 6H), 7.95 – 7.87 (m, 9H). ^{13}C NMR (101 MHz, $CDCl_3$) δ 191.7, 146.3, 141.6, 135.8, 130.4, 128.0, 126.5.

2.3 Synthesis of model Ir imine complex[3]

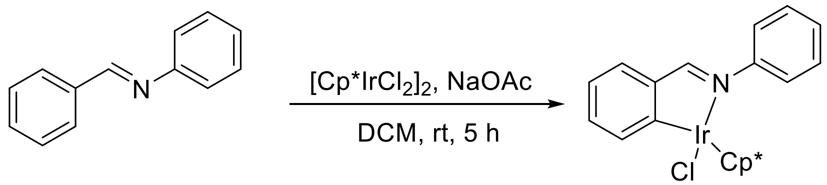

L1 **L1-Ir**

A mixture of NaOAc (13 mg, 0.16 mmol), $[Cp^*IrCl_2]_2$ (50 mg, 0.06 mmol), dibenzylideneaniline (23 mg, 0.13 mmol) and benzaldehyde (7 mg, 0.07 mmol) in 5 mL dichloromethane was stirred at room temperature for 5 h. The orange solution was passed through a syringe filter and concentrated to ca. 1 mL. Then hexane was added, and the resulting red precipitate was collected via filtration and washed with hexane. 5a was isolated as a red precipitate. Yield: 58 mg, 85%. ^{1}H NMR (400 MHz, $CDCl_3$) δ 8.34 (s, 1H), 7.87 (d, J = 7.7 Hz, 1H), 7.65 (dd, J = 7.7, 1.5 Hz, 1H), 7.62 – 7.55 (m, 2H), 7.42 (t, J = 7.7 Hz, 2H), 7.35 – 7.29 (m, 1H), 7.23 (ddd, J = 9.0, 6.6, 2.8 Hz, 2H), 7.05 (td, J = 7.4, 1.1 Hz, 1H), 1.49 (s, 15H). ^{13}C NMR (101 MHz, $CDCl_3$) δ 175.4, 170.5, 151.8, 147.0, 135.1, 132.4, 129.6, 129.0, 127.3, 122.5, 122.0, 89.2, 8.8.

2.4 Synthesis of model Ir azine complex[4]

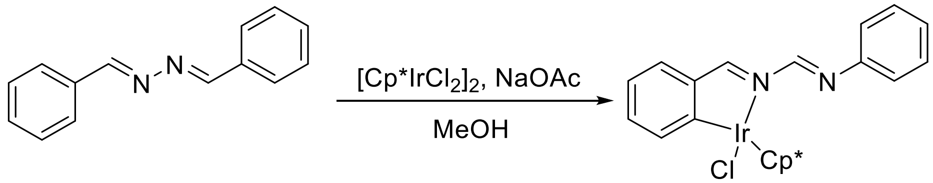

L2 **L2-Ir**

A mixture of NaOAc (150 mg, 1.8 mmol), $[Cp^*IrCl_2]_2$ (120 mg, 0.15 mmol), and 1,2-dibenzylidenehydrazine (63 mg, 0.3 mmol) was stirred at 50 °C in 15 mL of methanol for 8 h. The mixture was filtered and evaporated to give the crude product. The crude product was recrystallized from dichloromethane/hexane to give a red powder. Yield: 145 mg, 85%. ^{1}H NMR (400 MHz, $CDCl_3$) δ 9.21 (s, 1H), 8.61 (s, 1H), 7.91 (dd, J = 7.8, 1.9 Hz, 2H), 7.86 (d, J = 7.7 Hz, 1H), 7.60 (dd, J = 7.6, 1.5 Hz, 1H), 7.50 (tdd, J = 9.0, 5.8, 2.0 Hz, 3H), 7.20 (td, J = 7.5, 1.5 Hz, 1H), 7.07 (td, J = 7.4, 1.1 Hz, 1H), 1.71 (s, 15H). ^{13}C NMR (101 MHz, $CDCl_3$) δ 172.0, 160.8, 143.7, 135.1, 132.2, 131.6, 129.4, 129.0, 122.3, 89.6, 9.4.

2.5 Synthesis of Py-1P[5]

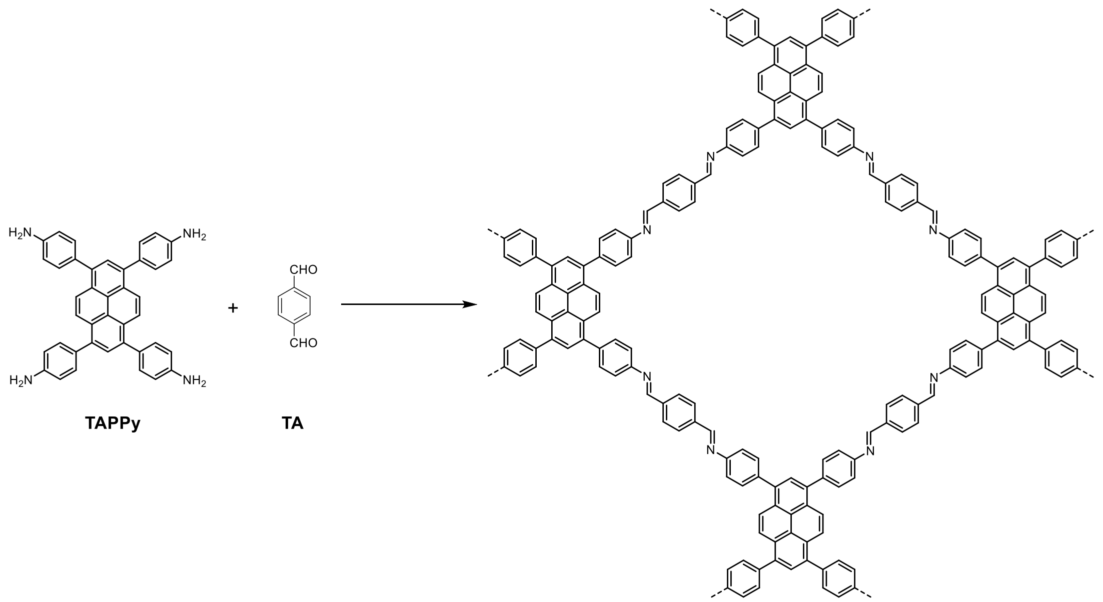

TAPPy (40.5 mg, 0.0715 mmol), TA (19.2 mg, 0.143 mmol) and 1,4-dioxane/mesitylene (1 mL, 4/1) were added into a 4 mL vial. The mixture was sonicated thoroughly (1-2 minutes). The vial was preheated to 70 °C using an aluminum heating block. To the preheated solution, 0.5 mL of 10.5 M acetic acid was added. The vial was capped securely, then gently swirled to mix the acid into the reaction mixture, then the reaction mixture was kept at 70 °C for 4 hours. After the specified reaction time, the COF solid was filtered and rinsed thoroughly with methanol and dried under vacuum at 80 °C.

2.6 Synthesis of TFPB-Hz COF[6]

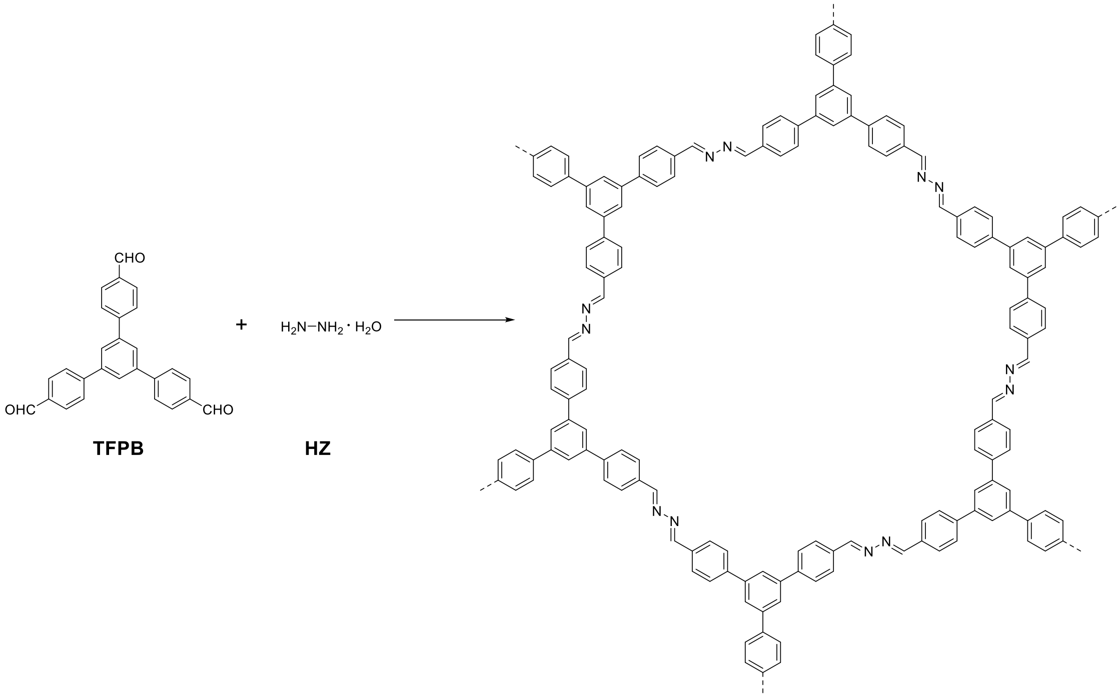

In a 10 ml Schlenk tube, TFPB (50 mg, 0.13 mmol) was suspended in a mixture *o*-dichlorobenze/EtOH/6 M acetic acid (1.2/1.8/0.3 mL). To the suspension, hydrazine hydrate (9.5 µL) was then added. The tube was degassed by three freeze-pump-thaw cycles, sealed, and then heated in an aluminum heating block at 120 °C for 3 days. Thereafter the yellow powder was filtered and washed with chloroform (2 x 5 ml), acetone (2 x 5 ml) and tetrahydrofuran (2 x 5 ml). The solid was dried in an oven at 60 °C to afford TFPB-HZ COF as a light-yellow powder.

2.7 General procedure for metalation with iridium

A mixture of COF sample (100 mg), $[Cp^*IrCl_2]_2$ (0.50 equiv. for Py-1P COF and 0.23 equiv. for TFPB-Hz COF with respect to imine), and NaOAc (10 equiv. to imine) was suspended in 10 mL dry methanol. Then the mixture was heated at 80 °C under N_2. After 24 h, the reaction mixture was cooled to room temperature. The COF powder was collected via filtration and washed with CH_2Cl_2 and MeOH, then dried under vacuum at 60 ^{o}C. The Ir loading was determined to be 11.8 wt% for Py-1P-Ir and 12.5 wt% for TFPB-Hz-Ir respectively by ICP-MS.

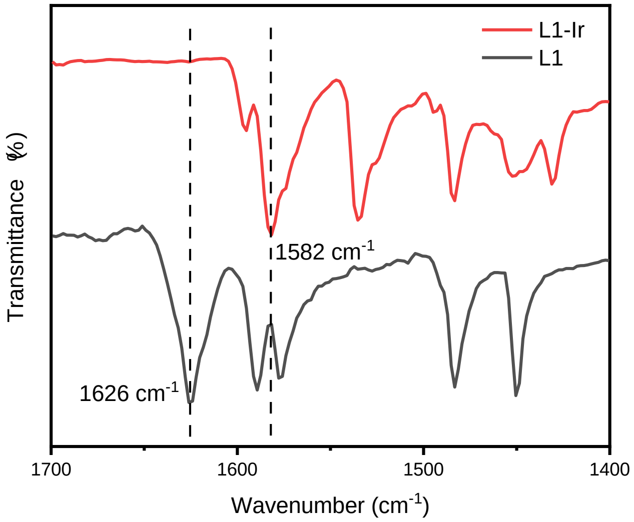

Figure S1. FT-IR spectra of L1 and L1-Ir.

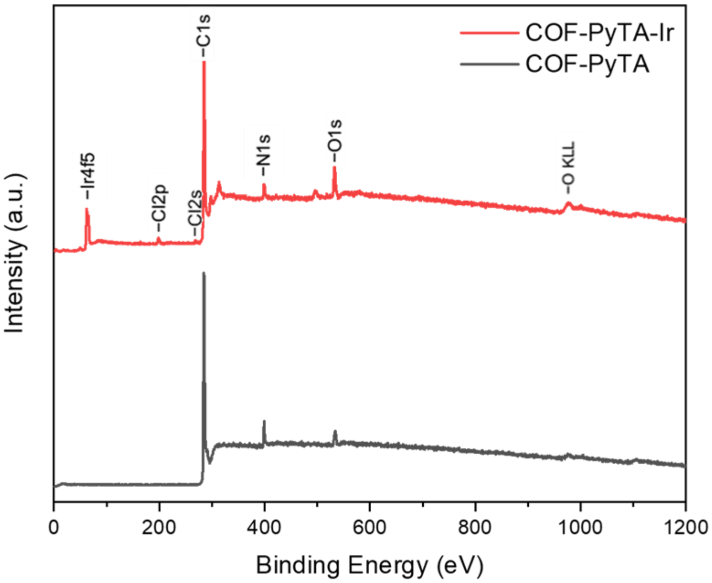

Figure S2. XPS survey spectra of Py-1P COF and Py-1P-Ir COF.

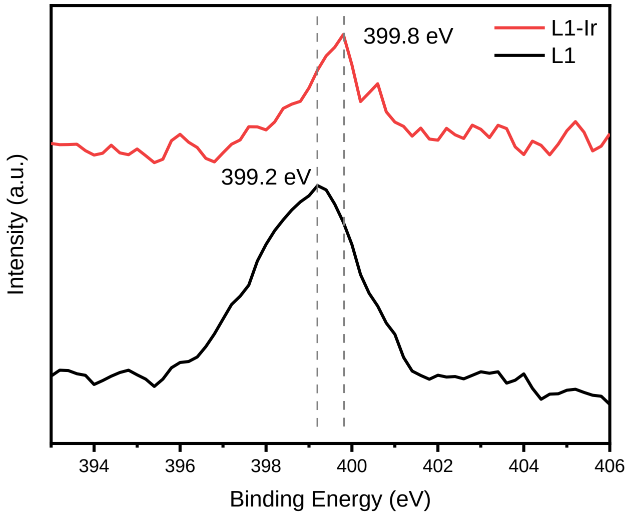

Figure S3. XPS N 1s spectra of L1 and L1-Ir.

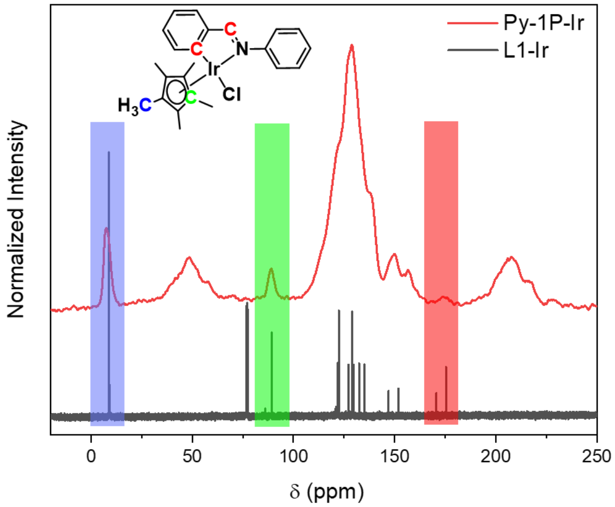

Figure S4. Comparison of ^{13}C NMR spectra of L1-Ir (in $CDCl_3$) and Py-1P-Ir COF (solid state). Color code: blue, methyl carbons; green, aromatic carbons of Cp*; red, imine carbon and Ir bonded carbon in the iridacycle.

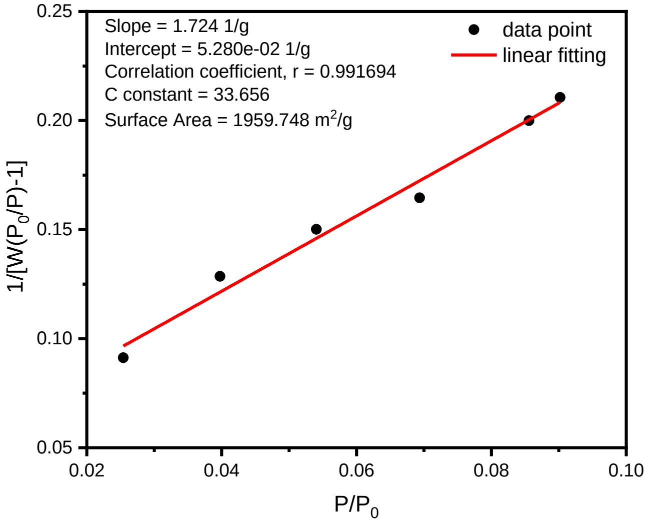

Figure S5. BET plot of Py-1P COF.

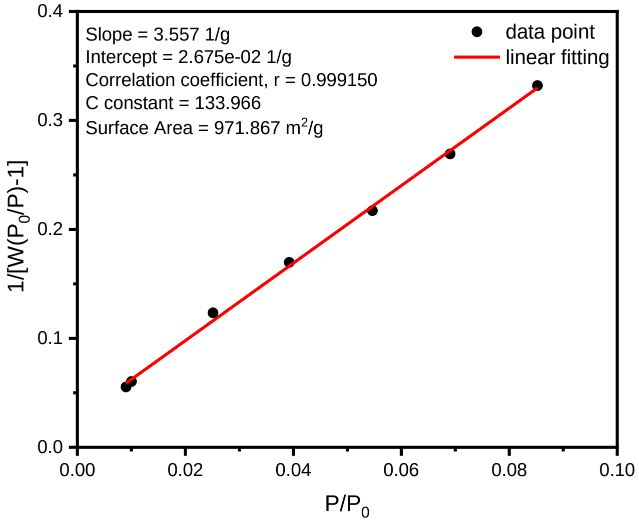

Figure S6. BET plot of Py-1P-Ir COF.

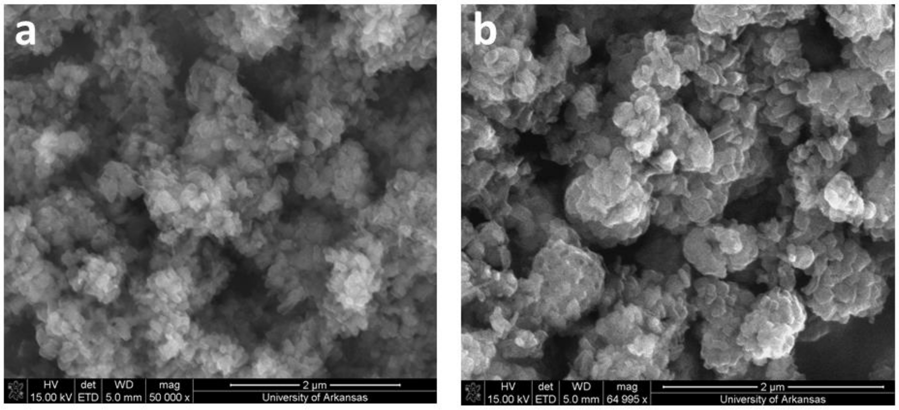

Figure S7. SEM images of (a) Py-1P COF and (b) Py-1P-Ir COF.

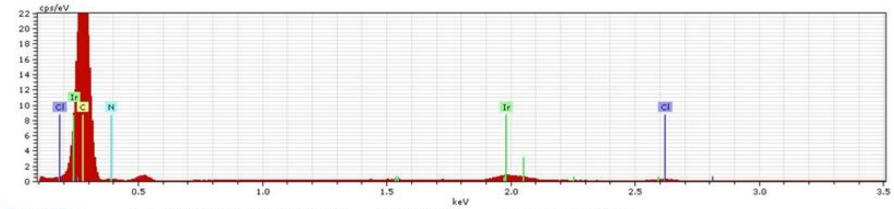

Acquisition Date:6/16/2020 10:31:15 PM HV:15.0kV Puls th.:3.00kcps

El	AN	Series	unn. C [wt.%]	norm. C [wt.%]	Atom. C [at.%]	Error (1 Sigma) [wt.%]
C	6	K-series	89.27	89.27	92.15	9.71
N	7	K-series	8.64	8.64	7.65	1.55
Cl	17	K-series	0.23	0.23	0.08	0.03
Ir	77	M-series	1.85	1.85	0.12	0.10
		Total:	100.00	100.00	100.00	

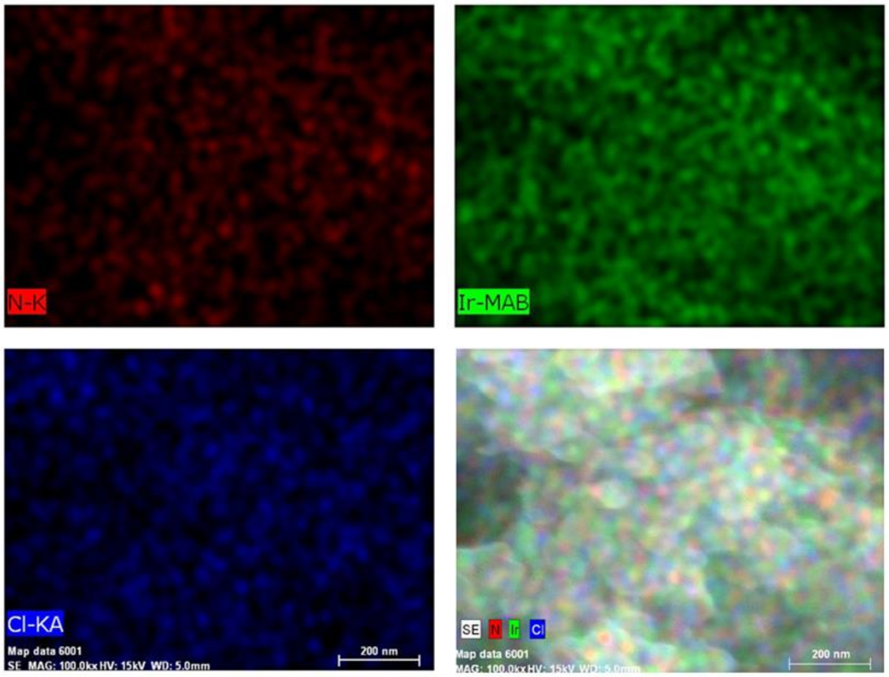

Figure S8. EDX spectrum of Py-1P-Ir COF.

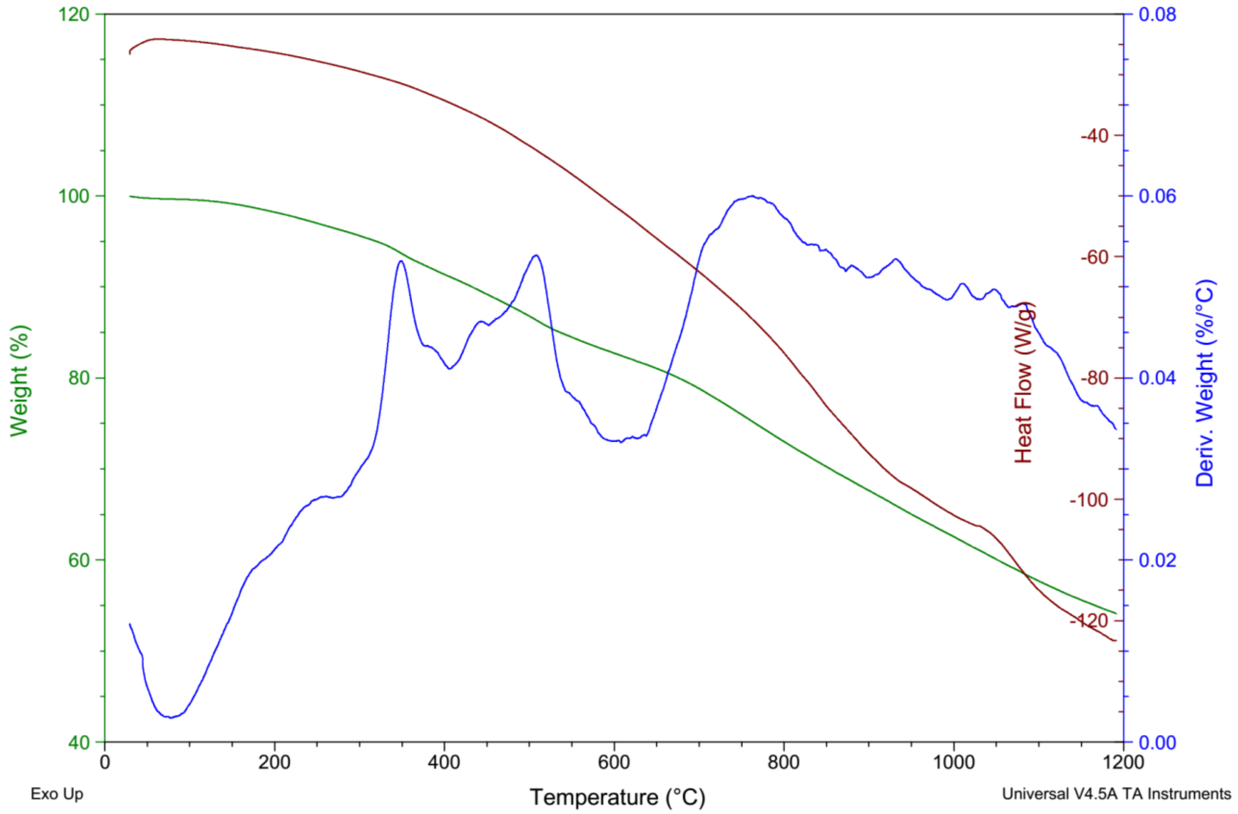

Figure S9. TGA trace of Py-1P-Ir COF.

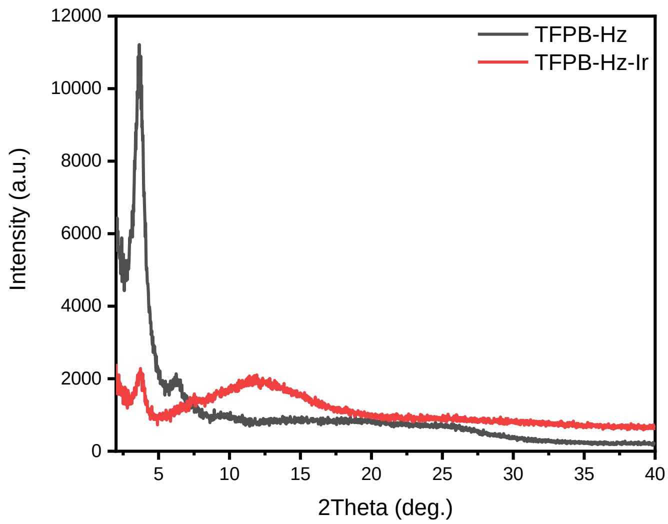

Figure S10. PXRD patterns of TFPB-Hz COF and TFPB-Hz-Ir COF.

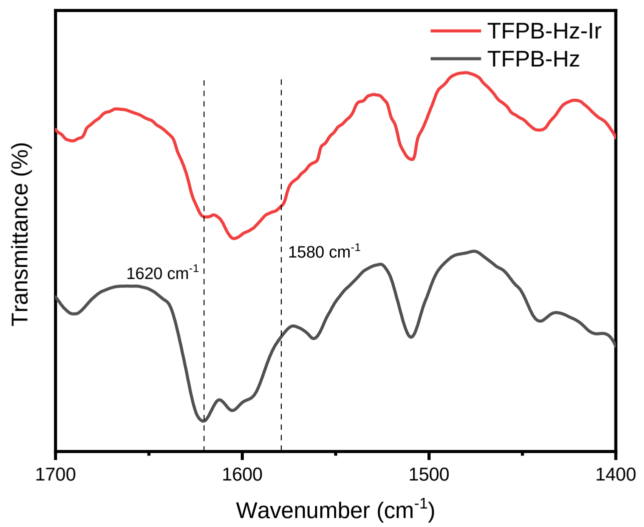

Figure S11. FT-IR spectra of TFPB-Hz COF and TFPB-Hz-Ir COF.

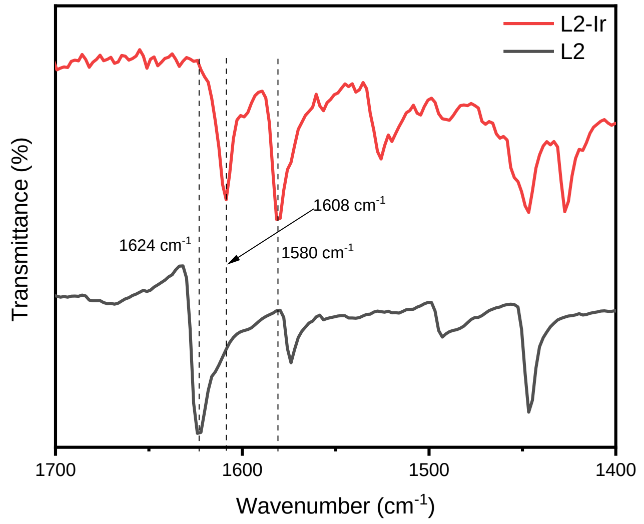

Figure S12. FT-IR spectra of L2 and L2-Ir.

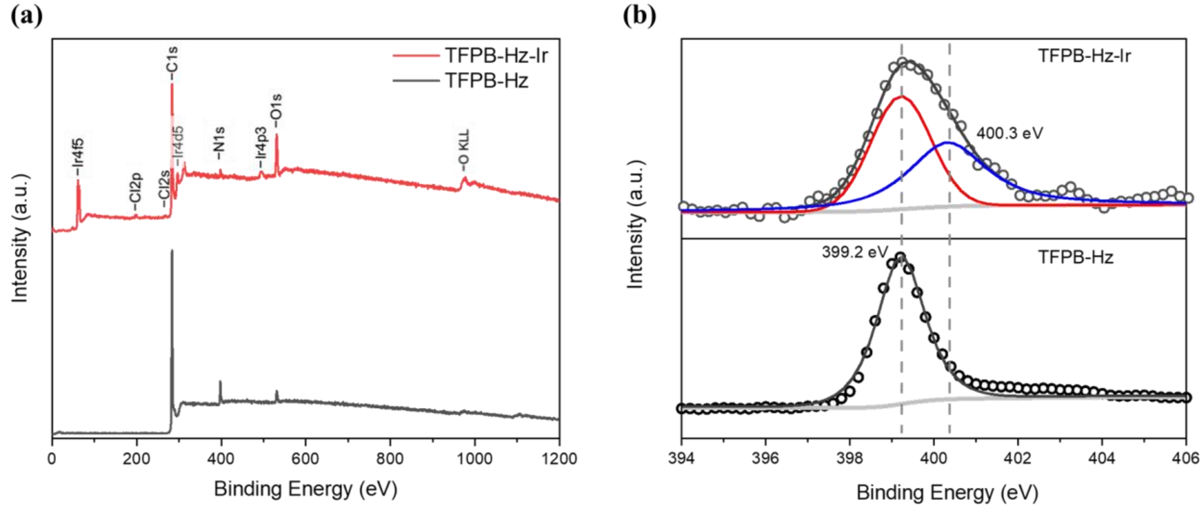

Figure S13. XPS survey (a) and N 1s (b) spectra of TFPB-Hz COF and TFPB-Hz-Ir COF.

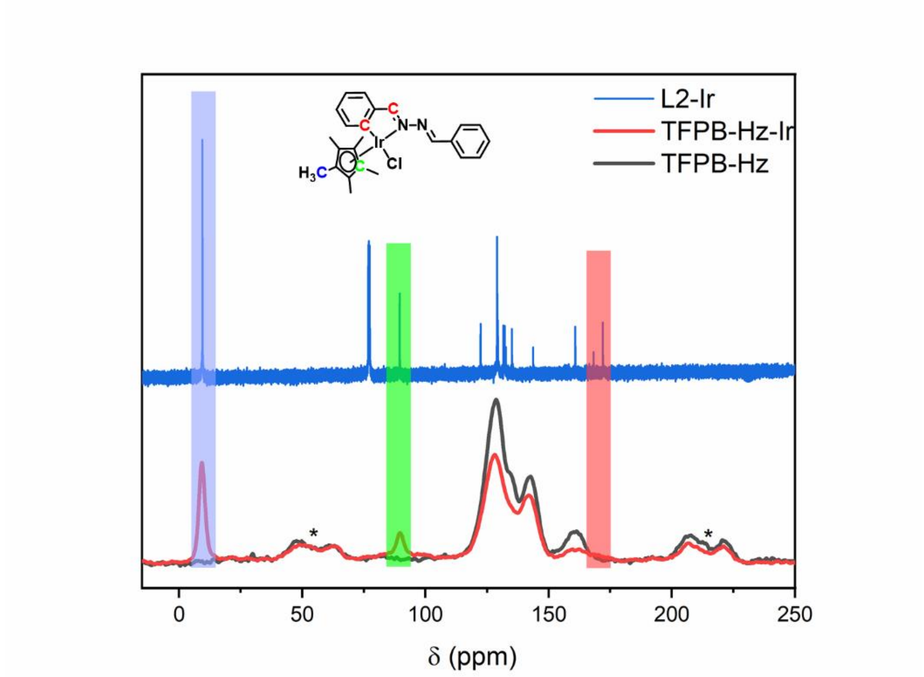

Figure S14. Comparison of ^{13}C NMR spectra of L2-Ir (in $CDCl_3$), TFPB-Hz COF and TFPB-Hz-Ir COF (solid state). Color code: blue, methyl carbons; green, aromatic carbons of Cp*; red, imine carbon and Ir bonded carbon in the iridacycle.

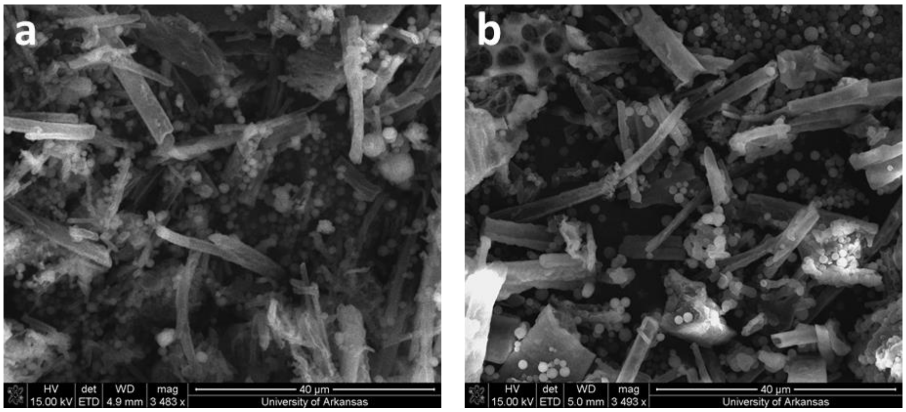

Figure S15. SEM images of (a) TFPB-Hz COF and (b) TFPB-Hz-Ir COF.

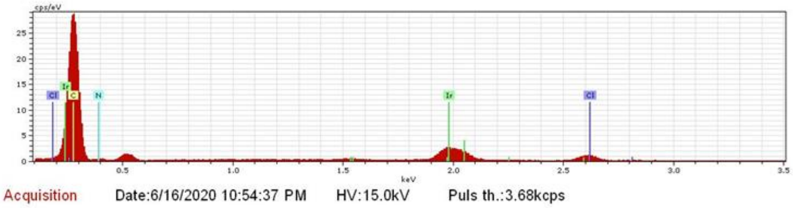

El	AN	Series	unn. C [wt.%]	norm. C [wt.%]	Atom. C [at.%]	Error (1 Sigma) [wt.%]
C	6	K-series	82.87	82.87	89.50	9.61
N	7	K-series	10.48	10.48	9.71	2.18
Cl	17	K-series	1.16	1.16	0.42	0.07
Ir	77	M-series	5.49	5.49	0.37	0.25

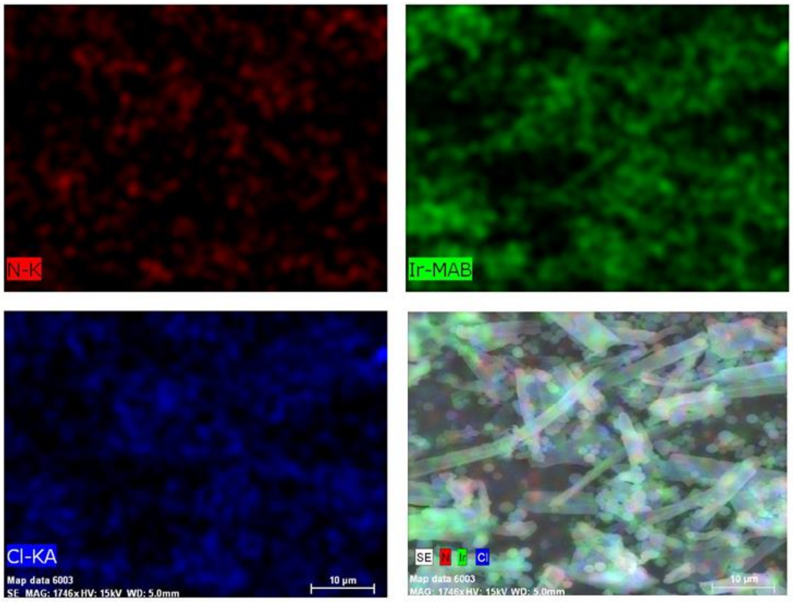

Figure S16. EDX spectrum of TFPB-Hz-Ir COF.

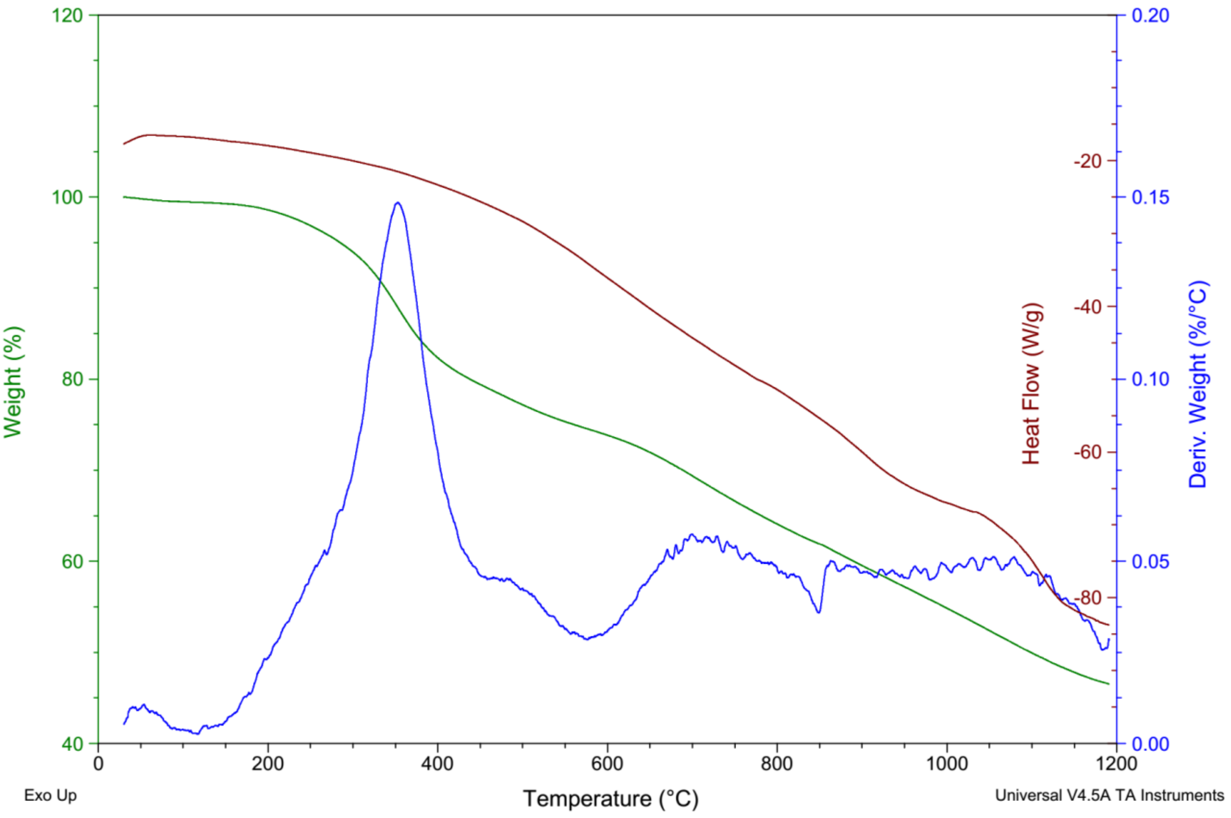

Figure S17. TGA trace of TFPB-Hz-Ir COF.

3. Catalytic hydrogen evolution from aqueous formate solution

General procedure. Catalyst (6.35 μmol based on Ir) and 1 M HCOONa aqueous solution (10 mL) were added into a 25 mL Schlenk tube. The tube was capped with a rubber septum and degassed by bubbling through N_2 for 15 min. Then the tube was heated in an oil bath (thermal reaction) or illuminated with a LED photoreactor (photoreaction, Figure S19) for 6 h. The gas in the head space was analyzed by GC.

Temperature effect. Py-1P-Ir COF (10 mg, 6.35 μmol based on Ir) and 1 M HCOONa aqueous solution (10 mL) were added into a 25 mL Schlenk tube. The tube was capped with a rubber septum and degassed by bubbling through N_2 for 15 min. Then the tube was heated in an oil bath for 6 h.

pH effect. Py-1P-Ir COF (10 mg, 6.35 μmol based on Ir) and 1 M pH adjusted formate aqueous solution (10 mL) were added into a 25 mL Schlenk tube. The tube was capped with a rubber septum and degassed by bubbling through N_2 for 15 min. Then the tube was heated in an oil bath at 25 ^oC for 6 h. **pH adjustment:** Acidic 1.0 M formate solutions were prepared by mixing approiate HCOOH and HCOONa in H_2O, keeping the concentration of formate at 1 M accordding to Table S1.

Recycle test. The reaction was carried out with 10 mg catalyst in 1 M HCOONa at 85 ^oC for 6 h. The COF catalyst was revovered by centrifugation, washed with MeOH, then dried at 65 ^oC before it was used for next run.

Concentration effect. Py-1P-Ir COF (10 mg, 6.35 μmol based on Ir) and 1 M, 2 M, 5 M, or 10 M HCOONa aqueous solution (10 mL) were added into a 25 mL Schlenk tube. The tube was capped with a rubber septum and degassed by bubbling through N_2 for 15 min. Then the tube was heated in an oil bath at 65 ^oC for 6 h. **Note: the pH was not adjusted.**

Catalytic reduction of imine substrate. Py-1P-Ir or Py-1P COF (6 mg), dibenzylideneaniline (45 mg, 0.25 mmol), and 1M HCOONa (5 mL) were added into a 25 mL Schlenk tube. The tube was capped with a rubber septum and degassed by bubbling through N_2 for 15 min. Then the tube was heated in an oil bath at 85 ^oC for 6 h. The organic compound was extracted with CH_2Cl_2 and analyzed by ^1H NMR. An NMR yield of 77% was observed for the formation of N-benzylaniline in the presence of **Py-1P-Ir**. **Py-1P** COF failed to catalyze the reaction (Figure S25).

Table S1. Recipe of 100 mL formate (1.0 M) solutions with different pHs.

Entry	HCOOH / g	HCOONa / g	pH
1	4.309	0.114	1.94
2	1.624	4.280	3.72
3	0.026	6.761	5.92
4	0	6.801	7.52

Table S2. Ir loading effect on the HER reaction.[a]

Catalyst[b]	Ir loading[c]	H_2 / µmol (purity, %)	H_2 generation rate / µmol g^{-1} h^{-1}	TON
Py-1P-Ir_{100}	11.8%	250.0 (93)	4167	40.7
Py-1P-Ir_{50}	9.9%	125.8 (96)	2097	24.4
Py-1P-Ir_{25}	8.7%	98.1 (96)	1635	21.7

[a] Reaction conditions: **A 10 mL of** 1 M HCOONa aqueous solution were heated at 85 °C for 6 h in the presence of 10 mg Py-1P-Ir COF. [b] The subscript denotes the equivalent of Ir relative to imine used for metalation. [c] Ir loading determined by ICP.

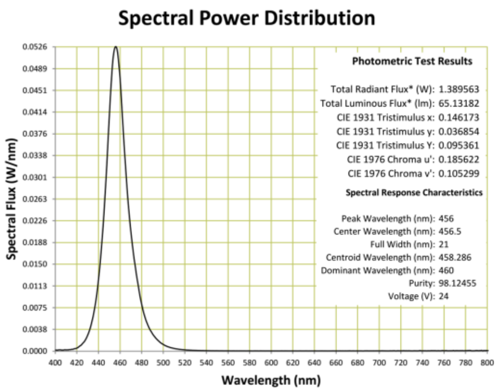

Figure S18. Test report of the blue LED light purchased from Environmental Lights[7].

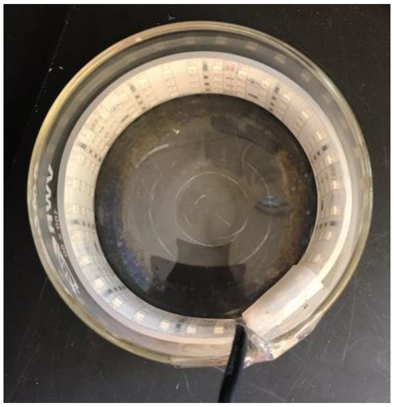

Figure S19. Photograph of the LED photoreactor. The LED light strip (320×33×7.5 mm) is glued onto the inner wall of a 100×50 mm crystallizing dish.

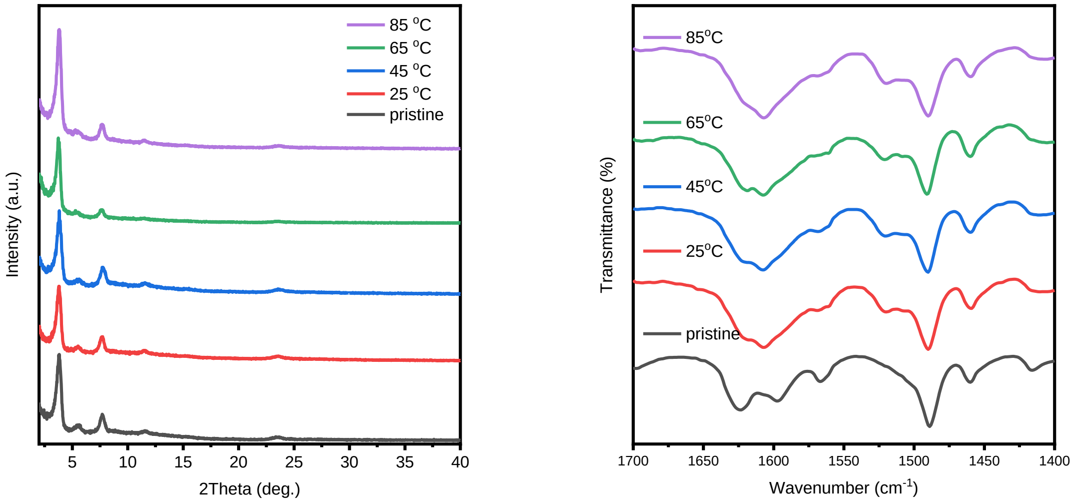

Figure S20. PXRD patterns (left) and FT-IR (right) spectra of recovered Py-1P-Ir COF from different reaction temperatures.

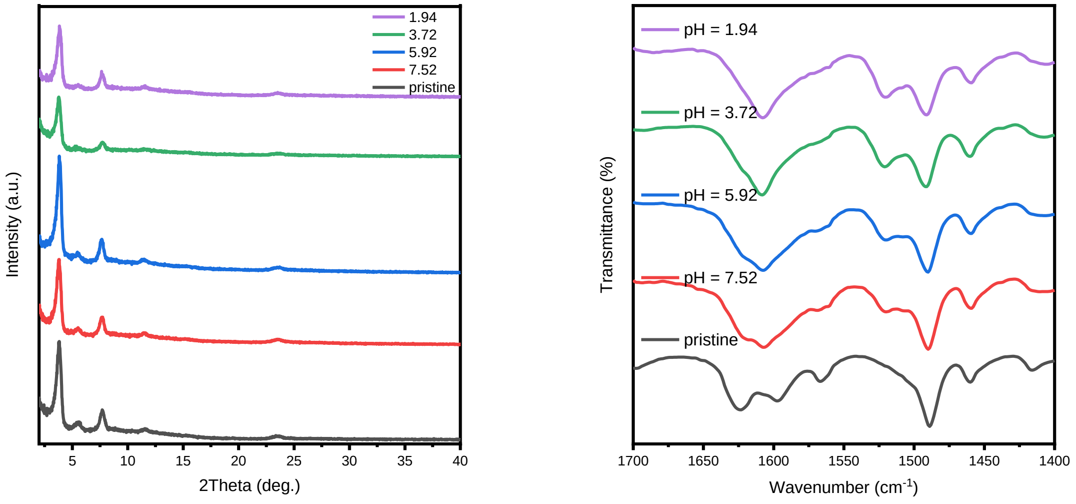

Figure S21. PXRD (left) patterns and FT-IR (right) spectra of recovered Py-1P-Ir COF from different reaction pHs.

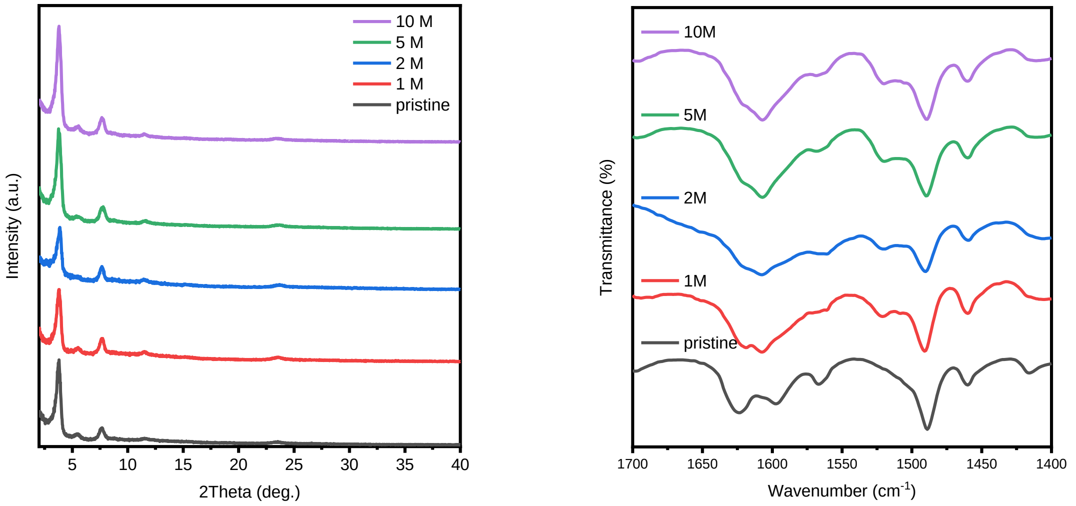

Figure S22. PXRD patterns (left) and FT-IR (right) spectra of recovered Py-1P-Ir COF from different reaction HCOONa concentrations.

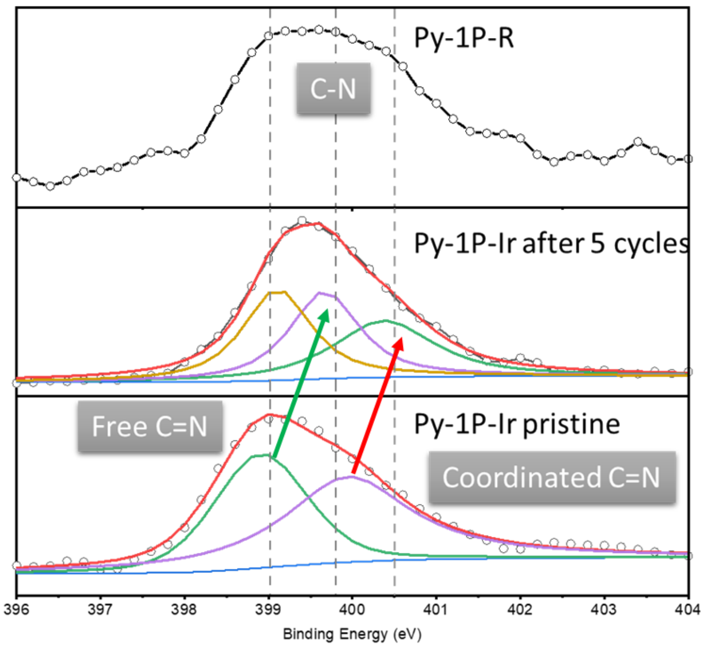

Figure S23. XPS N1S spectra of Py-1P-Ir COF after catalysis (Py-1P-R where R means imine reduced was prepared using HCOOH as a reductant according to a recent publication).[8]

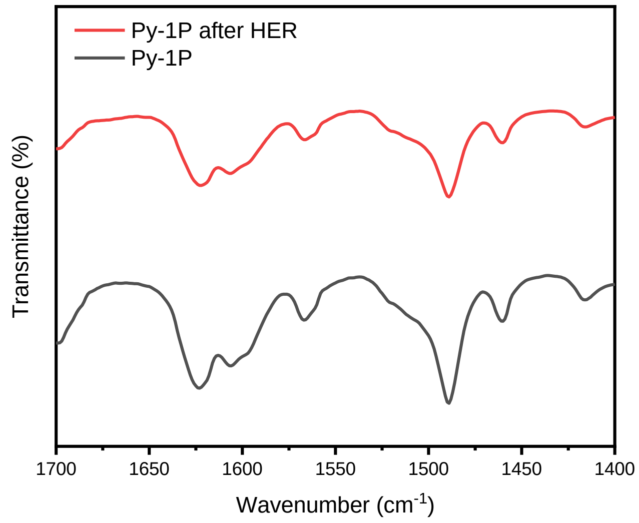

Figure S24. FT-IR spectra of Py-1P COF before and after HER at 65 °C for 6 h.

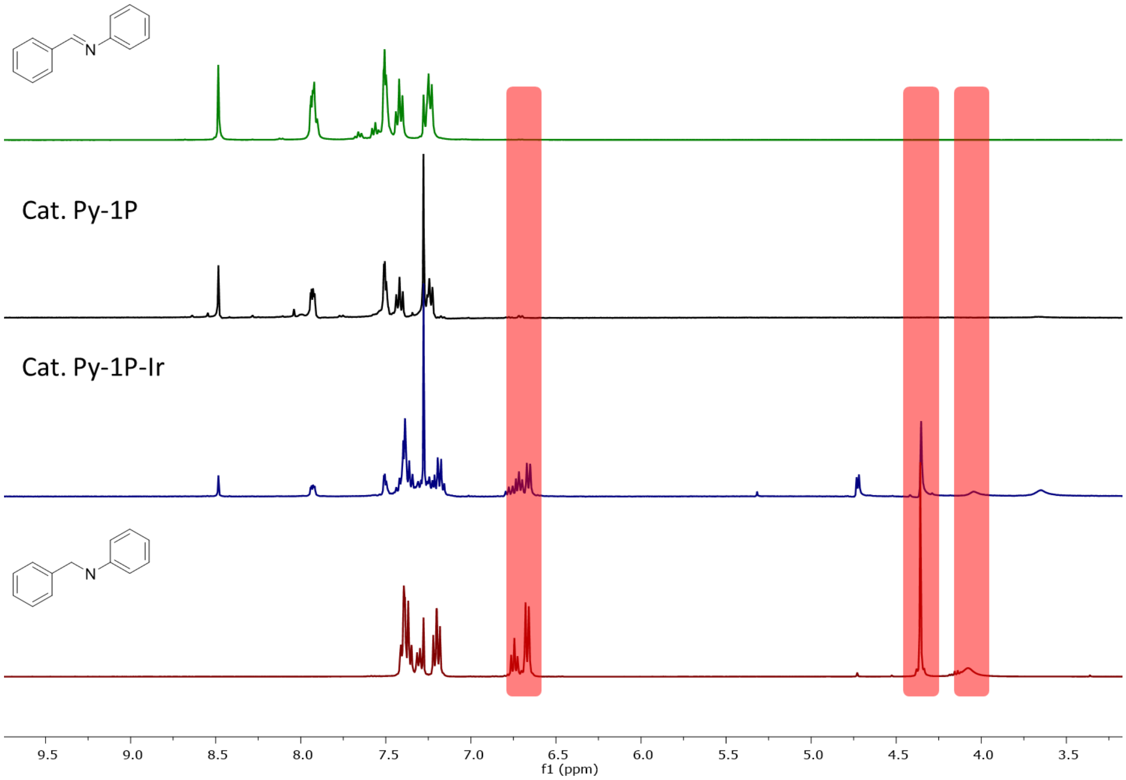

Figure S25. ^{1}H NMR spectra of dibenzylideneaniline (green line), reaction mixture of Py-1P (black line) and Py-1P-Ir COF (blue line) catalyzed transfer hydrogenation, and N-benzylaniline (maroon line) in $CDCl_3$.

Table S3. Ir concentration of the reaction filtrate determined by ICP.

Entry	pH	Temperature / °C	Formate concentration / M	Ir concentration / ppb
1	7.52	25	1	45
2	7.52	45	1	31
3	7.52	65	1	37
4	7.52	85	1	23
5	5.92	25	1	49
6	3.72	25	1	30
7	1.94	25	1	60
8	-	65	2	27
9	-	65	5	31
10	-	65	10	43

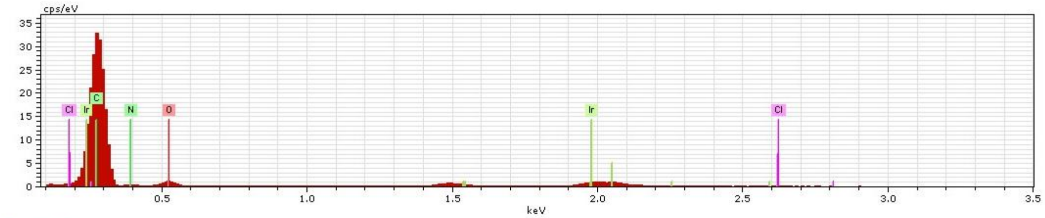

Acquisition Date:9/18/2020 9:19:43 PM HV:15.0kV Puls th.:3.23kcps

El	AN	Series	unn. C [wt.%]	norm. C [wt.%]	Atom. C [at.%]	Error (1 Sigma) [wt.%]
C	6	K-series	82.74	82.74	86.98	8.99
N	7	K-series	7.84	7.84	7.07	1.37
O	8	K-series	7.37	7.37	5.81	1.07
Cl	17	K-series	0.00	0.00	0.00	0.00
Ir	77	M-series	2.05	2.05	0.13	0.11
		Total:	100.00	100.00	100.00	

Figure S26. EDX spectrum of Py-1P-Ir COF after one HER cycle.

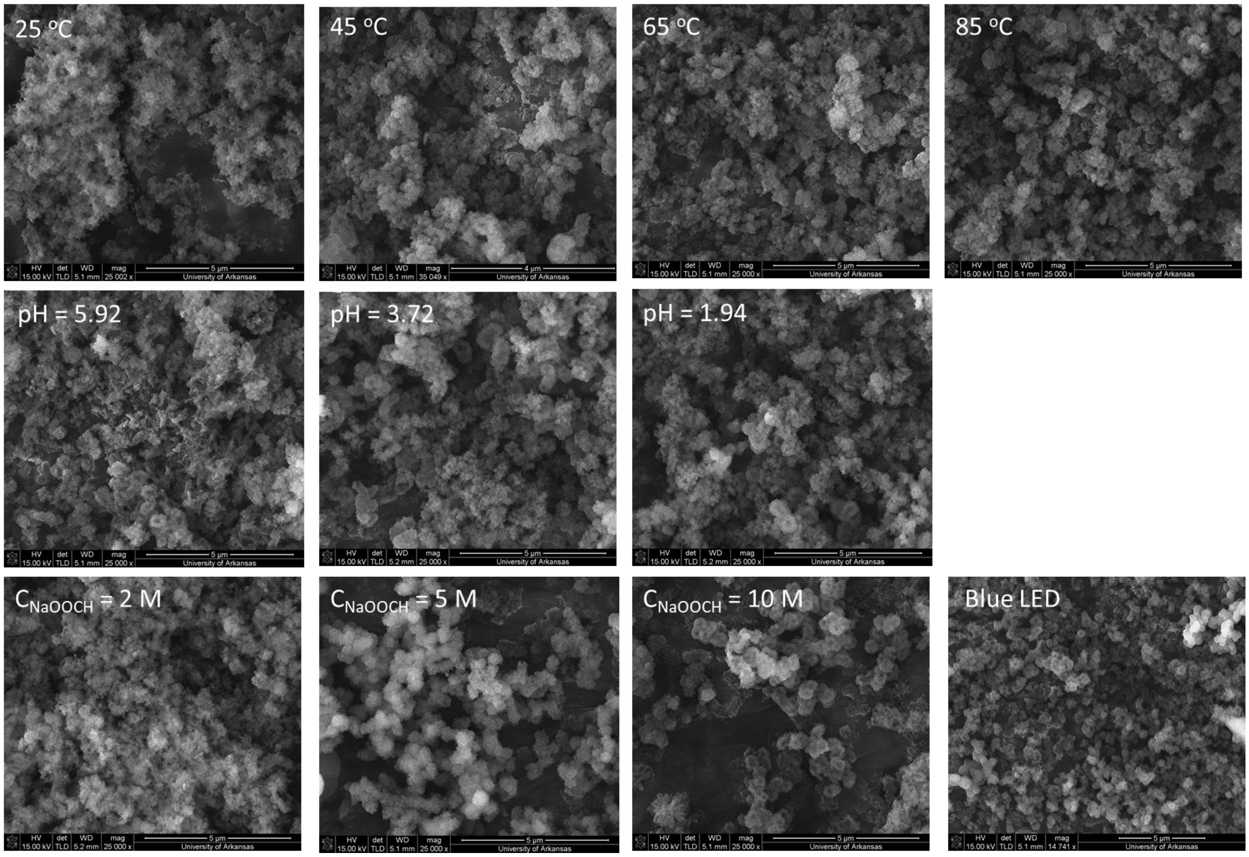

Figure S27. SEM images of recovered Py-1P-Ir COF from different conditions.

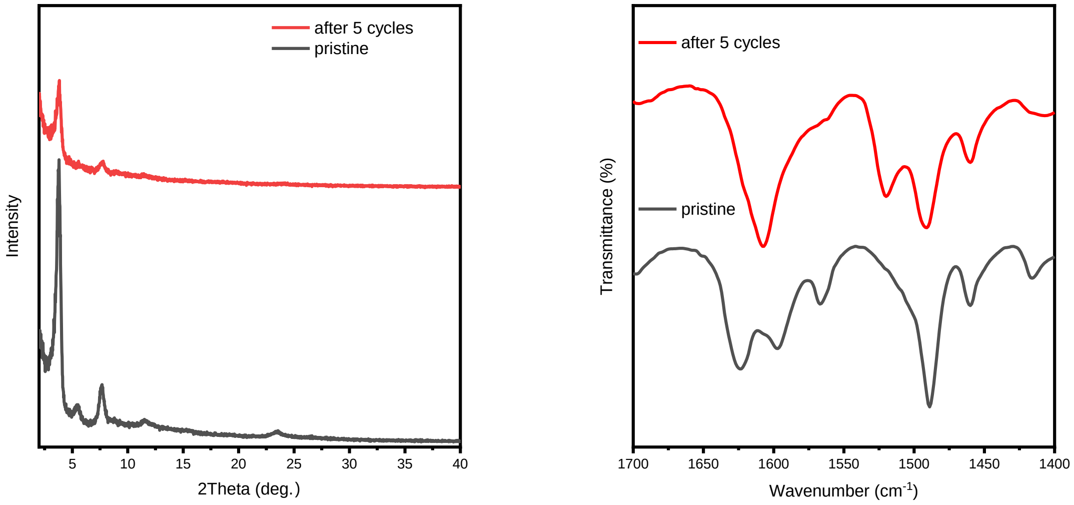

Figure S28. PXRD patterns (left) and FT-IR (right) spectra of recovered Py-1P-Ir COF after five consecutive cycles from 1 M HCOONa at 85 °C.

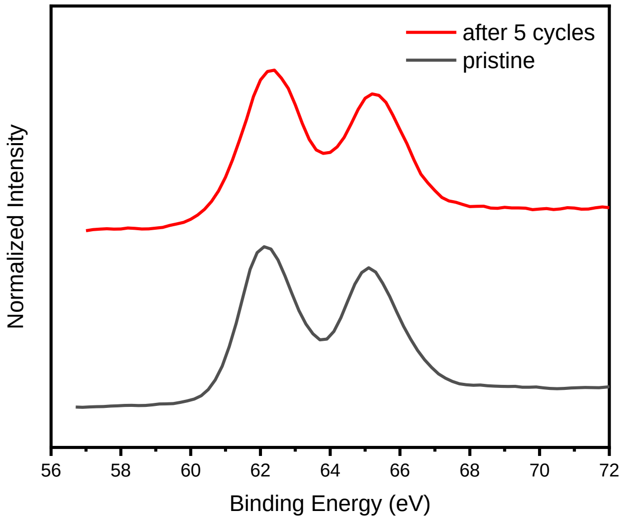

Figure S29. XPS Ir 4f spectra of Py-1P-Ir COF after catalysis.

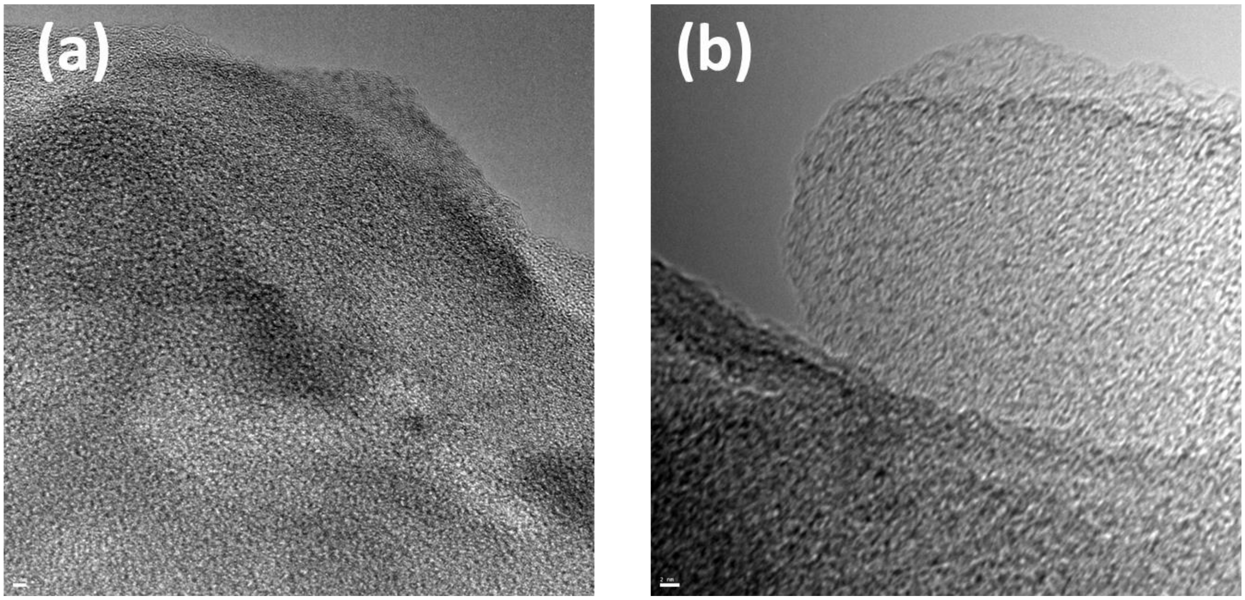

Figure S30. TEM images of Py-1P-Ir (a) before catalysis (scale bar 50 nm) and (b) after five catalytic cycles of HER reaction (scale bar 2 nm).

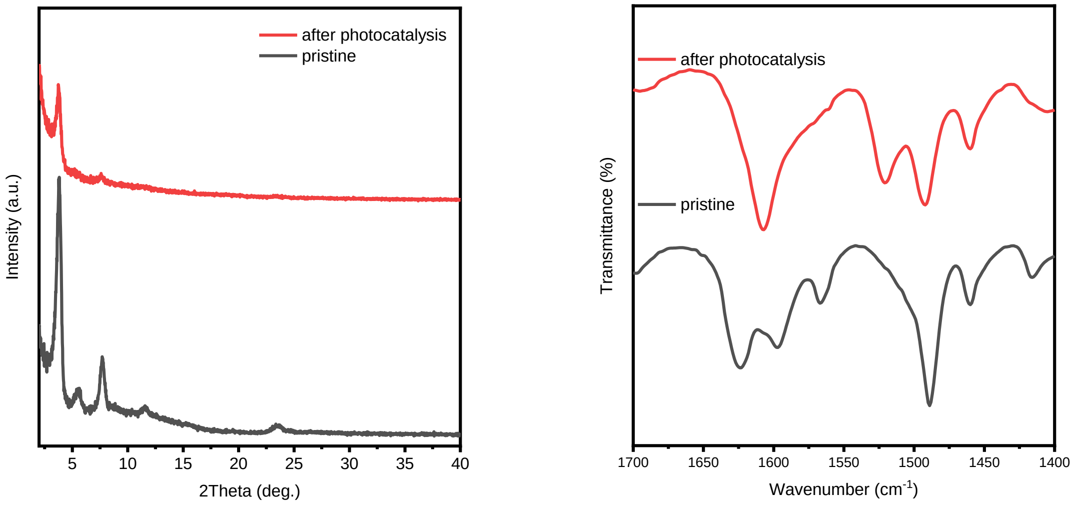

Figure S31. PXRD patterns (left) and FT-IR (right) spectra of recovered Py-1P-Ir COF from photoreactions.

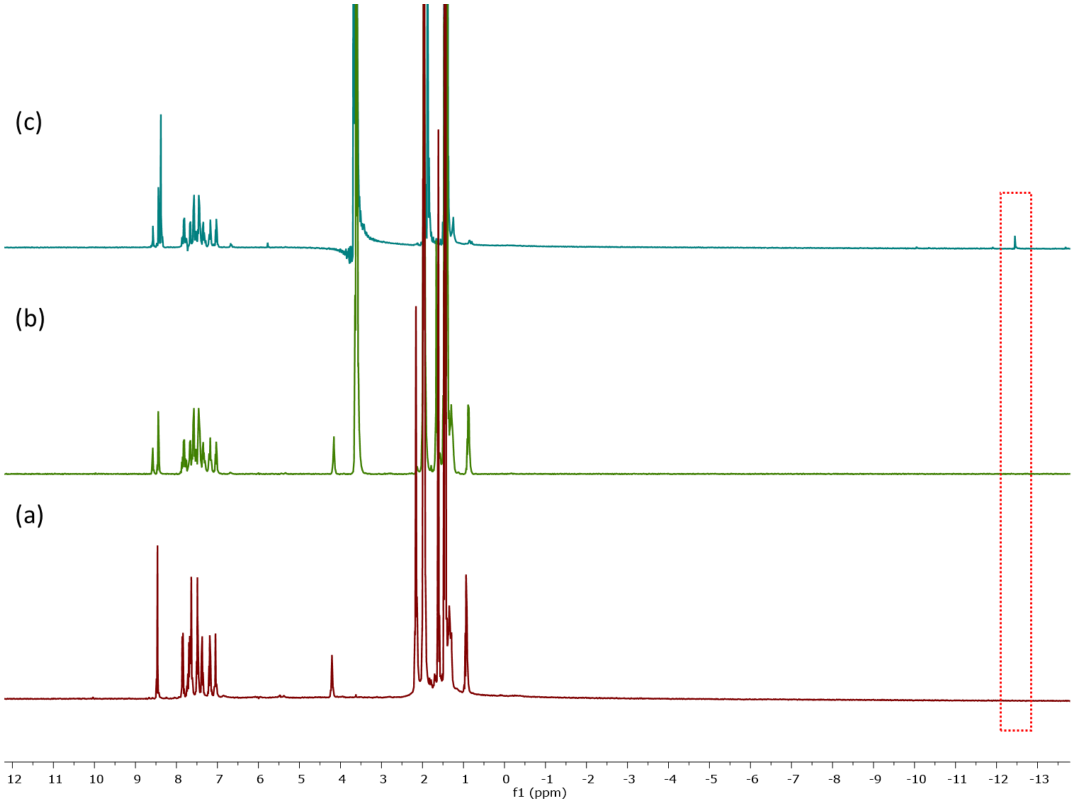

Figure S32. ^{1}H NMR spectra of (a) L1-Ir in CD_3CN, (b) L1-Ir in CD_3CN/D_2O = 4/1, (c) L1-Ir in CD_3CN/D_2O = 4/1 in the presence of excess HCOONa. Note: The low solubility of L1-Ir in water prevented the NMR studies in pure water, thus a mixture of CD_3CN/D_2O = 4/1 was used.

4. Theoretical calculations

Theoretical calculations were implemented with the Gaussian 16 software package.[9] The geometry optimization of the stationary points was carried out by using B3LYP hybrid functional[10, 11] with 6-31G(d,p) basis set for the main group atoms and the effective core potentials (ECPs) of Hay and Wadt with the LanL2DZ double-valence basis set[12-14] for Ir. D3 dispersion correction developed by Grimme[15] was included to correct the weak interactions. The vibrational frequency calculations at the same level were also carried out to verify each stationary point to be either a minimum or transition state (TS). Intrinsic reaction coordinate (IRC) calculations were used to confirm that each TS can connect to the corresponding reactant and product. Gibbs free energies were calculated with considering the solvent effect based on the solvation model density (SMD) solvent model,[16] using water as the solvent which was used in the experiment. To keep the computational study efficient, only one COF unit was used in the calculation.

5. NMR spectra

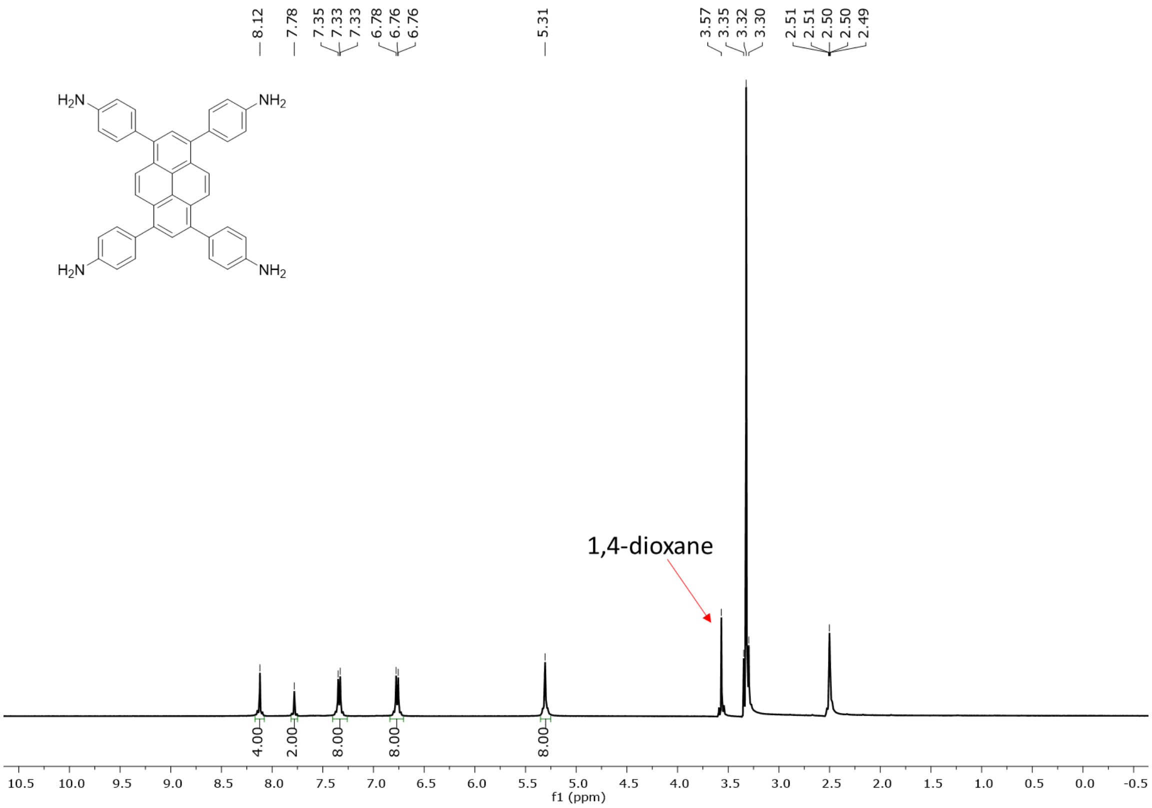

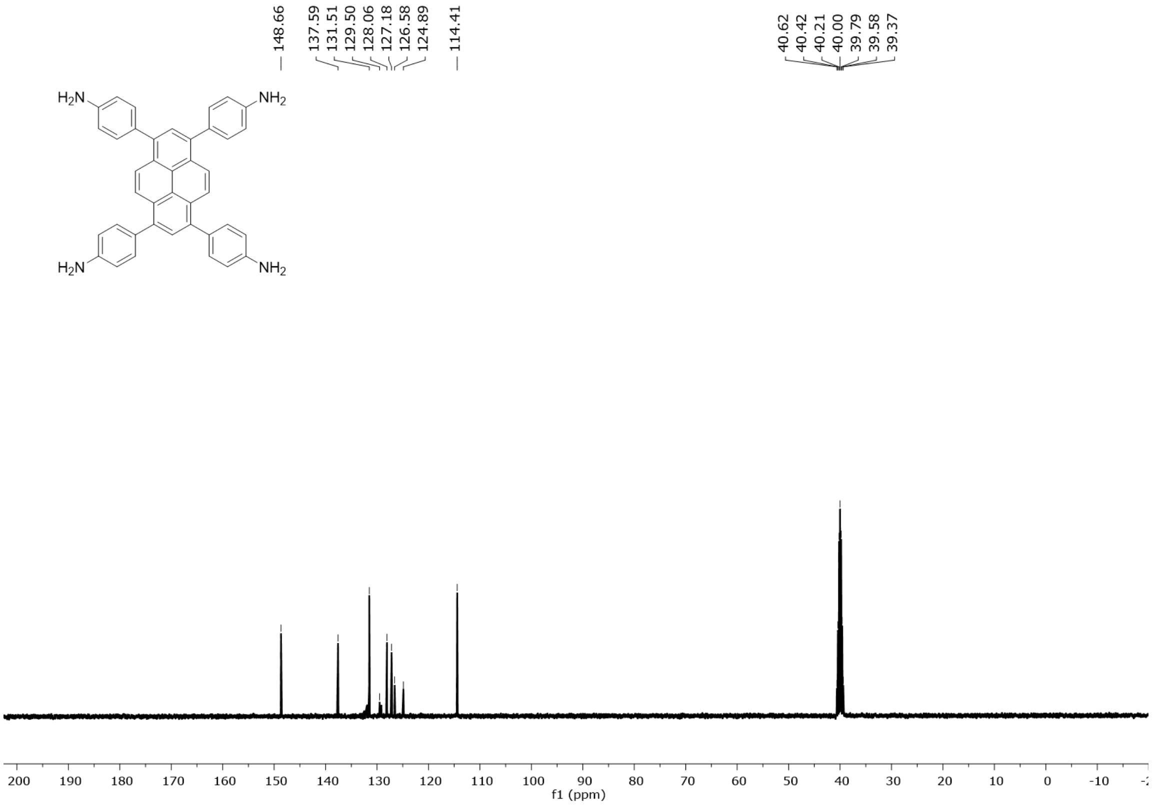
148.66
137.59
131.51
129.50
128.06
127.18
126.58
124.89
114.41
40.62
40.42
40.21
40.00
39.79
39.58
39.37
H2N
NH2
H2N
NH2
200 190 180 170 160 150 140 130 120 110 100 90 80 70 60 50 40 30 20 10 0 -10
f1 (ppm)

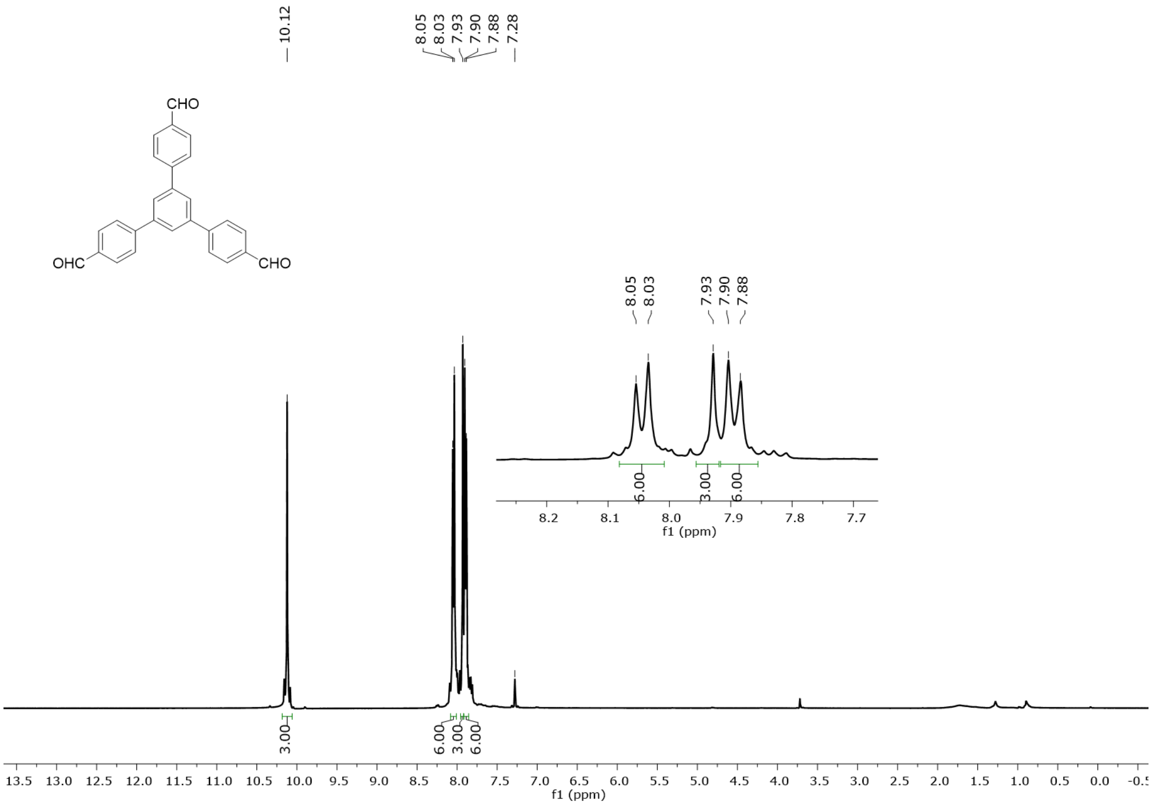
10.12
8.05
8.03
7.93
7.90
7.88
7.28
CHO
OHC
CHO
3.00
6.00
3.00
6.00
f1 (ppm)

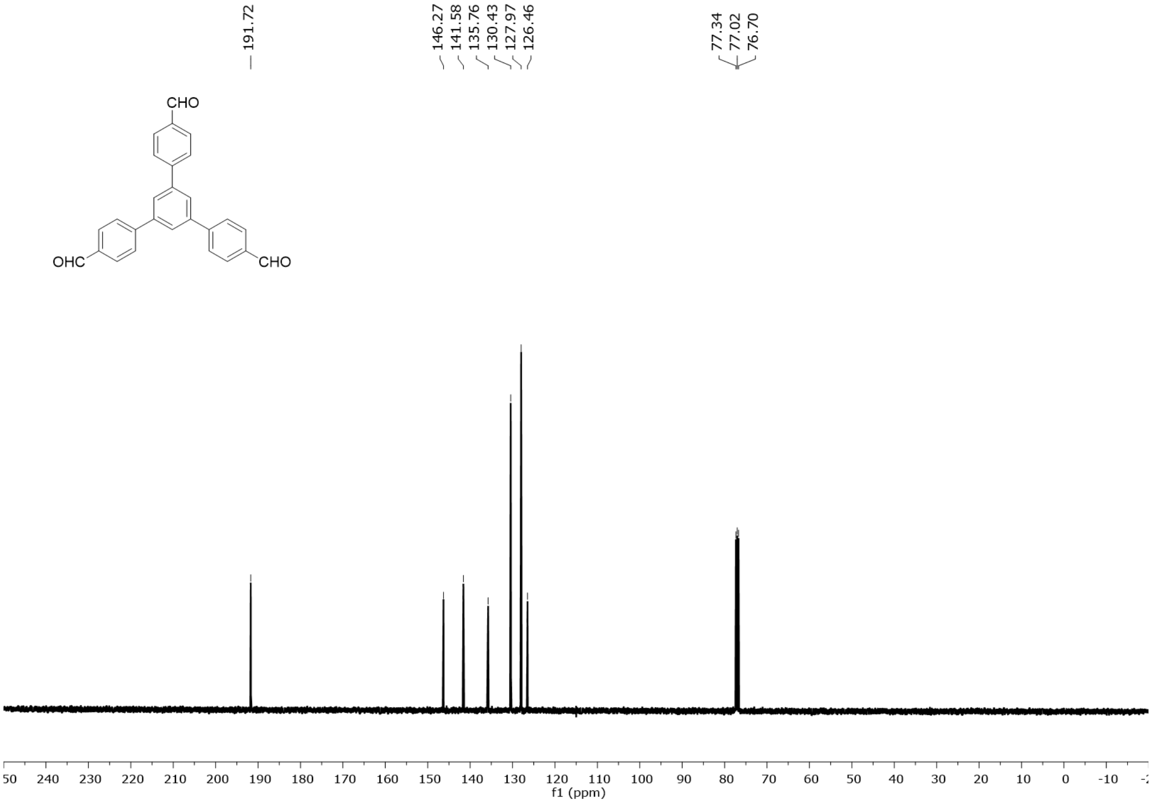
191.72
146.27
141.58
135.76
130.43
127.97
126.46
77.34
77.02
76.70
CHO
OHC
CHO
f1 (ppm)

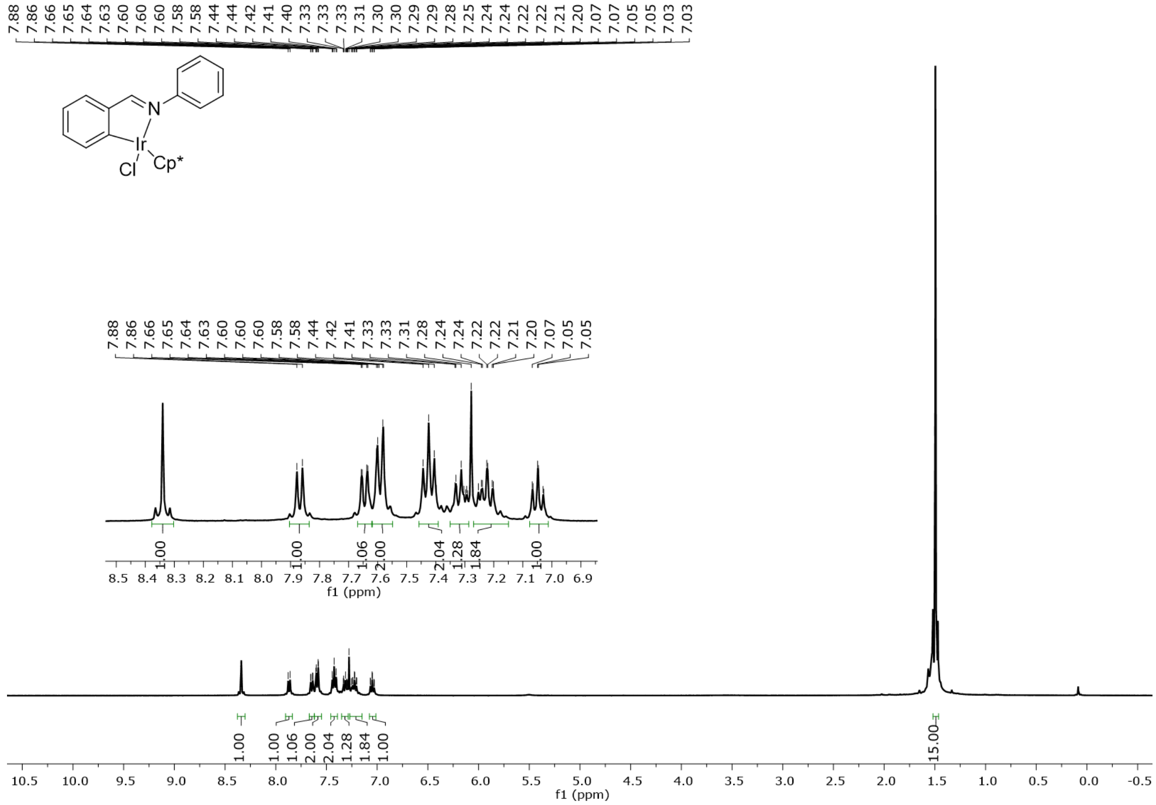
7.88 7.86 7.66 7.65 7.64 7.63 7.60 7.60 7.60 7.58 7.58 7.44 7.44 7.42 7.41 7.40 7.33 7.33 7.33 7.31 7.30 7.30 7.29 7.29 7.28 7.25 7.24 7.24 7.22 7.22 7.21 7.20 7.07 7.07 7.05 7.03 7.03
N
Ir
Cl
Cp*
7.88 7.86 7.66 7.65 7.64 7.63 7.60 7.60 7.60 7.58 7.58 7.44 7.42 7.41 7.33 7.33 7.31 7.28 7.24 7.24 7.22 7.22 7.21 7.20 7.07 7.05 7.05
1.00 1.00 1.06 2.00 2.04 1.28 1.84 1.00
8.5 8.4 8.3 8.2 8.1 8.0 7.9 7.8 7.7 7.6 7.5 7.4 7.3 7.2 7.1 7.0 6.9
f1 (ppm)
1.00 1.00 1.06 2.00 2.04 1.28 1.84 1.00 15.00
10.5 10.0 9.5 9.0 8.5 8.0 7.5 7.0 6.5 6.0 5.5 5.0 4.5 4.0 3.5 3.0 2.5 2.0 1.5 1.0 0.5 0.0 -0.5
f1 (ppm)

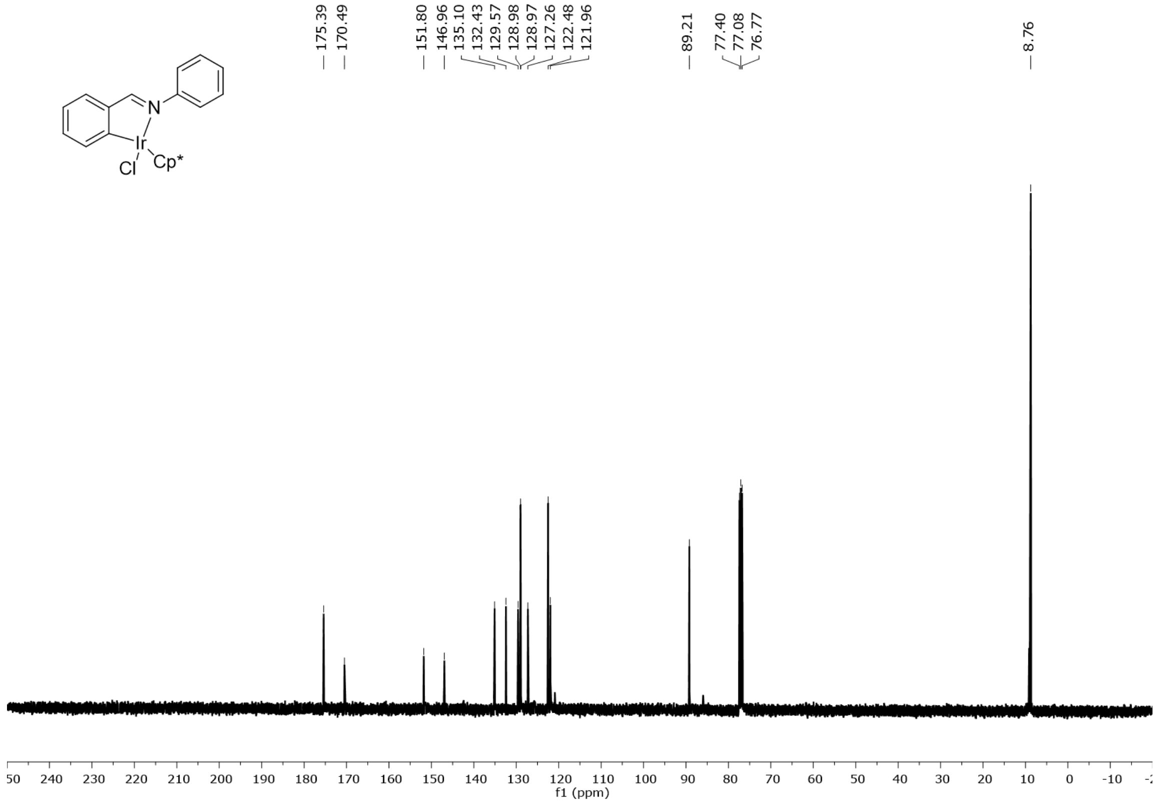
175.39
170.49
151.80
146.96
135.10
132.43
129.57
128.98
128.97
127.26
122.48
121.96
89.21
77.40
77.08
76.77
8.76
N
Ir
Cl
Cp*
50 240 230 220 210 200 190 180 170 160 150 140 130 120 110 100 90 80 70 60 50 40 30 20 10 0 -10
f1 (ppm)

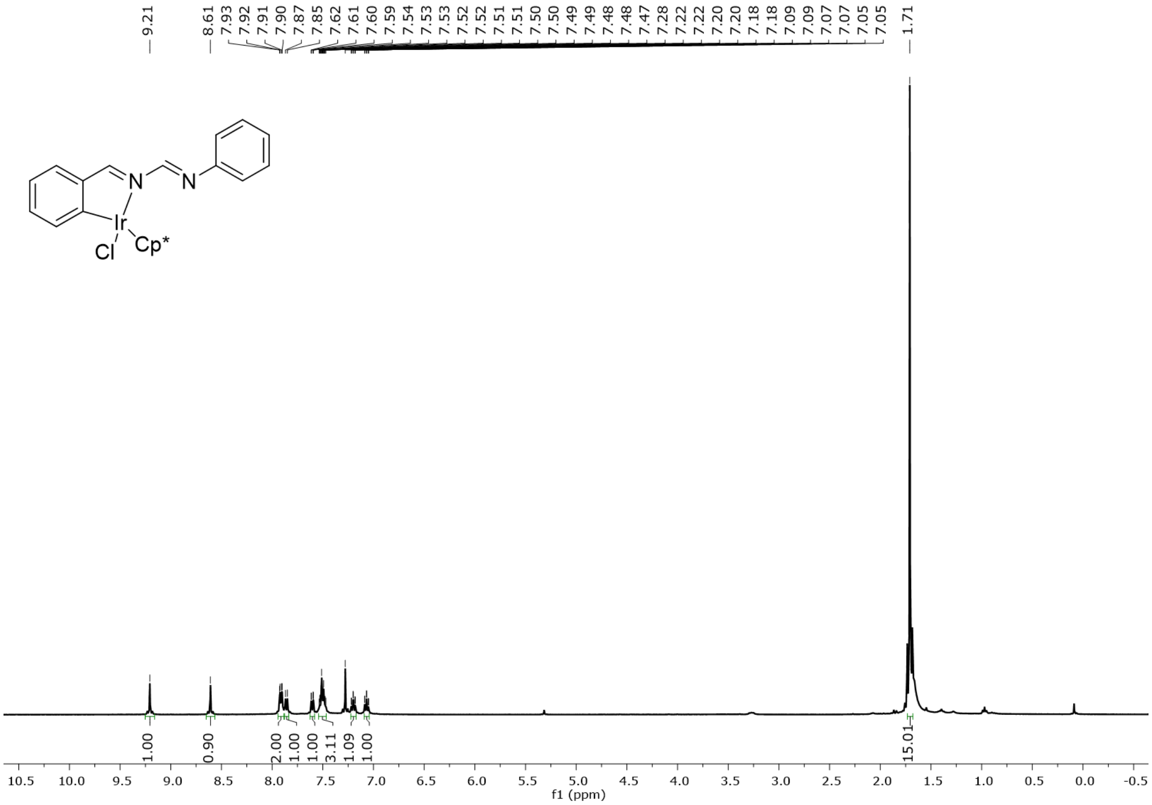
9.21
8.61
7.93
7.92
7.91
7.90
7.87
7.85
7.62
7.61
7.60
7.59
7.54
7.53
7.53
7.52
7.52
7.51
7.51
7.50
7.50
7.49
7.49
7.48
7.48
7.47
7.28
7.22
7.22
7.20
7.20
7.18
7.09
7.07
7.07
7.05
7.05
1.71
N
N
Ir
Cl
Cp*
1.00
0.90
2.00
1.00
1.00
3.11
1.09
1.00
15.01
10.5 10.0 9.5 9.0 8.5 8.0 7.5 7.0 6.5 6.0 5.5 5.0 4.5 4.0 3.5 3.0 2.5 2.0 1.5 1.0 0.5 0.0 -0.5
f1 (ppm)

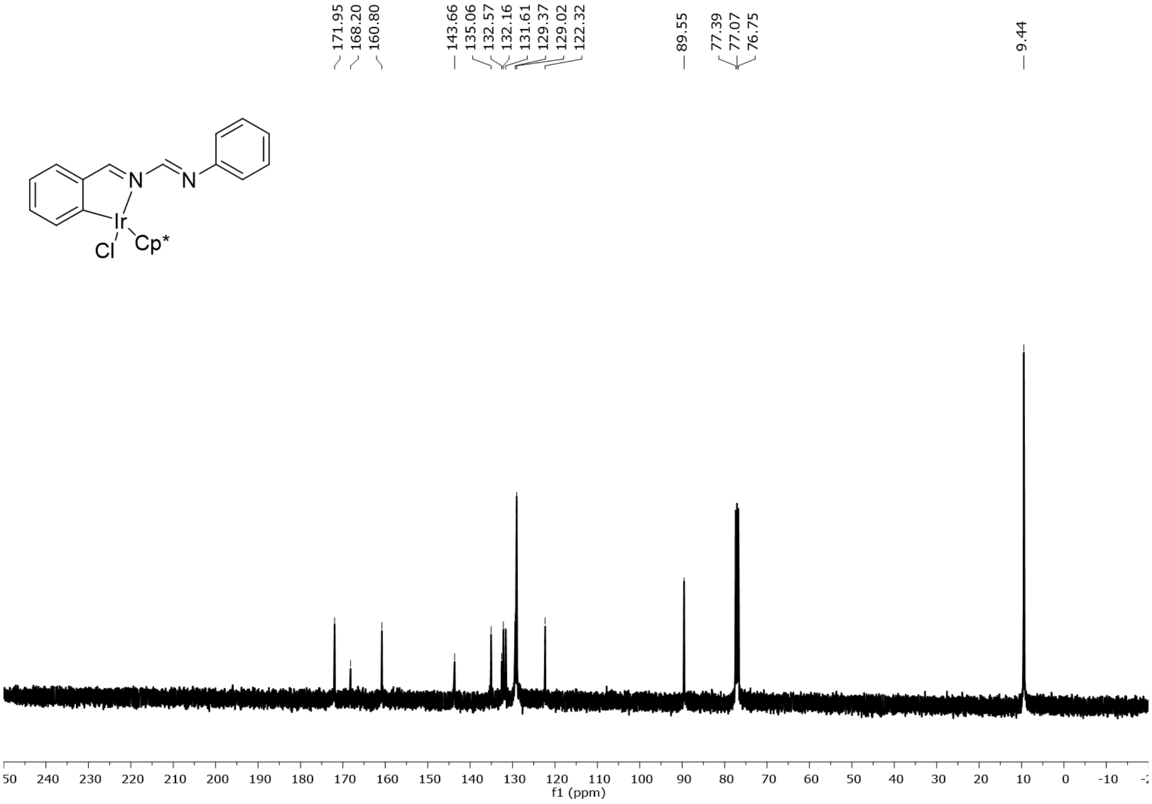
171.95
168.20
160.80
143.66
135.06
132.57
132.16
131.61
129.37
129.02
122.32
89.55
77.39
77.07
76.75
9.44
N
N
Ir
Cl
Cp*
50 240 230 220 210 200 190 180 170 160 150 140 130 120 110 100 90 80 70 60 50 40 30 20 10 0 -10
f1 (ppm)

6. References

(1). Jin, S.; Sakurai, T.; Kowalczyk, T.; Dalapati, S.; Xu, F.; Wei, H.; Chen, X.; Gao, J.; Seki, S.; Irle, S.; Jiang, D., Two-Dimensional Tetrathiafulvalene Covalent Organic Frameworks: Towards Latticed Conductive Organic Salts. *Chem. Eur. J.* **2014**, 20 (45), 14608-14613.

(2). Patra, B. C.; Das, S. K.; Ghosh, A.; Raj K, A.; Moitra, P.; Addicoat, M.; Mitra, S.; Bhaumik, A.; Bhattacharya, S.; Pradhan, A., Covalent Organic Framework Based Microspheres as an Anode Material for Rechargeable Sodium Batteries. *J. Mater. Chem. A* **2018**, 6 (34), 16655-16663.

(3). Davies, D. L.; Al-Duaij, O.; Fawcett, J.; Giardiello, M.; Hilton, S. T.; Russell, D. R., Room-Temperature Cyclometallation of Amines, Imines and Oxazolines with $[MCl_2Cp^*]_2$ (M= Rh, Ir) and $[RuCl_2(P\text{-Cymene})]_2$. *Dalton Trans.* **2003**, (21), 4132-4138.

(4). Yao, Z.-J.; Li, K.; Li, P.; Deng, W., Mononuclear Half-Sandwich Iridium and Rhodium Complexes through C–H Activation: Synthesis, Characterization and Catalytic Activity. J. Organomet. *Chem*. **2017**, 846, 208-216.

(5). Feriante, C. H.; Jhulki, S.; Evans, A. M.; Dasari, R. R.; Slicker, K.; Dichtel, W. R.; Marder, S. R., Rapid Synthesis of High Surface Area Imine-Linked 2D Covalent Organic Frameworks by Avoiding Pore Collapse During Isolation. *Adv. Mater.* **2019**, 1905776.

(6). Peng, Y.; Wong, W. K.; Hu, Z.; Cheng, Y.; Yuan, D.; Khan, S. A.; Zhao, D., Room Temperature Batch and Continuous Flow Synthesis of Water-Stable Covalent Organic Frameworks (COFs). *Chem. Mater.* **2016**, 28 (14), 5095-5101.

(7). https://www.environmentallights.com/15101-wp-blue3528-450-kit.html;

(8). Grunenberg, L.; Savasci, G.; Terban, M.; Duppel, V.; Moudrakovski, I.; Etter, M.; Dinnebier, R. E.; Ochsenfeld, C.; Lotsch, B. Amine-Linked Covalent Organic Frameworks as a Platform for Postsynthetic Structure Interconversion and Pore-Wall Modification. *J. Am. Chem. Soc.* **2021**, 143 (9), 3430-3438.

(9). Gaussian 16, Revision C.01, Frisch, M. J.; Trucks, G. W.; Schlegel, H. B.; Scuseria, G. E.; Robb, M. A.; Cheeseman, J. R.; Scalmani, G.; Barone, V.; Petersson, G. A.; Nakatsuji, H.; Li, X.; Caricato, M.; Marenich, A. V.; Bloino, J.; Janesko, B. G.; Gomperts, R.; Mennucci, B.; Hratchian, H. P.; Ortiz, J. V.; Izmaylov, A. F.; Sonnenberg, J. L.; Williams-Young, D.; Ding, F.; Lipparini, F.; Egidi, F.; Goings, J.; Peng, B.; Petrone, A.; Henderson, T.; Ranasinghe, D.; Zakrzewski, V. G.; Gao, J.; Rega, N.; Zheng, G.; Liang, W.; Hada, M.; Ehara, M.; Toyota, K.; Fukuda, R.; Hasegawa, J.; Ishida, M.; Nakajima, T.; Honda, Y.; Kitao, O.; Nakai, H.; Vreven, T.; Throssell, K.; Montgomery, J. A., Jr.; Peralta, J. E.; Ogliaro, F.; Bearpark, M. J.; Heyd, J. J.; Brothers, E. N.; Kudin, K. N.; Staroverov, V. N.; Keith, T. A.; Kobayashi, R.; Normand, J.; Raghavachari, K.; Rendell, A. P.; Burant, J. C.; Iyengar, S. S.; Tomasi, J.; Cossi, M.; Millam, J. M.; Klene, M.; Adamo, C.; Cammi, R.; Ochterski, J. W.; Martin, R. L.; Morokuma, K.; Farkas, O.; Foresman, J. B.; Fox, D. J. Gaussian, Inc., Wallingford CT, 2016.

(10). Becke, A. D., Density-Functional Thermochemistry. III. The Role of Exact Exchange. *J. Chem. Phys.* **1993**, 98 (7), 5648-5652.

(11). Lee, C.; Yang, W.; Parr, R. G., Development of the Colle-Salvetti Correlation-Energy Formula into a Functional of the Electron Density. *Phys. Rev. B* **1988**, 37 (2), 785.

(12). Hay, P. J.; Wadt, W. R., Ab Initio Effective Core Potentials for Molecular Calculations. Potentials for the Transition Metal Atoms Sc to Hg. *J. Chem. Phys.* **1985**, 82 (1), 270-283.

(13). Wadt, W. R.; Hay, P. J., Ab Initio Effective Core Potentials for Molecular Calculations. Potentials for Main Group Elements Na to Bi. *J. Chem. Phys.* **1985**, 82 (1), 284-298.

(14). Hay, P. J.; Wadt, W. R., Ab Initio Effective Core Potentials for Molecular Calculations. Potentials for K to Au Including the Outermost Core Orbitals. *J. Chem. Phys.* **1985**, 82 (1), 299-310.

(15). Grimme, S.; Antony, J.; Ehrlich, S.; Krieg, H., A Consistent and Accurate ab Initio Parametrization of Density Functional Dispersion Correction (DFT-D) for the 94 Elements H-Pu. *J. Chem. Phys.* **2010**, 132, 154104.
(16). Marenich, A. V.; Cramer, C. J.; Truhlar, D. G., Universal Solvation Model Based on Solute Electron Density and on a Continuum Model of the Solvent Defined by the Bulk Dielectric Constant and Atomic Surface Tensions. *J. Phys. Chem. B* **2009**, 113, 6378−6396.
